# A bipedal mammalian model for spinal cord injury research: The tammar wallaby

**DOI:** 10.12688/f1000research.11712.1

**Published:** 2017-06-15

**Authors:** Norman R. Saunders, Katarzyna M. Dziegielewska, Sophie C. Whish, Lyn A. Hinds, Benjamin J. Wheaton, Yifan Huang, Steve Henry, Mark D. Habgood

**Affiliations:** 1 Department of Pharmacology and Therapeutics, The University of Melbourne, Melbourne, VIC, 3010, Australia; 2 Health and Biosecurity Business Unit, Commonwealth Science and Industrial Research Organisation (CSIRO), Canberra, ACT, 2601, Australia; 3 Centre for Evolutionary and Theoretical Immunology, The University of New Mexico, Albuquerque, NM, 87131, USA

**Keywords:** Tammar wallaby, bipedal locomotion, spinal cord injury, regeneration, supraspinal innervation, marsupial

## Abstract

**Background**: Most animal studies of spinal cord injury are conducted in quadrupeds, usually rodents. It is unclear to what extent functional results from such studies can be translated to bipedal species such as humans because bipedal and quadrupedal locomotion involve very different patterns of spinal control of muscle coordination. Bipedalism requires upright trunk stability and coordinated postural muscle control; it has been suggested that peripheral sensory input is less important in humans than quadrupeds for recovery of locomotion following spinal injury.

**Methods**: We used an Australian macropod marsupial, the tammar wallaby *(Macropus* *eugenii*), because tammars exhibit an upright trunk posture, human-like alternating hindlimb movement when swimming and bipedal over-ground locomotion. Regulation of their muscle movements is more similar to humans than quadrupeds. At different postnatal (P) days (P7–60) tammars received a complete mid-thoracic spinal cord transection. Morphological repair, as well as functional use of hind limbs, was studied up to the time of their pouch exit.

**Results:** Growth of axons across the lesion restored supraspinal innervation in animals injured up to 3 weeks of age but not in animals injured after 6 weeks of age. At initial pouch exit (P180), the young injured at P7-21 were able to hop on their hind limbs similar to age-matched controls and to swim albeit with a different stroke. Those animals injured at P40-45 appeared to be incapable of normal use of hind limbs even while still in the pouch.

**Conclusions**: Data indicate that the characteristic over-ground locomotion of tammars provides a model in which regrowth of supraspinal connections across the site of injury can be studied in a bipedal animal. Forelimb weight-bearing motion and peripheral sensory input appear not to compensate for lack of hindlimb control, as occurs in quadrupeds. Tammars may be a more appropriate model for studies of therapeutic interventions relevant to humans.

## Introduction

Concentrated experimental efforts in adult animals, mainly rodents, have generated substantial information about spinal cord responses to injury and mechanisms behind axonal regenerative failure after trauma. However, translation into clinical outcomes for patients with spinal cord injuries (SCI) has been limited. This is partly because of difficulties in replicating promising results ( Steward *et al.*, 2012), but mostly because of the lack of an accessible model to study therapies aimed at improving bipedal locomotion characteristics in humans. Effective translation of various therapies derived from behavioural studies, mainly in quadrupeds (usually rodents) to patients has so far been unsuccessful. Possible reasons for this have been discussed ( Côté *et al.*, 2017). Most relevant for this study is the accumulating evidence that sensory feedback is important for driving locomotor output in quadrupeds, but not in humans.

There is therefore a need to establish an animal model where naturally occurring bipedal locomotion can be studied. The only eutherian bipedal models available are some species of non-human primates (see Alexander, 2004; Babu & Namasivayam, 2008); however most primates are not truly bipedal, are expensive to study and are not readily accessible. Very few studies of SCI in primate species that are truly bipedal have been published ( Rangasamy, 2013). Macropodid marsupials are the only other major group of mammals that are substantially bipedal. It has been proposed that spinal cord circuits used in bipedal gait in humans and macropods are similar, but evolutionary pressures have resulted in different adaptations as the most effective forms of locomotion ( Baudinette *et al.*, 1992). Macropods employ a hopping bipedal gait and use an alternating pattern of hind limb movements when swimming ( Wilson, 1974). Their locomotion has been studied with respect to mechanics and energetics (e.g. Cavagna *et al.*, 1988), but the circuitry involved in locomotion and development of the spinal cord has only been studied to a limited extent in macropodid marsupials: tammar wallabies ( Comans *et al.,* 1987; Harrison & Porter, 1992), kangaroos ( Watson & Freeman, 1977), quokkas ( Watson, 1971) and potoroos, all of which hop ( Martin *et al.*, 1972). In tammars the forelimb pattern generator, which is important for the first journey to the pouch and is retained for different motor functions in subsequent development, has been described ( Ho, 1997).

A further complication in SCI studies in quadrupedal eutherian species is that the experiments are usually performed at stages of cord development where the environment is inhibitory to growth and therefore regeneration fails to occur naturally. The finding that there is a developmental stage in mammals when the immature spinal cord can naturally repair itself and develop near normally after injury provides a system in which regenerative repair can be studied, especially in marsupials where this stage is uniquely after birth ( Tyndale-Biscoe & Janssens, 1988). This has been shown extensively in two marsupial opossums, *Didelphis virginiana*, ( Martin & Xu, 1988; Wang *et al.*, 1996; Wang *et al.*, 1998 *a*; Xu & Martin, 1992) and *Monodelphis domestica* ( Fry *et al.*, 2003; Lane *et al.*, 2007; Saunders *et al.*, 1995; Saunders *et al.*, 1998; Wheaton *et al.*, 2011). The fact that these results can also be demonstrated in rodents, provided the injuries occur at embryonic stages, shows that regeneration is not merely a marsupial-dependent quality but that the immature mammalian spinal cord possesses substantially greater potential for repair following injury ( Migliavacca, 1930; Saunders *et al.*, 1992).

In this project, we have exploited a unique combination of features of the tammar wallaby: **(i)** like all marsupials, tammars are born at a very early stage of central nervous system development (equivalent to embryonic stages in the rodent) and are therefore accessible for surgery at developmental stages where regeneration might occur without the need for *in utero* interventions; **(ii)** tammars and other macropods use alternating hind limb movements when swimming and bipedal over-ground locomotion.

Here we show that the tammar, similar to the well characterized quadrupedal *Monodelphis*, is also able to re-grow long supraspinal connections following a complete thoracic transection, providing the injury occurs early in development (less than 3 weeks of age). Once these young grew to pouch exit age they were able to hop in a manner similar to un-operated controls; they showed rhythmic alternating swimming hind-limb movements, but with a pattern differing from controls. In contrast, animals injured after 6 weeks of age did not re-grow axons across the site of transection and tended not to survive past the period when they normally start leaving the pouch, indicating that their locomotion was affected and they lacked full hind-limb control. The importance of these findings is two-fold: (i) the study is an independent replication of the fundamentally important biological observation of recovery from spinal cord injury in immature members of a distantly related species and (ii) it establishes the tammar as a model of SCI in a bipedal species. This last is of particular pertinence in the field of spinal trauma research because it offers a new animal model in which interventions to promote repair after SCI can be tested. The use of a bipedal marsupial model allows for the study of behavioural responses, in the presence or absence of spinal cord regeneration, in an animal that maintains upright trunk support for normal posture and locomotion. Thus these animals cannot so easily compensate for dysfunctional hind-limbs by shifting weight support to the fore-limbs, as quadrupeds can. To understand how these animals function following SCI would make a substantial contribution to developing future therapies suitable for human patients with paraplegia.

## Methods

### Ethical approval

The tammar wallaby *(Macropus eugenii)* pouch young (PY) were sourced from adult females derived from the CSIRO Wallaby Colony maintained at Crace, Canberra, Australia. In this colony, animals are held in open grassy yards and provided with supplemental ewe and lamb food pellets and water *ad libitum*. All procedures were approved by the CSIRO Wildlife and Large Animal Animal Ethics Committee (approval number, 13-05) following National Health and Medical Research Council (NHMRC; Australia) guidelines.

A complete spinal transection results in initial paralysis of the hind limbs and loss of sensation from the hindlimbs and lower trunk. However, at the time when the operation was done the young are at a very immature stage of development, are highly dependent on the protective environment of their mother’s pouch in which all nutritional and thermoregulatory needs are provided for up to 180 days ( Tyndale-Biscoe & Janssens, 1988). The pouch young is permanently attached to its specific teat at this age, their nutritional state is not disrupted. The young remain in the pouch for the duration of the experiment and so are protected from the external environment. Developmental changes in the electroencephalogram and responses to toe pinching have been previously monitored in tammar young. EEG activity was not recorded until after 120 days, and responses to toe pinching were shown to be minimal prior to 127 days of age ( Diesch *et al.*, 2010). In the tammar wallaby the eyes open at 140 days of age. In the present study surgery was done at or before 60 days of age and well before eye opening (140 days). Thus distress due to the spinal transection would be minimal or even non-existent; however, all operative procedures were carried out under anaesthesia.

### Experimental animals

Two types of experiments were conducted: **(i)** animals at four different postnatal-day (P) age groups: P7 (P4−15), P20 (P18−23), P40 (P39−45), and P60 were used for shorter time experiments that investigated re-growth of neuronal fibres across the site of transection and **(ii)** animals transected at P7–15 and P40–60 that were held until early pouch exit (around 200 days) in order to study their locomotor abilities. Details of numbers of animals and their ages are listed below.

### Surgery (spinal cord transection)

Adult female tammars with PY were caught, transferred into hessian sacks, and held in a holding facility. PY were removed from the pouch, and their head length and body weight recorded prior to surgery for age determination ( Poole *et al.*, 1991). PYs of both sexes were used (see below). They were anaesthetized by exposure to inhaled Isoflurane (3–4% in oxygen). A cotton ball was soaked in Isoflurane and placed in a small glass jar. The jar was placed over the PY’s snout until there was no evidence of muscle reflexes in response to a peripheral stimulus.

At each age, the available PY were divided into two groups: an experimental group (n = 4–11) that had their spinal cord transected and a control group (matched numbers), which were not injured. The sex of tammar PYs cannot be determined in the first week of life. In the case of older PYs, the sex of the animals was not known to the person allocating the animals to the two groups or to the person conducting the operations.

Under sterile conditions, the PY was positioned on its stomach over a roll of gauze to elevate the thoracic spine. A small skin incision was made over the dorsal aspect of the lower thoracic spine spanning two vertebrae. The spinal cord of each experimental animal was exposed at the lower thoracic level via gentle muscle dissection between two vertebrae. The spinal cord was cut completely at the approximate level of T10 using a fine ophthalmic blade, as described previously for *Monodelphis domestica* ( Fry *et al.*, 2003; Lane *et al.*, 2007; Wheaton *et al.*, 2011).

The wound was closed using fine monofilament sutures and sealed with tissue adhesive. The PY was warmed using body heat until it had visibly recovered from anaesthesia and was then placed back into the mother’s pouch. Approximately 30 minutes later the mother was put under light anaesthesia by the use of inhaled Isoflurane (3% in oxygen). The pouch was held open to expose the teats and, using small forceps, the active teat was placed into the PY’s mouth. The PY was observed until it was apparent that the young was securely attached. The level of anaesthetic was turned down to 0% in oxygen and the mother placed on her side in a hessian bag and observed until recovery from anaesthesia was apparent. In each age group some PYs (randomly selected by a person not involved in surgery) were removed from the pouch, terminally anaesthetized and fixed for examination of completeness of the lesion both macroscopically and microscopically. In the P7 group 4/4 cuts were complete. In the P20 age group, 3/4 (2 males and two females) were complete, while in the P40 age group all five (two males, three females) were complete. Only one (female) PY at P60 was tested and the transection was complete. Mothers were then released into their respective yards. PY from P7 and P20 age groups were observed 24 hours after the surgery to confirm that they were in good condition and still attached to the teat. They were then checked again 4–5 days later. P40 and older PY were checked after 4–5 days, but were largely left unobserved to avoid handling stress and to increase the chance of their survival.

### (i) Labeling experiments: Re-growth of axons across the site of transection

Four experimental age groups were used for retrograde labeling of axons growing through the transection site. PY at ages of P4–7 (“P7 group”, n=4), P18–21 (“P20 group”, n=4), P40–45 (“P40 group”, n= 8) and P60 (n=2) received a complete spinal cord transection in the lower thoracic region (T10), as described above. Age-matched controls were also included. Numbers of animals used in these experiments are listed in Table 1. Retrograde tracers were injected into the spinal cord at different times to label axons extending through the injury site.

**Table 1.** Numbers of animals in each experimental group/out of total used for the labeling experiments. Details of animals’ sex included in the legend to Table 2. P, postnatal day.

AGE	INJURED, n	CONTROL, n
P7	4/11	4/8
P20	4/8	4/5
P40	4/8	4/4
P60	2/8	2/8

Note that animal losses were mostly due to the difficulties in their survival after the second surgery (injections of Fluoro-Ruby), as at those stages the animals were older.

**Figure 1.**
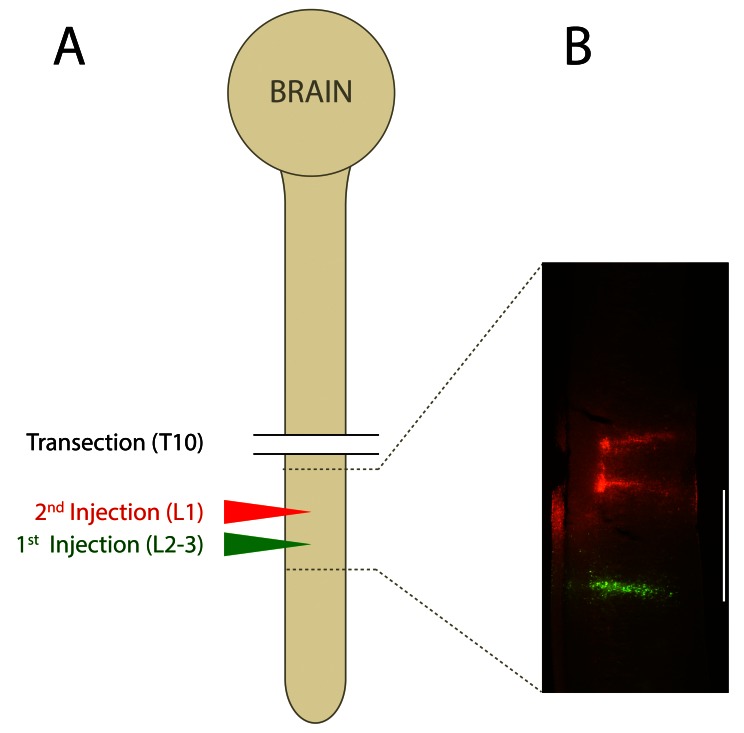
Illustration of the injection protocol into the spinal cord of pouch young tammars. **A** is a diagram representing the brain and the cord with levels of transection at thoracic (T) segment 10 indicated by lines and sites of first injection of Oregon Green at lumbar segment L2–3 shown in green while the second injection of Fluoro-Ruby into L1 shown in red. A micrograph of the spinal segment indicated by broken lines and taken under the fluorescent microscope is shown in **B**.

A diagram illustrating the injection protocol is shown in Figure 1A. Briefly, immediately following transection, the upper lumbar region of the spinal cord was exposed and 0.25μL of Oregon Green (Molecular Probes) tracer (25% weight/volume in 2.5% Triton X-100, 0.1M TRIS buffer; pH 7.6) was injected into each side of the cord at the approximate level of L2–L3 using a fine glass pipette and gentle pressure. Animals in the control group were not injured, but received an injection of Oregon Green in the similar upper lumbar region of the spinal cord. This label was injected at this early time in order to test the completeness of the cut, as any green labeling rostral to the transection would indicate an incomplete transection ( Fry *et al.*, 2003).

After a period of 1–2 months (time adjusted for the age differences between the groups) a second tracer (Fluoro-Ruby, Molecular Probes) was injected rostral to the first injection, but still caudal to the site of transection, in order to label any fibres that had re-grown through the injury site ( Fry *et al.*, 2003). For this procedure, PY were removed from the mother, as described above, and their body weight and head length recorded. Animals were anaesthetised with 3% isoflurane in oxygen and the upper lumbar vertebrae exposed at the approximate level of L1–L2. The retrograde axon labeling probe, Fluoro-Ruby (25% weight/volume in 2.5% Triton X-100 0.1M TRIS buffer; pH 7.6) was injected into both sides of the cord (0.5μL each injection). The wound was closed and PY were returned to their mother’s pouch, as described above.

After the Fluoro-Ruby injection, all young were held for a further 14–21 days. Young were retrieved, final body measurements recorded and the young were terminally anaesthetized (overdose of isoflurane), perfuse-fixed with 4% paraformaldehyde (PFA), and post fixed for an extra 24 hours in 4% PFA.

***Processing of tissue samples for fluorescent analysis.*** Following fixation in PFA, brains were removed from the skull and the spinal cords were dissected out of the vertebral column (from about T1 level to L6) and individually embedded in high gel strength 4% Agar (Sigma-Aldrich). Each spinal cord was divided into blocks of about 2–3cm, including the site of injury and both injections, and cut into serial longitudinal sections of 100μm thick on a vibrating microtome (Leica). The brainstems were serially sectioned into 100μm coronal sections. All sections were mounted on glass slides using fluorescent mounting medium (DAKO) and kept at 4°C covered with foil to restrict light exposure. All sections were viewed with an Olympus BX50 fluorescent light microscope with filters specific for Fluoro-Ruby, Oregon Green or triple filter. Digitized photographs with embedded scale bars were taken using an Olympus DP70 camera attached to the microscope.

Following the initial screen of the tissue, specific criteria were set for animals to be further included in the study:

(a) Spinal injections needed to be successful: Oregon Green injected immediately after injury and Fluoro-Ruby injected 30–60d later both had to be visible in the lumbar spinal cord at the correct spinal level. This applied to both the injured and uninjured control animals ( Figure 1B).
(b) Evidence of completeness of the transection: in animals with complete spinal injuries, no Oregon Green cells could be detected rostral to the injury site (both in rostral spinal cord and the brainstem).
(c) In control animals, both labels were clearly visible in the rostral spinal cord and brain stem nuclei.

Any animal that failed one or more of these criteria was removed from further analysis. The final animal numbers used in this part of the study are shown in Table 2.

**Table 2.** Summary of tracing experiments

AGE		SCI			CONTROL		
SCI	analysis	n	green	red	n	green	red
P7	P90	2/4	0	4, 158	2/4	50, 975	1040, 4703
P20	P60	2/4	0	132, 852	4/4	19, 26, 211, 388	75, 1920, 2323, 4963
P40	P80	3/4	0	0	2/4	108, 431	1642, 2293
P60	P90	1/2	0	0	1/2	274	2112

Age refers to the mean group age (described in Methods) at the time of spinal cord injury ( **SCI**) and injection of the Oregon Green tracer ( **green**); **analysis** refers to time of termination of experiment after injection of the Fluoro-Ruby tracer ( **red**). n = number of animals with successful outcomes/out of total attempted following surgery and both injections in each age group. Please note that to be counted as a successful outcome selection criteria were applied as described in Methods. Numbers for all labeled neuronal cell bodies counted in brainstem nuclei are shown for individual animals (0 = no positive labeled cell bodies detected). In the P7, P20 and P40 groups, numbers of males and females were similar; at P60 both animals were males. P, postnatal day.

***Analysis of axonal labeling in the cord and neuronal labeling in the brainstem.*** Spinal cord: Every longitudinal section through each cord spanning the entire length from about T1 to L6 was examined under the fluorescent microscope and the distribution of green- and red-labeled fibres and cell bodies was noted. This was done to establish that injections were successful and in the right spinal segment. In the case of transected animals, the lack of green labeling in the rostral segment was used as a definitive criterion that the original cut was complete.

Brainstem: All serial sections from brainstems of transected and control PY were investigated under a fluorescent microscope and all cell bodies containing fluorescent labeling were counted and tallied. A note was taken of how many cells were labeled with either green, red or included both fluorophores (yellow).

### (ii) Long-term experiments: Locomotor abilities at pouch exit

**Figure 2.**
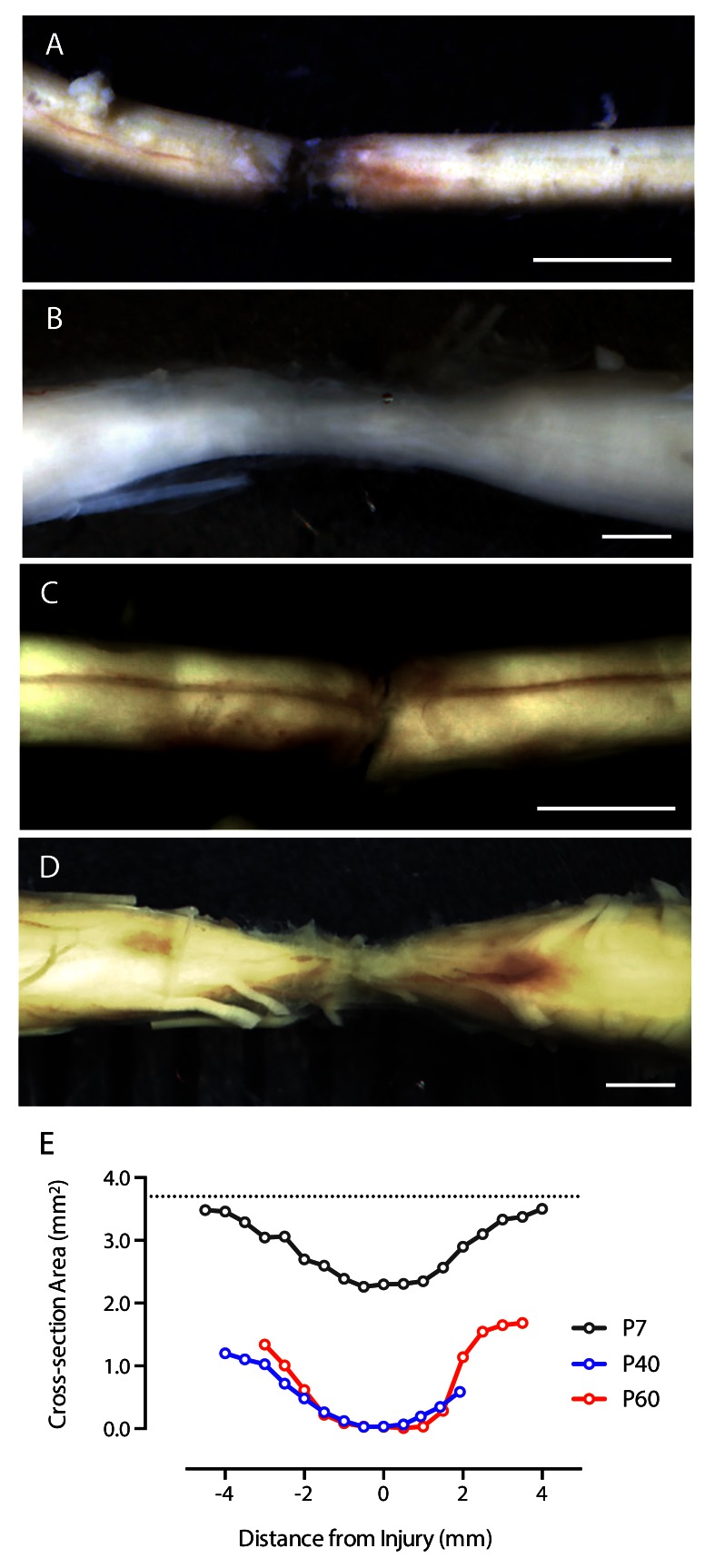
Spinal cords of tammars after a complete transection at T10. **A–D** are whole-mounts of the cords taken immediately after dissection from a fixed tissue while **E** shows changes in cross-sectional area at the injury site of P7, P40 and P60 transected cords when measured at P160 **. A** is immediately after surgery on a pouch young at P13; **B** is a cord from a P12 transected animal that was allowed to grow until P160; **C** is immediately after surgery on a P40 animal; **D** is a cord from a P40 transected pouch young taken at P160. The rostral end is to the left. Scale bar is 1mm. **E** represents the cross area (in mm ^2^) of H&E stained sections. 10mm length of cord enclosing the injury site was cut into consecutive 5μm thick transverse sections, every 500μm section stained with H&E and the spinal cord area measured (see Methods). Sections were centered on the middle of the injury (0), rostral (negative numbers) is to the left while caudal (positive numbers) is to the right. Hashed line at the top illustrates the expected P160 cross-sectional area of the cord if the injury were not present.

Two age groups of animals were used for long term experiments, in which locomotor abilities of PY at pouch exit (P190–200) were analyzed: P7–15 (P7; n=8), P39–45 (P40; n=4) and P60 (n=2). Age-matched controls were also included (n=3. Spinal cord transection procedures and animal care were performed as described above. To assess completeness of the spinal transection, cords from random PY were collected immediately after surgery. Four out of five cords investigated immediately after transections at P7–15 had complete cuts and five out of five collected immediately after transections at P39–45 were complete. Figure 2 illustrates two such cords collected immediately after transection at P7 and at P40.

***Long-term animal care and locomotor testing.*** All animals (transected and age-matched controls) were measured (weight and head length; Poole *et al.*, 1991) and returned to the mother’s pouch; their ability to move hind limbs was assessed from around P140, with a more comprehensive testing at around P150–160. This included taking video recordings of PY efforts to stand up, move their hind limbs while supported, with special emphasis on rhythmic kicking. After these tests, the young were returned to the pouch and mothers were placed in individual wire-enclosed pens in the open yards, as at this stage if the injured young fall out of the pouch they may not be able to return and are potentially a target to bird predators. Once established in individual pens, the females were monitored every second day for the presence of the young in their pouches. At this stage (days 140–160), we observed greatest losses of the young: most of the animals injured at P40 were found outside the pouch and had difficulty standing and were unable to return to the pouch unaided. Therefore, experiments for all animals operated at P40 and P60 were terminated by terminal anaesthesia at around P150–160. Animals operated at earlier ages were left with their mothers until P190, when the young start to periodically leave the pouch ( Tyndale-Biscoe & Janssens, 1988) and are able to hop in a mostly coordinated fashion. Further testing was performed on this group of PY, as described below. For all observations of locomotion, including hopping and swimming (see below) the observer was blinded to whether the animals were controls or operated.

***Over ground hopping test.*** A runway (0.6m wide × 2.5m long) was erected against a solid wall with the parallel wall (0.6m high) made of clear Perspex. Each test young was placed at one end of the runway and encouraged to hop towards the other end where the mother was placed held in a hessian bag. When the young vocalized so did the mother and the young would generally hop towards the sound. Markings at 100cm intervals were made on the back wall and video footage was used to determine normality of movements compared to uninjured controls. In addition, the young were also placed in an open field inside the room and allowed to hop of their own accord whilst being video recorded. The animals would typically hop towards and follow the experimenter. Each animal was observed for its ability to hop in a straight line, weight bearing use of fore and hind limbs and limb coordination.

***Swimming test.*** A clear, glass tank (1.8m long/0.6m wide/0.8m deep) was constructed and used in this test. Water temperature was kept at approximately 27°C. Young tammars at about P190–P200 were placed at one end of the tank and video recordings of their swimming were taken. Animals were not able to touch the bottom of the pool with their hind limbs. Video footage was examined for the animals’ ability to use their hind limbs (evidence of supraspinal innervation, Wheaton *et al.*, 2011), hind limb extension, fore-hind limb coordination and tail movements. However due to losses of young transected at ages older than P40, we were only able to record swimming of animals transected at P7–15 together with age-matched controls.

***Morphology of the injury site.*** At the end of the locomotor testing, animals were terminally anaesthetised by an overdose of inhaled isoflurane, transcardially perfuse-fixed with PFA and spinal cords and brains collected, as described above. A segment of cord (10mm) containing the injury site was dissected out and post fixed in Bouin’s fixative for detailed morphological analysis in paraffin sections.

Tissue fixed in Bouin’s fixative for 24 hours was rinsed in distilled water and washed in 70% ethanol until clear, followed by dehydration in graded alcohols up to 100% ethanol. Tissue was placed in chloroform for 24 hours, followed by 60°C in paraffin wax and infiltrated under vacuum before embedding in warm paraffin wax in the desired orientation.

Paraffin embedded spinal cord tissue was serially sectioned (5μm) in the transverse plane on a microtome (Leica). Ribbons of ten consecutive sections were mounted on each gelatin-coated glass slide and left to dry over several days in a warm oven (36°C).

In order to perform histological and immunohistochemical analysis, every tenth slide (about 500μm apart) was stained for each method in order to obtain an overview of each cord. General histology sections were stained using Mayer’s Haematoxylin and Eosin Y (H&E), myelin was detected using Luxol Fast blue (LFB), and axonal neurofilaments and glia cells were detected using specific antibodies and PAP method (see below).

H&E: Selected slides were de-waxed and re-hydrated through graded ethanol solutions and washed in tap water. They were immersed in haematoxylin (0.2%; Sigma) for 20 min, washed in tap water and stained with eosin (0.1%; Sigma) for 2 minutes, followed by another wash in tap water. Finally sections were dehydrated through increasing concentrations of ethanols and histolene (Fronine) and finally coverslipped with Ultramount Mounting Medium (Fronine).

LFB: Selected slides were de-waxed, but not rehydrated. Instead slides were brought to 100% ethanol and immersed in LFB solution (0.1% w/v; Sigma; 10% acetic acid dissolved in 95% ethanol) overnight at 60°C. Tissue was rinsed in 95% ethanol and differentiated in 0.05% solution of lithium carbonate in water followed by 70% ethanol until white and gray matter could be easily distinguished. Sections were then permanently mounted as described above.

Immunohistochemistry: Following de-waxing and rehydration (see above) selected sections were washed in phosphate-buffered saline (PBS; 0.2M, pH 7.4) with 0.2% Tween20 (Sigma) and incubated with Peroxidase- and Protein- Blocking Solutions (both from DAKO) for 2 hours each. Sections were incubated with primary antibodies: rabbit anti-glial fibrillary acidic protein (DAKO, Z0334 1:200 dilution in PBS) or mouse anti-SMI 312 (pan-axonal neurofilament marker; Steinberger, SMI312; diluted 1:200 in PBS) overnight at 4°C. The next day, slides were washed in PBS/Tween20 and incubated with corresponding secondary antibodies (swine anti-rabbit, Z0196 or rabbit anti-mouse, E0464, both from DAKO) in 1:200 dilution in PBS for 2 hours at room temperature. After several washes in PBS/Tween20 slides were incubated in appropriate peroxidase-anti peroxidase (PAP) complexes: rabbit PAP (P1291, 1:200, Sigma) or mouse PAP (B650, 1:200, DAKO) again for 2 hours at room temperature. Following washes, slides were developed by a reaction with DAB (DAKO). Once the reaction was developed (about 5 minutes) slides were dehydrated and mounted as above.

Histological analysis: One section from every stained slide was photographed under 10× magnification (Olympus BX50 microscope with DP70 digital camera). The area of stained cord was quantified using ImagePro Plus software (version 4.5.1.22). The perimeter of the section was outlined and automated measurement function applied. For comparison between cords obtained from animals injured at different ages, the obtained areas of all sections were plotted against their position along the length of the corresponding cord segment relative to the center of the injury site ( Figure 2E).

## Results

### Animal survival

In the group of animals used for the labeling experiments, the outcomes of each surgery and animal survival varied between age groups. All animals survived the first surgery and were responsive when placed back into the pouch and re-attached to their teat; however, the likelihood of their survival in the time between the first and the second surgery and then until the final collection appeared to decrease with age at the time of the transection. The mothers of those that did not survive were found with pouches empty during checks undertaken before the second surgery was scheduled. Animals from the two older operated groups did not fare as well and their losses were much higher. The numbers of animals that survived and were analyzed are recorded in Table 1; not all animals had successful injections or complete cuts. Numbers of animals that conformed to inclusion criteria listed above (successful injections, completeness of the cut) are listed in Table 2. There was no difference in the survival rates between the two sexes.

In the two age-groups of animals that were used in the long-term experiments to assess their locomotion after SCI, all animals injured at P7–15 (n=8) survived until their final testing, while out of 4 transected at P39–45 and two transected at P60, all survived until P150, but one from each group died shortly after. However, due to increased risk of losing these animals, short behavioural testing was conducted at about P150, and all but one of the surviving animals from the two older groups were culled at this time for morphological examination. The one retained young was re-tested at P160 followed by terminal anaesthesia and collection of tissue for morphology.

### Analysis of axonal labeling in the cord and neuronal labeling in the brainstem

**Figure 3.**
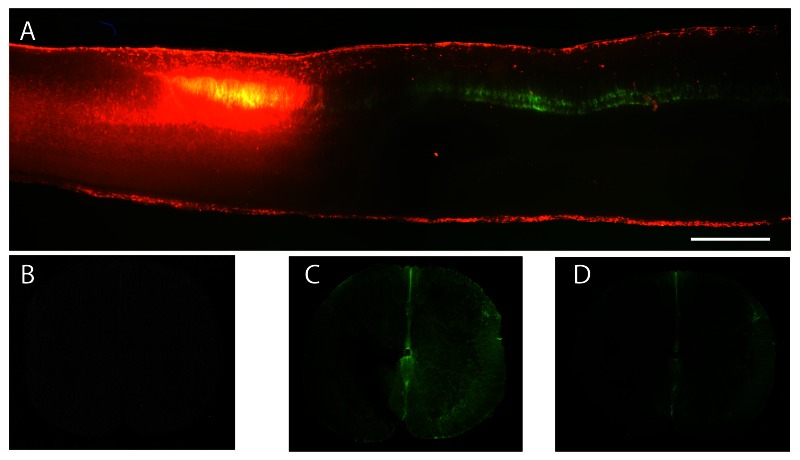
Sections through control and transected P23 spinal cords following injection of fluorophores. ( **A**) P23 control (not transected) spinal cord injected at the same level as that in operated animals and examined 30 days later; this illustrates the longitudinal appearance of the cord from an animal that was included in the study of counting labeled neurons in the brain stem. Rostral end to the left. ( **B–D)** 50μm thick vibratome cut transverse sections of P23 transected spinal cord injected with Oregon Green caudal to lesion immediately after lesioning and collected 1.5h later. Note lack of Oregon Green rostral to lesion ( **B**), but presence of the fluorophore caudal to the lesion ( **C and D**). ( **C**) is in close proximity and ( **D**) is further caudally. Lack of rostral labeling indicates that the lesion was complete. Scale bar is 500μm.

***Spinal cord.*** Every longitudinal spinal cord section for each embedded cord was examined under the fluorescent microscope and distribution of green and red labeling was noted. As described above, these sections spanned approximately from the T1 to L6 spinal segments, encompassing both the injury and injection sites. In control cords from all age groups, green and red cell bodies and fibres were visible along the entire length of the cord, including the most rostral segments. However, no double labeled cell bodies were observed in any of the cords. An illustration of successful labeling following both injections in the cord is presented in Figure 3A.

**Figure 4.**
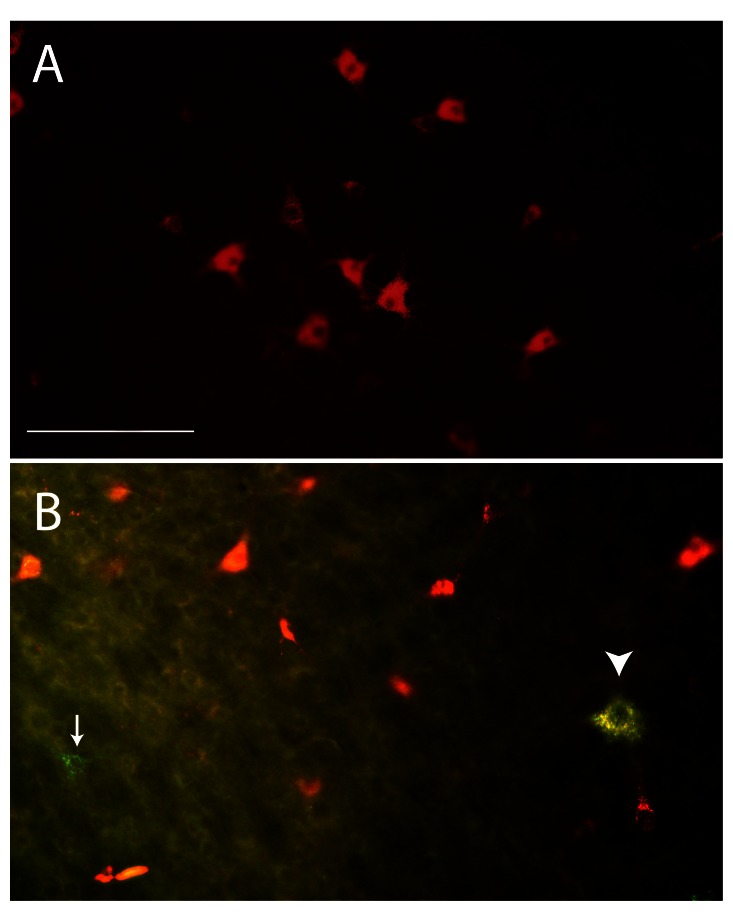
Examples of labeled neurons in the brain stem. Gigantocellular reticular nucleus of a P7 transected ( **A**) and age-matched control ( **B**) pouch young. Only red-labeled cells could be detected in transected animals indicating re-growth of supraspinal axons across the site of injury. Lack of green labeled cells indicated that the transection was complete ( **A**). In control animals both labels could be detected with many more red-labeled than green (arrow), or double-labeled (arrowhead) neurons visible ( **B**). Images taken under triple filter specific for fluorescein and rhodamine. Scale bar is 200μm.

In all animals with spinal transections that were included in this study, no green labeling was visible in the segment rostral to the injury ( Figure 3B), but was clearly visible caudal to the injury site ( Figure 3C and Figure 3D). If green label was detected in the rostral segment of the cord, it was an indication that the injury was not complete and the animal was removed from the study (see Methods). This only occurred in one animal injured at P4 and in one injured at P60. The red fluorophore (2 ^nd^ injection) could be detected in spinally transected animals from the two younger groups, but not in animals injured after P40. This observation was confirmed by the results obtained from the analysis of labeled neuronal cell bodies in the brainstem regions of control and transected animals ( Figure 4). Control animals all had labeled axons detected in the rostral end of their cords and cell bodies labeled in the brainstems ( Figure 4). This indicates that following a complete transection in the thoracic region up to 3 weeks of age, spinal axons were able to span the injury site, but this did not occur if the transection was performed in animals older than 6 weeks of age.

***Brainstem.*** Agar-embedded brainstems were sectioned in the coronal plane on a vibrating microtome. They were examined under a fluorescent microscope fitted with filters specific to each fluorescent probe. The numbers of neuronal cell bodies positive for Oregon Green (green) and Fluoro-Ruby (red) were counted, as well as those containing both labels (yellow).

In SCI animals, the presence of Fluoro-Ruby labeling within cell bodies in the brainstem of pouch young indicated the regrowth of axons across the lesion site ( Figure 4A). However if Oregon Green–positive cell bodies were also detected, this indicated that the transection was not complete and these animals were not used in further analysis (see above). Only one animal injured at P4 was removed from the study due to incomplete transection. Presence of both labels in brainstem nuclei in control animals indicated successful injections in these animals ( Figure 4B).

Five of the eight P40 injured young were lost soon after the second injection and are thus not included in Table 2, which shows final numbers of animals that were analyzed. As can be noted in Table 2, in control animals, the numbers of Oregon Green and Fluoro-Ruby–labeled cell bodies respectively were of similar order of magnitude in all three age groups, indicating that the volume of injected dye was relatively adjusted to the size of the growing spinal cord ( Fry *et al.*, 2003). In all cases, the numbers of Fluoro-Ruby-positive neurons were an order of magnitude higher than Oregon Green-positive neurons, which is most likely a reflection of older (bigger) cords at the time of the second injection.

There was a large variation in numbers of labeled neurons even within one age group; this was most likely due to difficulties in injecting small volumes of the fluorescent probe into the cords, especially in SCI animals, where cords were often narrower near the injury site. Nevertheless the data clearly show that following a complete T10 transection in animals up to 3 weeks of age (total n=4), there is substantial morphological repair across the site of injury, as demonstrated by the presence of red-labeled neurons in the brainstems of spinal animals ( Figure 4A). In brains of two animals injured at one week of age and two animals injured at around three weeks of age, up to 800 Fluoro-Ruby-labeled neuronal cell bodies were counted. Additionally in the P7 and P20 age group, the numbers of Fluoro-Ruby cell bodies were between 5−20% of those detected in un-injured age-matched controls. In the three animals obtained from the group injured at around 6 weeks of age and one injured at P60, no Fluoro-Ruby-labeled cell bodies were detected in the brainstem ( Table 2), indicating that there was no re-growth of axons across the site of transection in these older pouch young.

Double labeled neuronal cell bodies were also detected in brain stems of all control animals and were occasionally as high as 80 ( Figure 4B). There were no double-labeled neurons in any of the brains from SCI animals, thus confirming the completeness of the spinal transections.

### Morphology of the injury site

**Figure 5.**
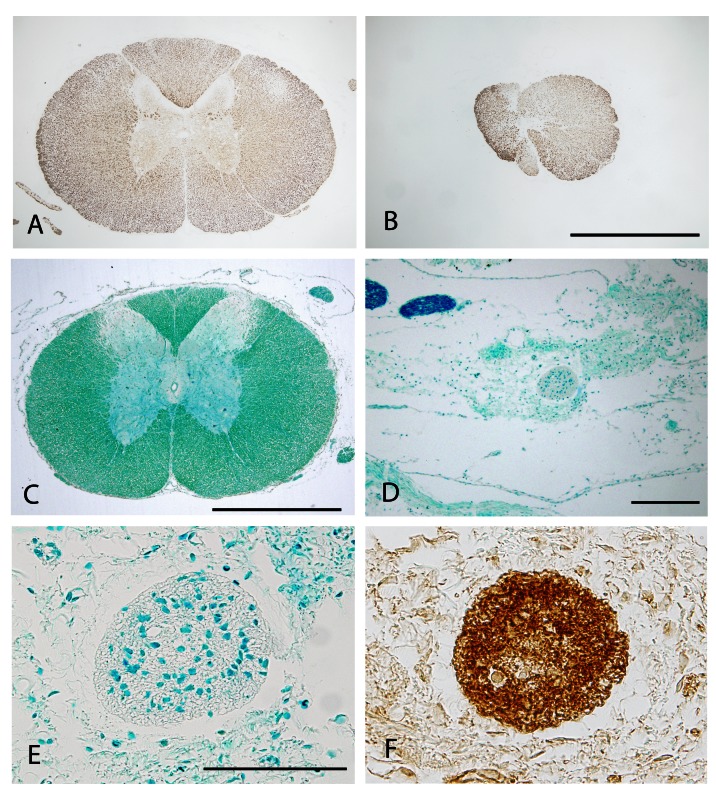
Transverse sections though the site of injury in transected (B, D, E, F) and age-matched control (A, C) spinal cords. All sections are from animals at around P160. **A** and **B** are immunostained with antibodies to neurofilament. **C, D** and **E** are from sections stained with Luxol Fast blue to detect myelin. **F** is stained with antibodies to GFAP. **B** is a section from the middle of the injury performed at P13. **D, E** and **F** are from the middle of the injury site made at P60. Note substantial neurofilament immune-positive tissue present in **B** indicating regrowth of axons after transection at P13. Following transection at P60 myelination could not be detected at the lesion centre ( **D** and **E**). Instead very strong GFAP staining in **F** indicates that the site of transection performed at P60 was now filled with glia cells, forming a scar. Scale bar is 500μm in **A, B** and **C;** 200μm in **D;** 100μm in **E** and **F**.

The difference in the age-related morphological repair between the two groups of animals observed short-term (above) was confirmed by immunohistochemical analysis of transverse sections through cords obtained from animals used in the long-term experiments described below. Sections through the centre of the initial spinal transection performed at either P7 or P40 (and age-matched uninjured controls) and analysed at P150-180 are illustrated in Figure 5. Measurements of the cross-sectional area of transverse sections along the length of different cords are illustrated in Figure 2E above.

The control cords at P160 ( Figure 5A) show the usual well defined structure of characteristic white and grey matter typical of the thoracic mammalian cord and white matter, as defined by LFB staining ( Figure 5C). Following spinal transection at P7, the cords were able to repair themselves to a degree showing strong immunoreactivity for neurofilament, indicating that neurites were able to span the site of the injury. However the overall morphology of the cord is disturbed and grey matter is absent, but some white matter is defined ( Figure 5B). In contrast to the cords of injured animals from the younger (P7–P20) permissive group, cords from the older (P40−P60) non-permissive group, when analysed at P150, showed no LFB staining ( Figures 5D and E) or neurofilament immunoreactivity at the transection site. Instead, a loosely defined fibrous connective tissue could be observed defined by a very strong immunopositive reaction with antibodies to GFAP ( Figure 5F), indicating a likely glial scar, presumably involving mainly A1 astrocytes given the lack of neurite outgrowth ( Liddelow *et al*., 2017).

Estimations of the cross-sectional areas of cords injured at P7, P40 and P60 are illustrated in Figure 2E. Each point on the graph represents measurements from one section. As can be seen in this figure, a significant tissue deficit in all three transected cords was detected. However, the tissue that was present in the P7 injured cord was considerably larger than either of the cords injured in older animals.

These data and observations confirm that following a complete transection of the spinal cord, tammars up to three weeks of age are capable of significant morphological repair (Fluoro-Ruby-labeled axons in the rostral cord and labeled neurons in the brain stem indicate neurites crossing the site of transection). This process of morphological repair did not occur in animals injured from about six weeks of age.

The next set of experiments aimed at establishing if morphological repair translated into functional recovery after SCI.

### Long-term functional recovery

**Figure 6.**
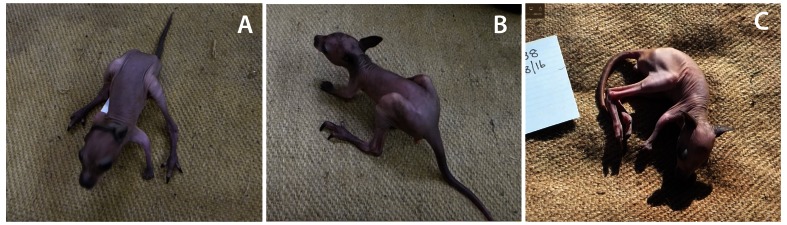
Tammar wallaby pouch young at around 160 days (P150–160). Control ( **A**), P7 ( **B**) and P40 ( **C**) were removed from their mothers’ pouches and videos of their ability to remain upright recorded. Note that controls (n=3) and P7 group transected animals (n=8) were able to remain upright using the support of all four limbs while the P40 group operated animals (n=3) were unable to do so.

Following complete thoracic spinal transection at different ages (see Methods), all surviving animals were tested at two ages: around P150–160 and P190–P200. The aim was to keep the animals until such time that they would begin to leave the pouch (around P200), be able to stand and hop (after P180) and swim when placed in a water tank (also after P180). However, since we noticed in our previous experiments that most losses of PY operated after P40 occurred around the time of P140–P160, we instead conducted the first test, consisting only of recording the animals’ ability to use their hind limbs in a manner similar or dissimilar to un-operated age-matched controls, at this age. At around P150–160, the control (n=3, two females, one male) and P7–15 transected (n=8, four females, four males) PY could kick their hind-limbs in a coordinated manner while supported, but the young operated at P40 (n=2, one female, one male) and older (P60, n=1, male) were less able to do so. In addition control and PY injured in the first three weeks of life were able to remain upright and stand using all four limbs for support, while animals operated after P40 were not able to maintain an upright posture ( Figure 6). As P40 operated animals exhibited such reduced mobility, and most in fact were lost after about 150 days of life, these animals were terminally anaesthetized and their brain and spinal cord fixed for morphological examination together with n=3 PY from the P7–15 group at this point. One P60 operated PY was allowed to remain in the study, but was rejected by the mother shortly afterwards and was therefore terminated for morphological examination of the cord.

**Figure 7.**
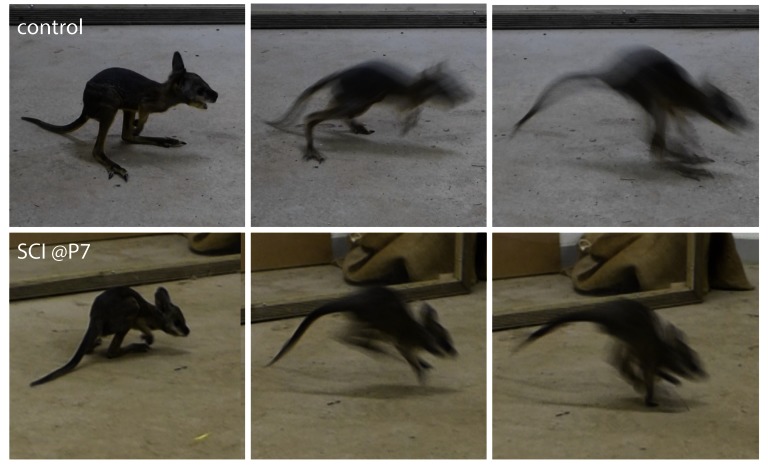
Hopping test in pouch young tammars at pouch exit. At about P180–200 pouch young periodically start leaving the pouch. Control and P7 group transected (SCI@P7–P13) animals were placed on a hard surface in the Animal House and allowed to hop. Videos of their movements can be found in the ( Supplementary Material S1 and Supplementary Material S2). There was no obvious difference in the way young from either group moved, all were very active and equally fast. Their use of the tail for support was also similar (n=3).

**Figure 8.**
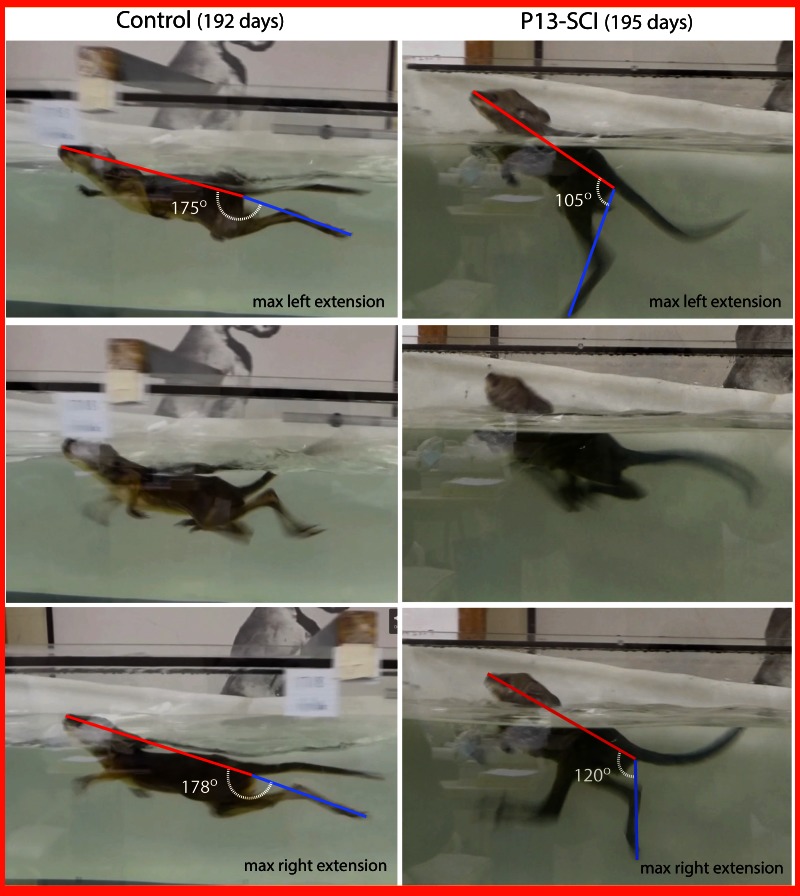
Swimming test of pouch young tammars at pouch exit. P190–200 pouch young were placed in a swimming tank (see Methods) and their swimming recorded (see Supplementary video S3 and Supplementary video S4). Control animals used alternating hind-limbs kicks with full retraction and posterior extension of the hind legs in kicking movements. Transected animals (SCI@P13) were also able to swim, but their tails were less flexible and their hind-limbs extended more vertically relative to the body during the kicking stroke (n=3). To highlight these differences in body position while swimming, still images were captured from the videos where there was full extension of each hind-limb. The angle made between the body axis and the extended hind-limb axis was measured by drawing a line between the tip of the nose and the ventral base of the tail (body axis, red line) and between the ventral base of the tail and the tip of the toes on the extended hind limb (hind-limb axis, blue line). In control animals, this angle was close to a horizontal line, but in spinally injured animals the angle was more acute.

All surviving PY were retested at P190 for hopping and swimming ability. Still images from video footage of hopping are included in Figure 7 and swimming in Figure 8. There was no visible difference in the ability of animals transected at P7−15 and controls to hop over ground, all were able to stand upright on their hind legs using their tail for support (see Supplementary Video S1 and Supplementary Video S2). However, differences were noted during the swimming test between the two groups. Control animals (see Supplementary Video S3) used alternating strong rhythmic hind-limb kicks extending out behind the body coupled with snake like sideways movements of the tail to propel themselves forwards, while maintaining a fairly horizontal body orientation. Measurements of the angle between the hind-limb axis at full extension (line drawn between the tip of the hind-leg and ventral base of the tail) and the axis of the body (line drawn between the tip of the nose and ventral base of the tail) in control animals were close to a straight line (175−180°; Figure 8). These animals consistently used their forelimbs in coordination with their hind-limbs. In contrast, animals transected at P7–15 (n=3) also used their hind-limbs in an alternating and coordinated fashion; however, the hind-limb kicks were in a more vertical plane, lacking the normal posterior extension, which resulted in reduced ability to propel forwards. They also used their tail, but in an upwards/downwards plane rather than horizontal as controls did. They have also shown less coordination of forelimbs with hind-limbs (see Supplementary Video S4) and maintained a much more vertical hind-limb angle at full extension compared to controls when swimming (105−140°; Figure 8). We have observed some variation in swimming performance between injured animals. All were able to swim with rhythmical hind-limb kicks, but 2 out of 3 had difficulty propelling themselves forward in a straight direction, instead moving in a circle. Figure 8 (and Supplementary Video S4) shows the best performing of the spinal injured animals.

These results demonstrate that if a spinal transection is performed during the first 3 weeks of life of the tammars, their spinal cords not only repair themselves morphologically, but this repair also translates into functional recovery (see Discussion).

Archived video and histological data. Click here for additional data file. Copyright: © 2017 Saunders NR et al. 2017 Data associated with the article are available under the terms of the Creative Commons Zero "No rights reserved" data waiver (CC0 1.0 Public domain dedication).

## Discussion

In this study, we aimed to establish in the tammar periods of spinal cord development when, following a complete mid thoracic transection, supraspinal axons are able to bridge the injury site and when this ability is lost in development. Similar periods in development of another marsupial, *Monodelphis domestica* have been described and the terms “permissive” and “non-permissive” stages of cord maturation were proposed ( Noor *et al.*, 2013), as previously suggested for chick embryo spinal cord ( Keirstead *et al.*, 1992) (see below). Results obtained here confirm that during tammar development there is indeed a permissive period of spinal cord maturation (first 3 weeks of life) followed by a non-permissive period (from P40 onwards).

### Use of marsupials as experimental models

Marsupials are unique experimental animal models, especially for developmental studies, for a number of important reasons. All marsupials, including tammars, are born at a very immature stage of early development and most brain development occurs *ex utero* ( Dziegielewska *et al.*, 1989; Riese, 1948; Renfree *et al*., 1982; Reynolds *et al.*, 1985; ). While there are variations in details of anatomical development between different marsupials, at birth in most cases their stage of central nervous system (CNS) development corresponds to approximately that of a rat at embryonic day 14 and a human at about six weeks ( Comans *et al.*, 1987; Nicholls *et al.*, 1990; Reynolds *et al.*, 1985; Saunders *et al.*, 1989). This means that experiments performed previously in eutherians *in utero* can be performed *ex utero* in marsupials, automatically eliminating the many risks and complications involved in performing ** surgery on pregnant females ( Saunders *et al.*, 1989).

Previous studies have used the marsupial South American opossum ( *Monodelphis domestica*) and North American opossum ( *Didelphis virginiana*) to demonstrate the effects of spinal cord injuries at different stages of development ( Fry & Saunders, 2000; Martin & Xu, 1988; Saunders *et al.*, 1989; Saunders *et al.*, 1998; Wang *et al.*, 1996; Wang *et al.*, 1998 *a*; Wheaton *et al.*, 2011; Wheaton *et al.*, 2013; Wheaton *et al.*, 2015; Xu & Martin, 1992). It has been shown that when pups were injured early in development at post-natal day P7, regrowth of supraspinal neuronal axons across the lesion site was demonstrated (termed the “permissive” stage, as previously suggested for the period when functional repair occurred in chick embryonic spinal cord following SCI; Keirstead *et al.*, 1992). However, when injured at a later stage of development, at P28, no regeneration of axons was observed (“non-permissive” stage). Martin and colleagues referred to “the critical period” for regenerative growth in postnatal *Didelphis* and a subsequent period when the local environment at the site of injury was “non-permissive” (e.g. Terman *et al.*, 1999; Wang *et al.*, 1998 *a*). Several attempts have been made to identify cellular and molecular changes that could explain this shift in the ability of axons to bridge the site of injury ( Noor *et al.*, 2011; Saunders *et al.*, 1998; Saunders *et al.*, 2014; Terman *et al.*, 1999; Wang *et al.*, 1998 *b*; Wheaton *et al.*, 2015) and some suggestions that it could be related to the onset of myelination were also put forward ( Bandtlow, 2003; Saunders *et al.*, 2014; Wheaton *et al.*, 2015).

In the present study we have sought to define these “permissive” and “non-permissive” stages in the tammar. Our results demonstrate that the period of development that is permissive to spinal cord regeneration extends up to P20−25 and animals older than P40 at the time of injury show no regeneration. Cord development of the tammars appears to be similar to that of the *Monodelphis domestica* ( Nicholls *et al.*, 1990) at similar ages *.* By P1 the tammar spinal cord shows a deep central canal surrounded by a proliferating neuroepithelium. The dorsal horn contains advanced neurons at cervical and brachial levels; however, fewer developed neurons are present in the lumbosacral cord ( Harrison & Porter, 1992). By approximately P17, the spinal cord has reached a mature form with a small central canal, fully formed dorsal horns and distinct fasciculi gracilis and cuneatus in the dorsal column ( Comans *et al.*, 1987) and at P20 the corticospinal tracts begin to form ( Ashwell, 2010). The switch to a non-permissive phase of development appears to occur soon after this stage.

### The tammar wallaby as a model for bipedal locomotion

Following SCI, patients face many problems in trying to regain some degree of function and independence. Being able to use legs for voluntary movement is only one part of this process. The ability to successfully walk would also require balance. This is critically dependent on the *stability of the trunk*. This is one fundamental limitation for the use of quadrupeds as models for studying recovery of function after spinal trauma that could complicate a treatment’s eventual transfer to humans. Tammars, like humans, require trunk stability and are a truly *unique animal model,* in which it is possible to answer many questions not answerable in quadrupeds (e.g., the commonly used rodent models).

For many bipedal animals (including humans), to implement successful locomotion one leg must support the body in stance, while the other limb lifts off the ground and swings through to produce forward movement. To achieve this there must be activity in the descending motor pathways, to innervate and coordinate muscle contraction of the limbs, as well as activity of the trunk musculature to trigger compensatory postural movements, preventing the body from losing balance. Quadrupeds are able to use their fore limbs to compensate for balance deficits in the hind quarters, whereas bipeds cannot, at least not while maintaining unsupported bipedal locomotion. Although some rat studies have used a harness for body weight support and obliged spinal rats to walk on a treadmill using only hind limbs (e.g. van den Brand *et al.*, 2012), this approach merely provides external support for the body and does not take into account autonomous trunk stability during bipedal gait. In one of the few studies of compensatory trunk control in quadrupeds, Giszter *et al.* (2007) showed that spinal injured rats were able to modify command of the trunk musculature, unloading the hind-limbs in order to compensate for their aberrant stepping ( Giszter *et al.*, 2007). Crucially though, these modifications served primarily to shift weight support to the forelimbs ( Giszter *et al.*, 2008), an option that bipeds do not have.

The bipedal hopping locomotion of macropods, such as tammars, might appear significantly different from bipedal locomotion in humans; however, the underlying spinal cord circuits are likely to be similar and certainly not exclusive to macropods. There are reports that now-extinct species of macropod used bipedal striding gait rather than hopping and tree-kangaroos have been observed to walk bipedally along branches ( Janis *et al.*, 2014); larger species of macropod, but not the smaller tammar, use a low speed pentapedal gait when foraging ( Dawson *et al.*, 2015). Hopping is energetically efficient compared with other forms of locomotion and perhaps evolved in Australia in response to the large distance and limited food supply in an arid environment ( Baudinette *et al.*, 1992). Of particular relevance to the present study are the publications of Kiehn (e.g. Kiehn, 2011), who has described in detail the locomotor networks responsible for rhythmic coordinated limb movements in neonatal animals. This suggests that the underlying circuitry and nervous system development involved is similar.

### The central pattern generator and the importance of the swimming test

When behavioural analysis of *Monodelphis* with complete spinal transections at either P7 (permissive) or P28 (non-permissive) was performed, both experimental groups maintained the ability to walk over-ground using coordinated fore and hind limb movements ( Wheaton *et al.*, 2011; Wheaton *et al.*, 2013). This was in spite of the fact that animals injured at P28 did not have any supraspinal innervation below the thoracic transection ( Wheaton *et al.*, 2011). However, when these animals were subjected to a swim test, *Monodelphis* injured at P28 were observed to only use their forearms to navigate through the water, in contrast to animals injured at P7 that could swim using all four limbs ( Wheaton *et al.*, 2011). This was evidence that the over-ground locomotion observed in the P28-injured opossums (those without supraspinal innervation to the hind limbs) was dependent upon reflex input and without it (in the case of the swim test) the limbs were receiving insufficient signals to move.

There have been many studies in quadrupedal animals leading to the idea of a central pattern generator in the lumbar spinal cord, which is a local circuit that has the ability to generate rhythmic movement of the hind limbs when activated by peripheral sensory input ( Grillner *et al.*, 2008; Kiehn, 2011; Pearson, 2000; Rossignol & Frigon, 2011; ). This makes the swim test a good test to detect the effects of locomotion in the absence of this reflex input, and a good measure of supraspinal control ( Saunders *et al.*, 1998; Smith *et al.*, 2006; Magnuson *et al.*, 2009; Wheaton *et al.*, 2011; Wheaton *et al.*, 2013).

In the present study, we have shown that control tammar young are able to swim using hind limbs, as well as forelimbs and the tail from about P190. Young with SCI performed at P7–20 were equally able to swim using hind limbs and the tail, but in a pattern different from un-operated controls (see Figure 8 and Supplementary Video S3 and Supplementary Video S4). Thus this swimming test confirms the tracing studies showing retrogradely labeled axons spanning the site of injury in P7 and P20 groups of experimental animals ( Figure 4 and Figure 5), and suggests the re-establishment of functional supraspinal control.

While there has been limited research into the rhythm generators in the spinal cord that contribute to bipedal walking ( Courtine *et al.*, 2009; Rossignol & Frigon, 2011; van den Brand *et al.*, 2012), there have been attempts to translate these findings into humans with SCI. In these trials, patients were supported in a sling while walking on a treadmill in order to improve their weight-bearing locomotion; however, over time no improvements were observed ( Dobkin *et al.*, 2006). This may be a consequence of the loss of core strength in humans due to the loss of innervation of the core muscles of the trunk. Quadrupeds gain stability through their fore limbs and thus rely less on the core strength through their trunk. Therefore supraspinal input may not play such an important role in quadruped locomotion after injury ( Wheaton *et al.*, 2011). Similarly, the Australian brushtail possums possess propriospinal mechanisms that control the release of hind limb grip when the forepaws are activated during climbing ( Ashwell, 2010), a mechanism which does not benefit bipeds. This is why it is important to establish a bipedal animal model of spinal cord injury.

### Comparison of the SCI experimental design in tammars and in *Monodelphis*

The fluorescent labeling undertaken here in the tammar study showed definitive evidence of regrowth of supraspinal axons through the injury site ( Figure 4) in animals injured in the first three weeks of life, but no regrowth after this age. Previous work employing labeling methods has identified similar regenerative periods in *Monodelphis* (regeneration up to P14; Fry *et al.*, 2003; Lane *et al.*, 2007) and *Didelphis* (regeneration up to P24, Wang *et al.*, 1998 *a*). The main difference between the studies of Fry *et al.* ( Fry *et al.*, 2003) in *Monodelphis* and the present one in tammars was that the injection protocol was designed to study true regeneration as opposed to re-growth of axons after transection that would occur as part of normal development. For that purpose, injection of the first marker was done 3 days *before* the transection to label axons that were subsequently cut, while in the present study the first marker was injected *immediately* after the transection. This difference in the design stems from the fact that *Monodelphis* are easier experimental animals to maintain as a colony: they breed all year around, have multiple young and are the size of a small rat ( Saunders *et al.*, 1989), while tammars breed naturally once a year and usually have one PY ( Tyndale-Biscoe & Janssens, 1988). Therefore, the completeness of the transection was deemed to be more important to establish in the present study as the objective was to determine the permissive versus non-permissive ages in a new species.

## Limitations of the study

This study has two main limitations: (i) The small numbers of animals that were available and (ii) the problems we encountered in achieving survival of older (P40 and P60) operated animals.

Over many decades, studies on marsupial biology, particularly reproductive and developmental biology, were internationally recognised strengths of Australian science, much of it carried out in government research institutes, such as the former CSIRO Wildlife Research in Canberra. However, with the increasing emphasis on utilitarian research over the past 3−4 decades ( Saunders, 1989) the facilities for such fundamental research have declined. We were fortunate to obtain access to the remnants of a once large colony of tammars at the CSIRO in Canberra. In addition to the small numbers of animals available from the colony, tammars, like many Australian marsupial species, generally produce only one young per year and the lengthy period in the pouch makes for long experiments. This is in contrast to the South American opossums, which will breed all the year round in captivity, have multiple young and reach maturity in a shorter time. An experimental advantage of the tammar over the South American opossum is that the tammars look after their young in a pouch, whereas the opossum is pouchless. This means that in effect the tammar mother provides us with a natural incubator for the postoperative young. This was effective for the younger (P7) operated animals, where the main reason for discarding animals from the analysis was evidence that the spinal transections were incomplete. For the older operated animals, as outlined in the Results section, young were lost before the time they would normally begin to exit the pouch. It was unclear whether these losses were due to the mother recognizing that the pouch young were abnormal or because if having left the pouch the young were sufficiently disabled as to be unable to return. Whatever the explanation, the result is important because it suggests that bipedal tammars are less able to cope with a complete spinal lesion after the period when regeneration naturally occurs, compared with the quadrupedal *Monodelphis*, which exhibited weight bearing quadrupedal locomotion in the absence of any axon growth across the lesion ( Wheaton *et al.*, 2011; Wheaton *et al.*, 2013). This confirms the conclusion of Côté *et al.* (2017) that peripheral sensory inputs may be insufficient to drive locomotion in bipedal animals. In future studies it should be possible to use artificial incubators and feeding systems to maintain these older SCI tammars to later stages of development in order to confirm that their locomotor activity is indeed limited by the loss of sensory input via forelimb stretch, as appears to occur in *Monodelphis* with complete SCIs made at 4 weeks of age ( Wheaton *et al.*, 2011; Wheaton *et al.*, 2013).

There is also the question to what extent bipedal locomotion in the tammar can accurately equate to bipedal locomotion patterns in humans. Important evidence suggesting that the spinal circuits involved in bipedal locomotion in tammars are likely to be similar to those in humans comes from the observation that tammars swim using alternating movements of their hind limbs. The evolutionary reasons for why tammars and kangaroos in general have adopted a bipedal hopping gait are discussed above, but their swimming movements suggest similarity of the spinal circuits in tammars and humans. The nature of the spinal rhythm generators in the tammar is not known, but will be an important topic for future investigations.

## Conclusions

The experiments reported here provide further evidence in another species (tammar wallaby) for an early period of CNS development that is “permissive” for axon growth and functional recovery, in contrast to a later stage when axon growth does not occur (“non-permissive”). An important difference in this study of a species with bipedal gait compared to quadrupedal opossums is the poor locomotor recovery in the tammar when the spinal cord is injured during the “non-permissive” period. We propose that this difference may be due to sensory feedback from the limbs being much less effective in promoting locomotion in bipedal animals, as suggested by Côté *et al.* (2017) for humans. This not only may contribute to explaining the lack of translation to human patients of apparently effective therapies based on experiments in quadrupedal rats, it also provides a potential solution, namely to develop tammars or other macropods as animal models for testing potential therapies for human patients.

## Data availability

The data referenced by this article are under copyright with the following copyright statement: Copyright: © 2017 Saunders NR et al.

Data associated with the article are available under the terms of the Creative Commons Zero "No rights reserved" data waiver (CC0 1.0 Public domain dedication). 

**Dataset 1: Archived video and histological data.** http://dx.doi.org/10.5256/f1000research.11712.d164069 ( Saunders *et al.*, 2017).

Video files:

- ***PY2835 Control Hopping.mov,*** uninjured control tammar, video recorded at age P191;
- ***PY2988 Control Hopping.mov,*** uninjured control tammar, video recorded at age P196;
- ***PY2988 Control swimming.mov,*** uninjured control tammar, video recorded at age P196;
- ***PY2846 Control swimming.mov,*** uninjured control tammar, video recorded at age P199;
- ***PY2836 SCI@P12 Hopping.mov,*** spinal transection at P12, video recorded at age P195;
- ***PY2839 SCI@P7 Hopping.mov,*** spinal transection at P7, video recorded at age P195;
- ***PY2836 SCI@P12 swimming.mov,*** spinal transection at P12, video recorded at age P191.

Note: video recordings were made for animals that could swim when placed into the tank. Animals injured at later ages (P40-P60) were not able to swim.

Histology files:

- ***Control SC sections.pdf,*** serial sections of a control tammar spinal cord, H&E stained;
- ***H&E serial sections.pdf,*** cross-sectional area measurements of H&E stained serial sections of tammar spinal cords injured at P7, P40 and P60. Cords collected at P195-198. Includes H&E stained serial sections of P60 injured spinal cord.
- ***P7-60 injury centre sections.pdf,*** H&E stained sections from the lesion centre of tammar spinal cords injured between P7 and P60 and collected at P195-198.

Retrograde tracing and cell counts:

***Saunders et al Raw data files.pdf***

- Head length and body weight measurements of the tammar young
- Position and number of green and red retrograde labelled cell bodies in the brainstem regions of the tammar cords.
- Images of the injection sites for the retrograde tracers

***Brainstem cell counts data.xls***

- Head length and body weight measurements of the tammar young
- Position and number of green and red retrograde labelled cell bodies in the brainstem regions of the tammar cords.

## Supplementary material

**Supplementary Videos S1 and S2: Over ground locomotion of control (S1) and operated (S2) tammars.** At about P180−200, pouch young periodically start leaving the pouch. Control and P7−13 group transected animals were placed on a hard surface in the Animal House and allowed to hop. Note that there were no obvious differences in the way young from either group moved, all were active, propelled themselves equally fast and were able to sit upright on their hind-legs with front paws by their sides. Their use of the tail for support when standing was also similar. Three animals in each group were analyzed and four independent, blinded observers were asked to identify any differences. None were observed.

.

**Supplementary Videos S3 and S4: Control (S3) and operated (S4) tammars swimming.** After completing the over ground locomotion test ( Figure 7; Supplementary Video S1 and Supplementary Video S2), pouch young were placed in a swimming tank (27°C) and their swimming recorded. Control animals used alternating hind-limbs kicks with full retraction and extension of legs in kicking movements. Transected animals were also able to swim, but their tails were less flexible and their hind-limbs showed less posterior extension in the kicking stroke. In addition their front paws were less coordinated with hind-legs than in control young. N=3 per group.
