# Supplementary material for: A bipedal mammalian model for spinal cord injury research: The tammar wallaby
Source: F1000Res. 2017 Jun 15;6:921. [Version 1] doi: 10.12688/f1000research.11712.1 (PMC5497825; doi:10.12688/f1000research.11712.1)
Supplement: Archived video and histological data. [file f1000research-6-12654-s0000.tgz › 00cb5ab8-8bc4-420a-83a6-f865b6698448_Dataset_1.zip!/Dataset 1/Control SC sections.pdf]

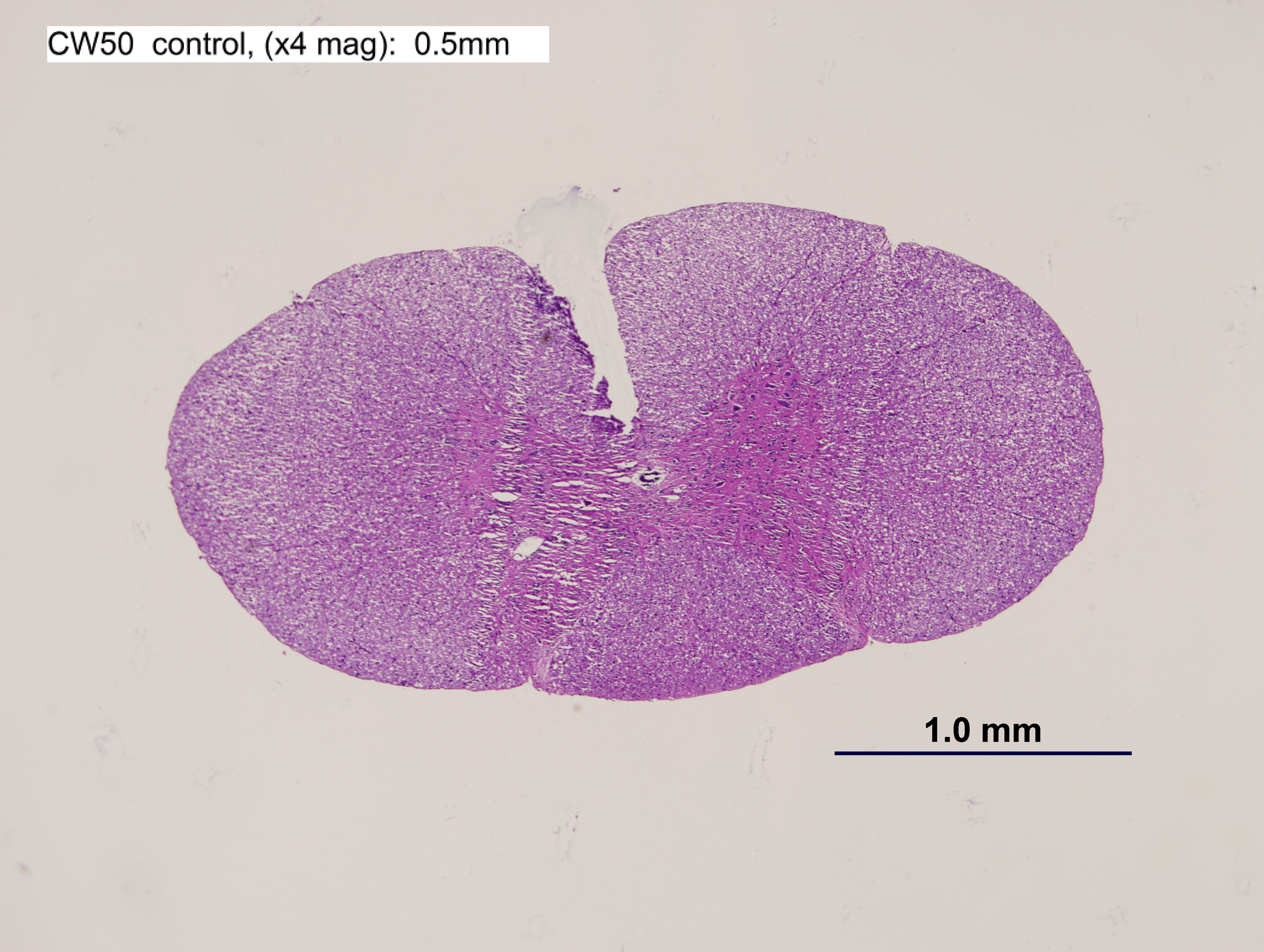
CW50 control, (x4 mag): 0.5mm
1.0 mm

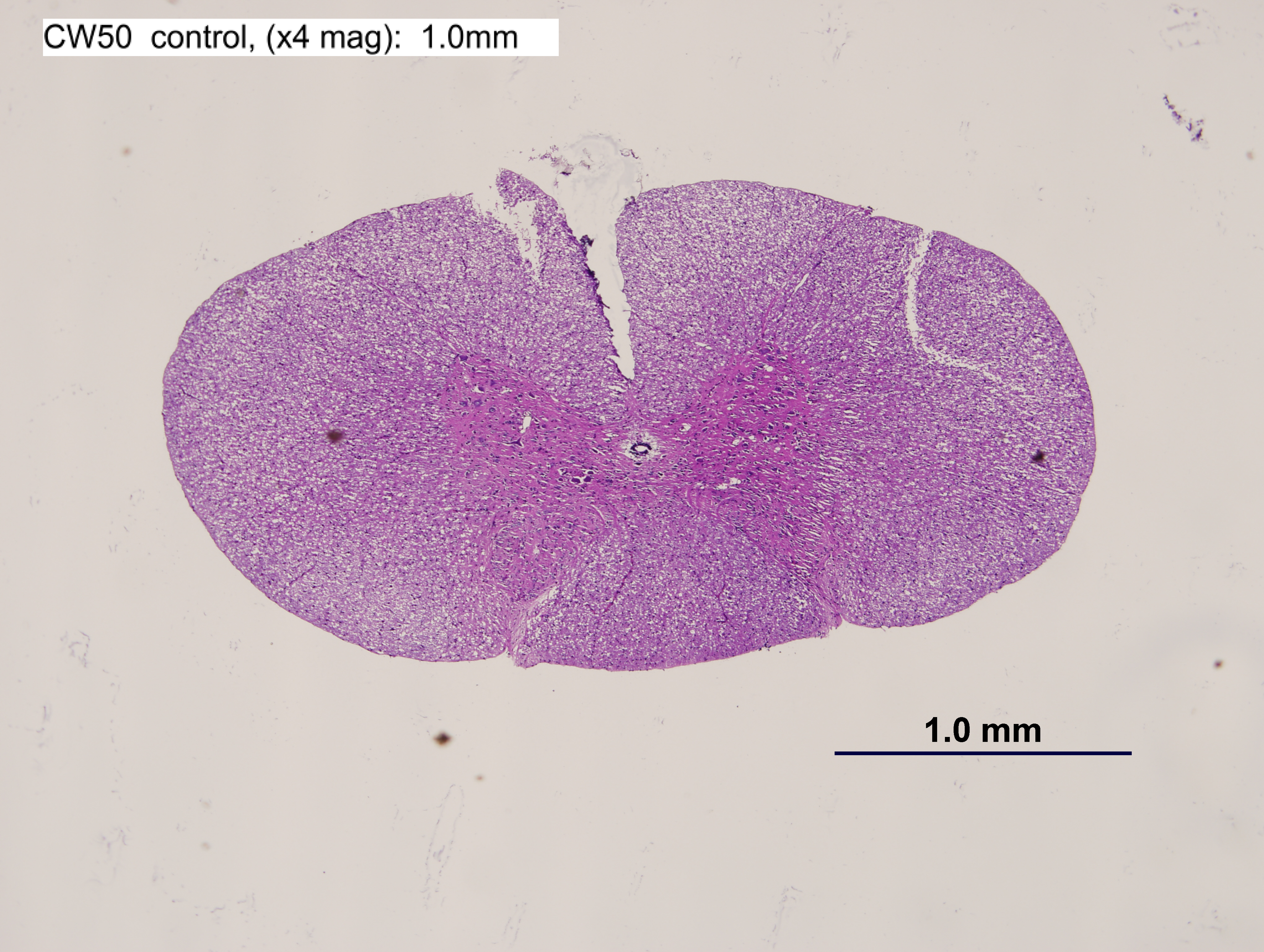
CW50 control, (x4 mag): 1.0mm
1.0 mm

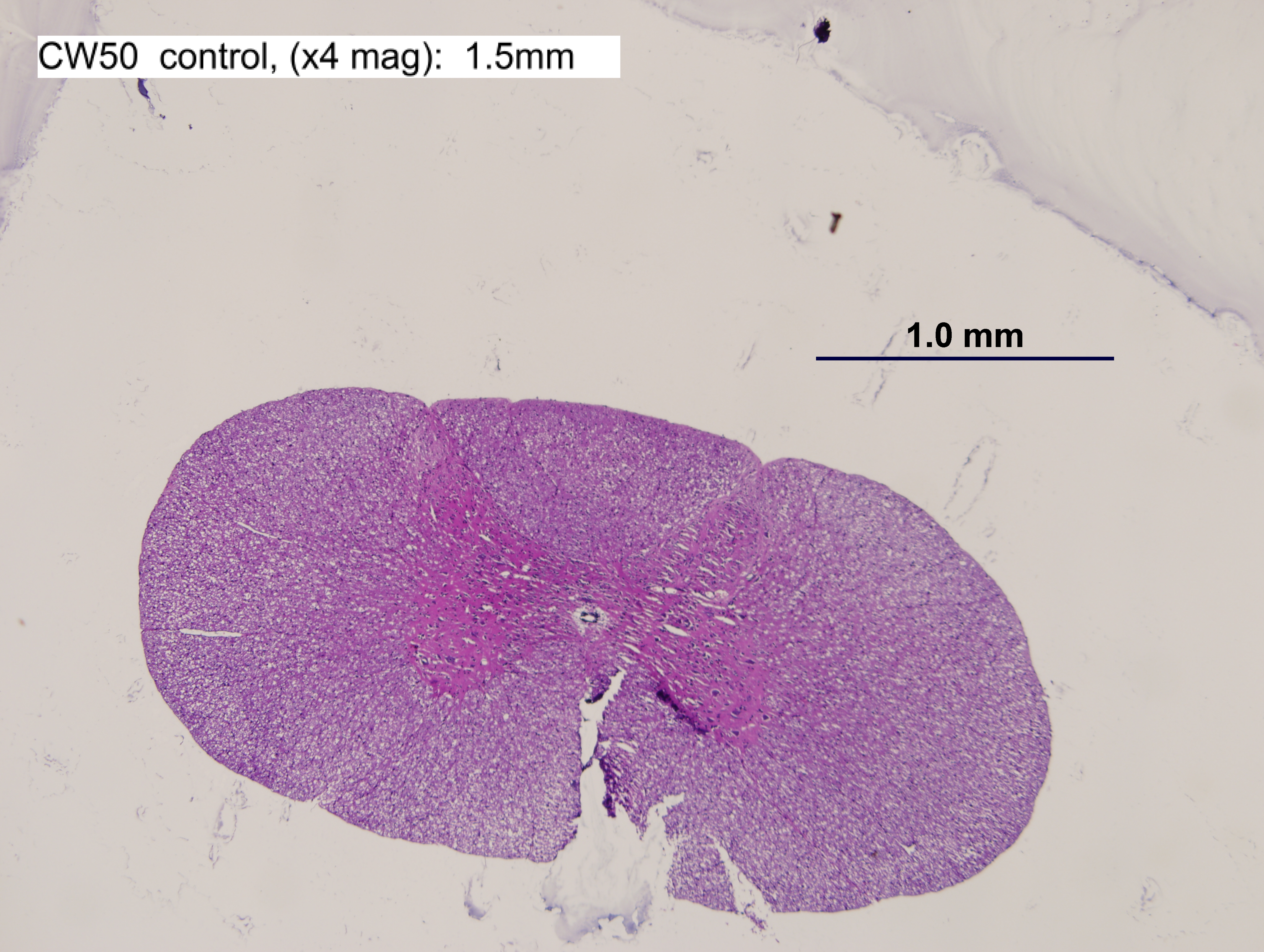
CW50 control, (x4 mag): 1.5mm
1.0 mm

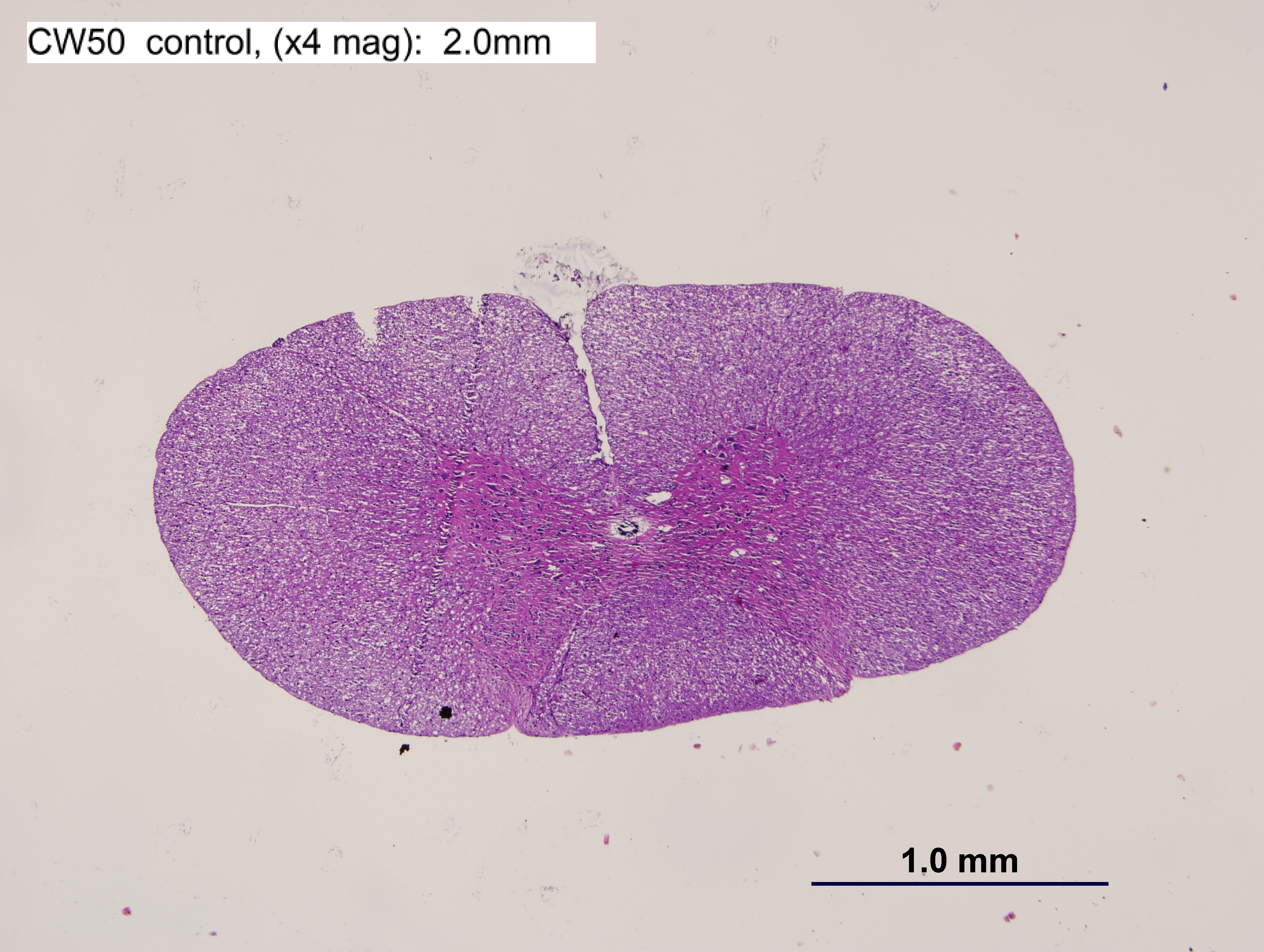
CW50 control, (x4 mag): 2.0mm
1.0 mm

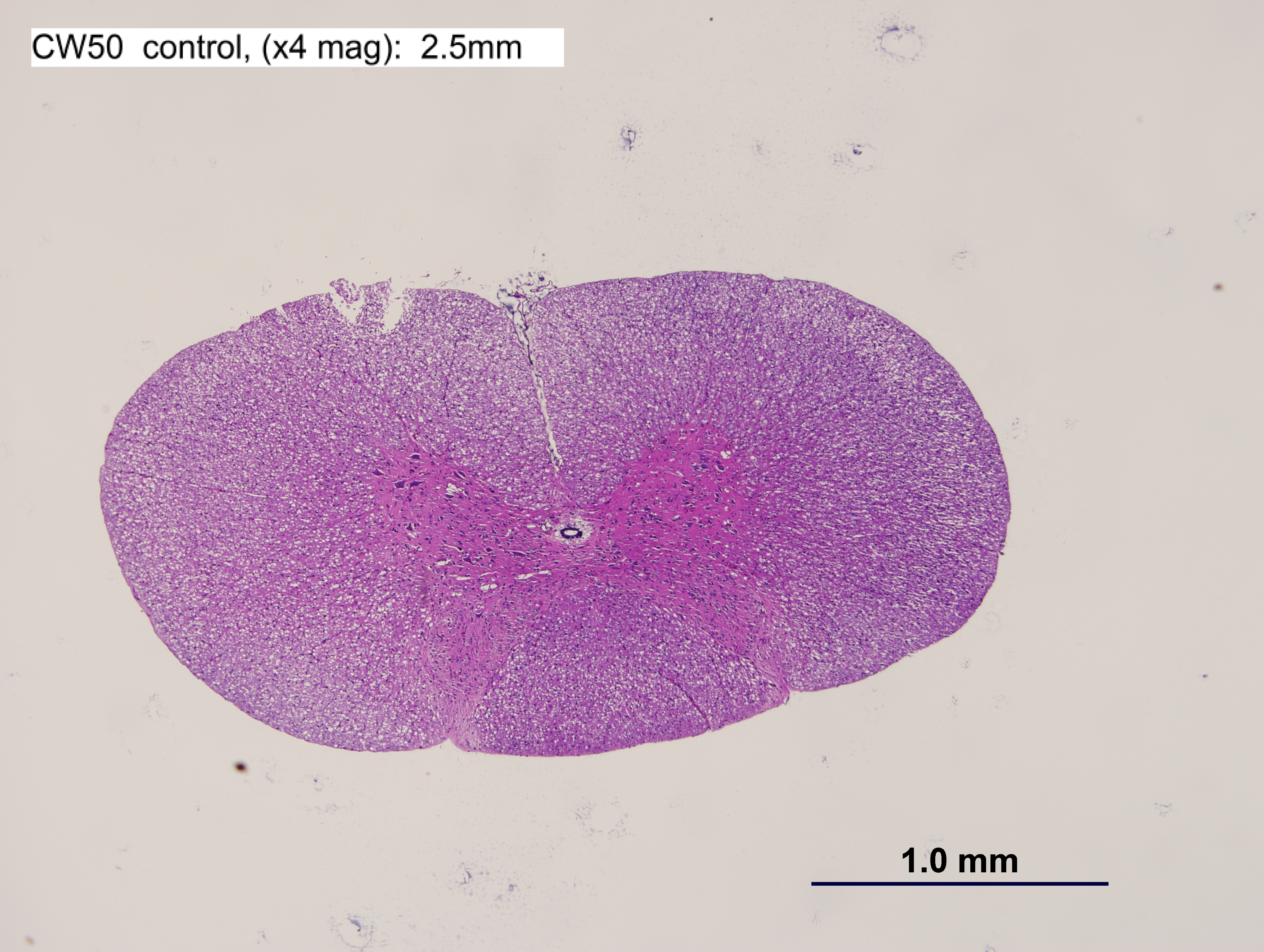
CW50 control, (x4 mag): 2.5mm
1.0 mm
