# Supplementary material for: A bipedal mammalian model for spinal cord injury research: The tammar wallaby
Source: F1000Res. 2017 Jun 15;6:921. [Version 1] doi: 10.12688/f1000research.11712.1 (PMC5497825; doi:10.12688/f1000research.11712.1)
Supplement: Archived video and histological data. [file f1000research-6-12654-s0000.tgz › 00cb5ab8-8bc4-420a-83a6-f865b6698448_Dataset_1.zip!/Dataset 1/H&E serial sections.pdf]

Area measurements (mm^2) from serial
sections of Tammar spinal cords (H&E) relative to the injury centre (0mm).

Negative numbers are rostral, positive numbers are caudal.

P ages are the age at the time of spinal injury.

Distance (mm)	P40	P60	P7
-4.50			3.484
-4.00	1.203		3.459
-3.50	1.105		3.289
-3.00	1.028	1.340	3.048
-2.50	0.718	1.007	3.060
-2.00	0.483	0.618	2.698
-1.50	0.263	0.224	2.598
-1.00	0.123	0.086	2.387
-0.50	0.030	0.032	2.262
0.00	0.034	0.030	2.299
0.50	0.067	0.008	2.308
1.00	0.175	0.031	2.351
1.50	0.328	0.286	2.566
2.00	0.571	1.140	2.901
2.50		1.545	3.100
3.00		1.651	3.332
3.50		1.684	3.374
4.00			3.501

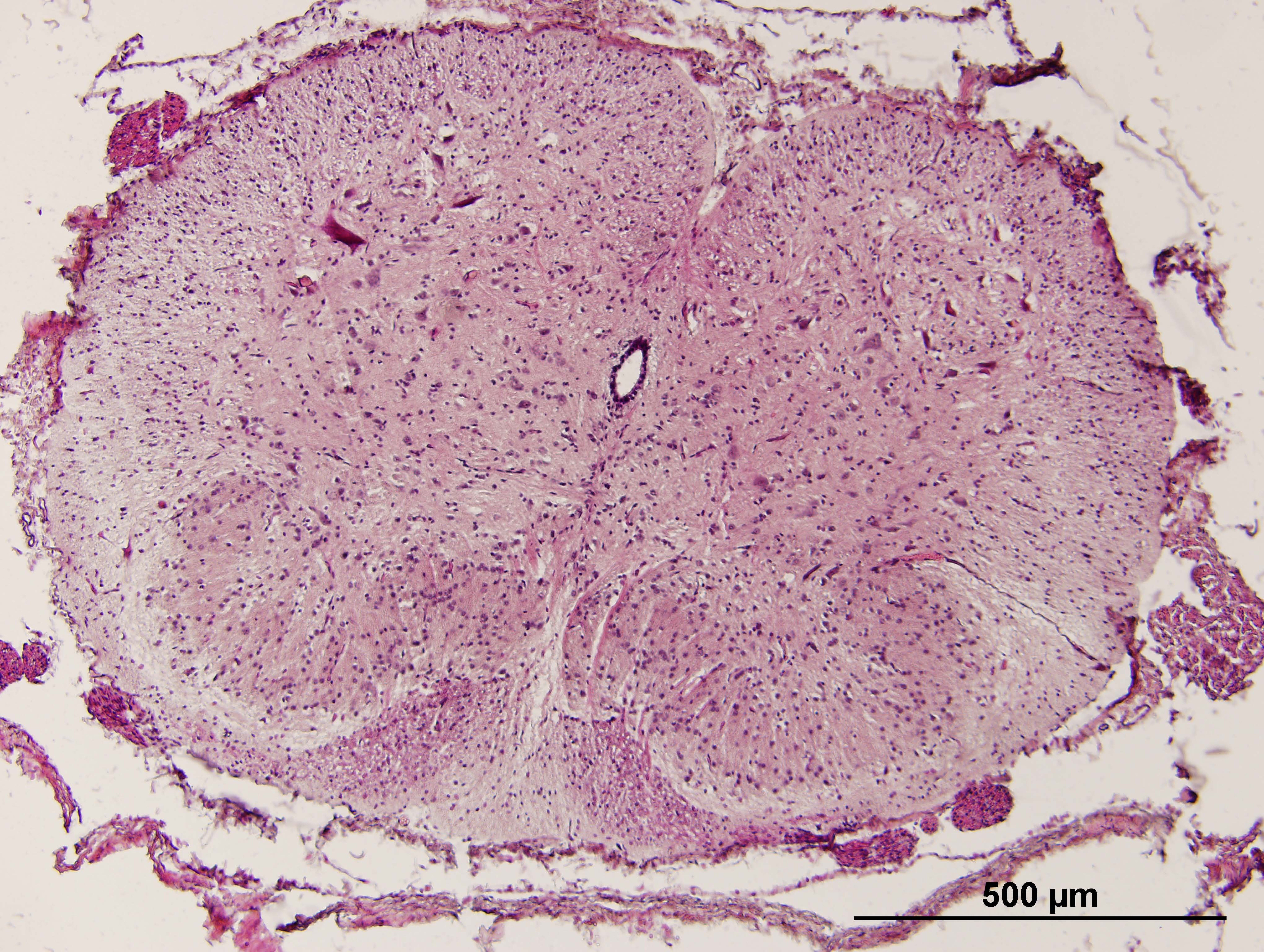
500 µm

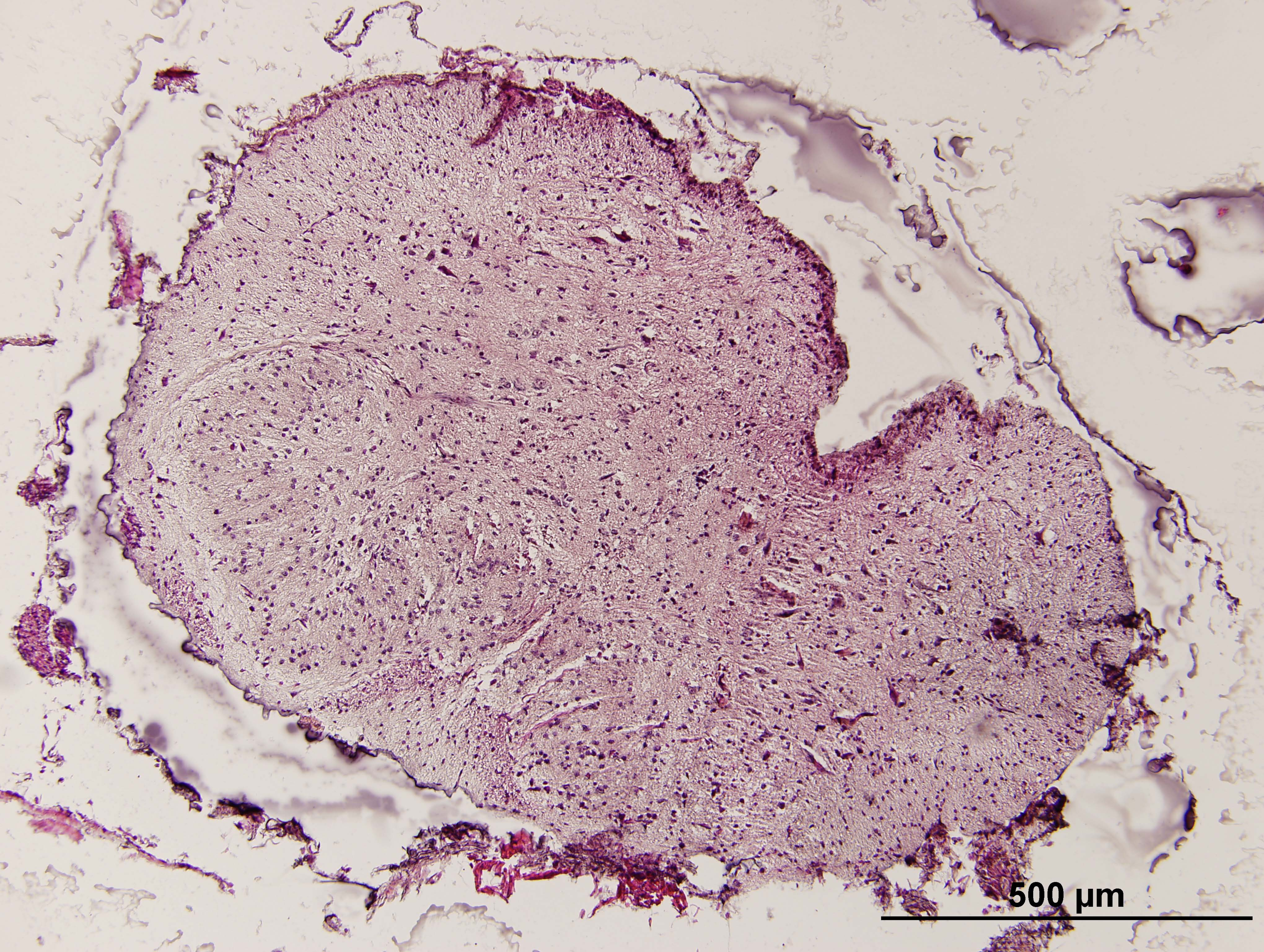
500 µm

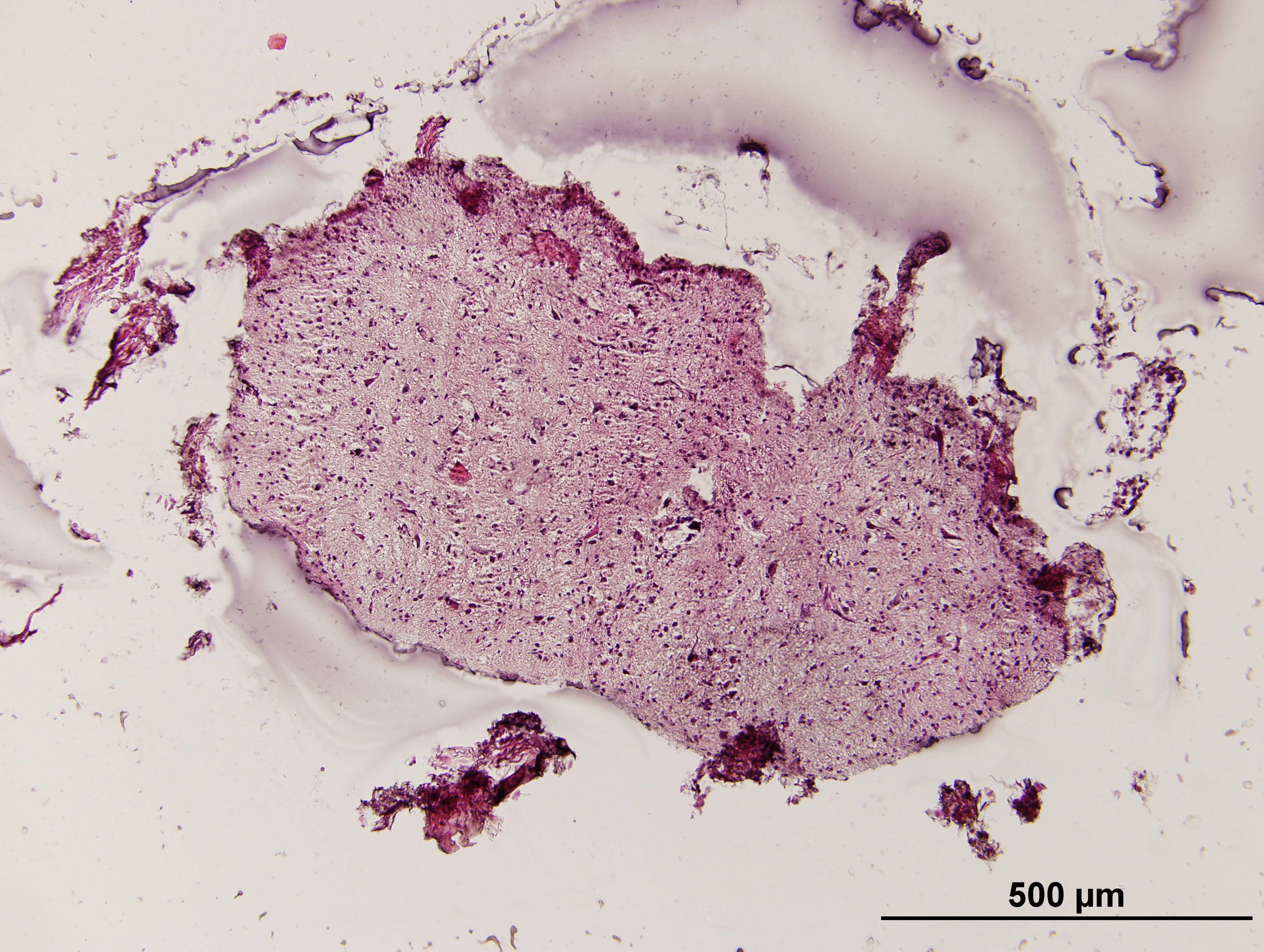
500 µm

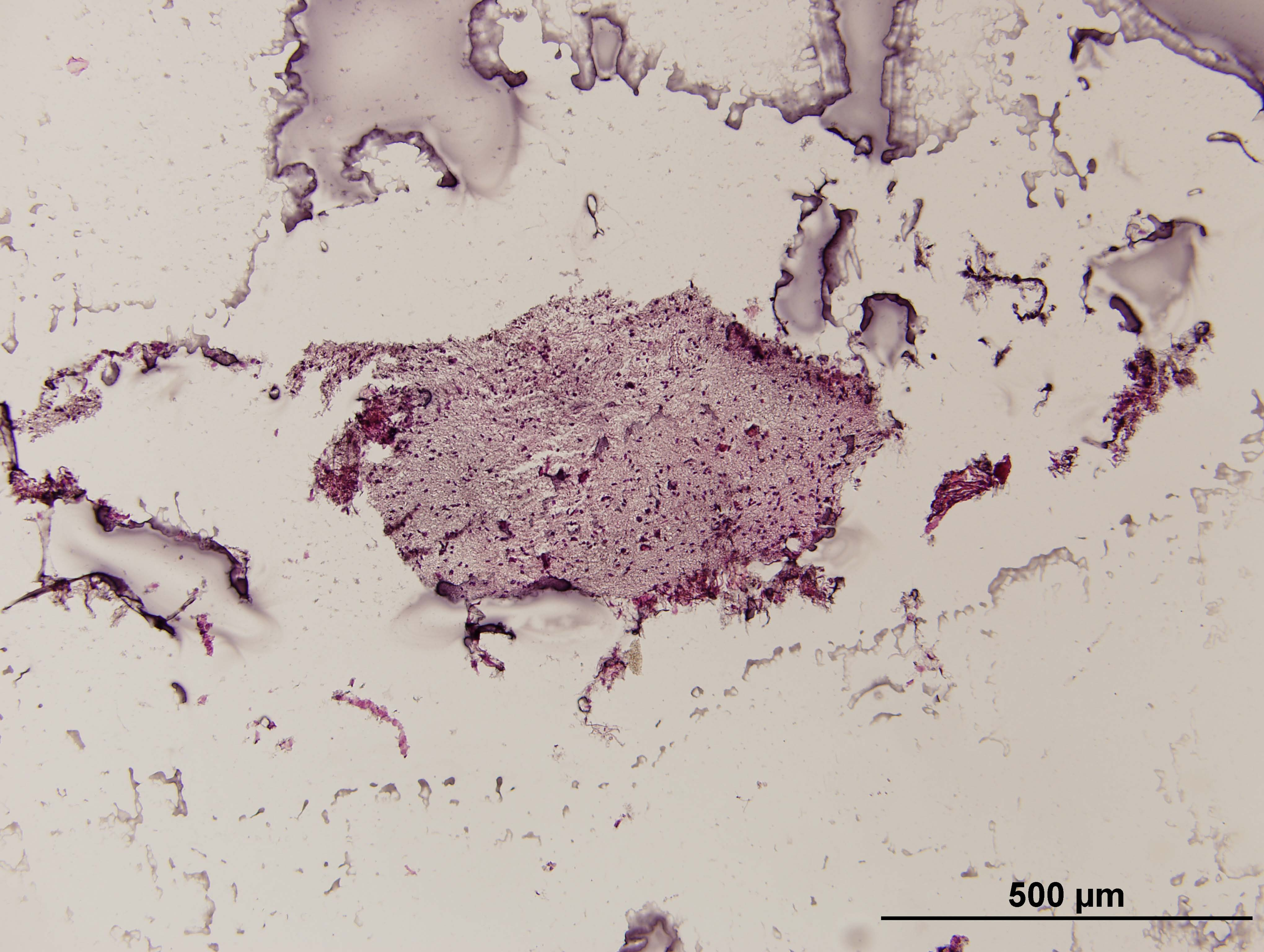
500 µm

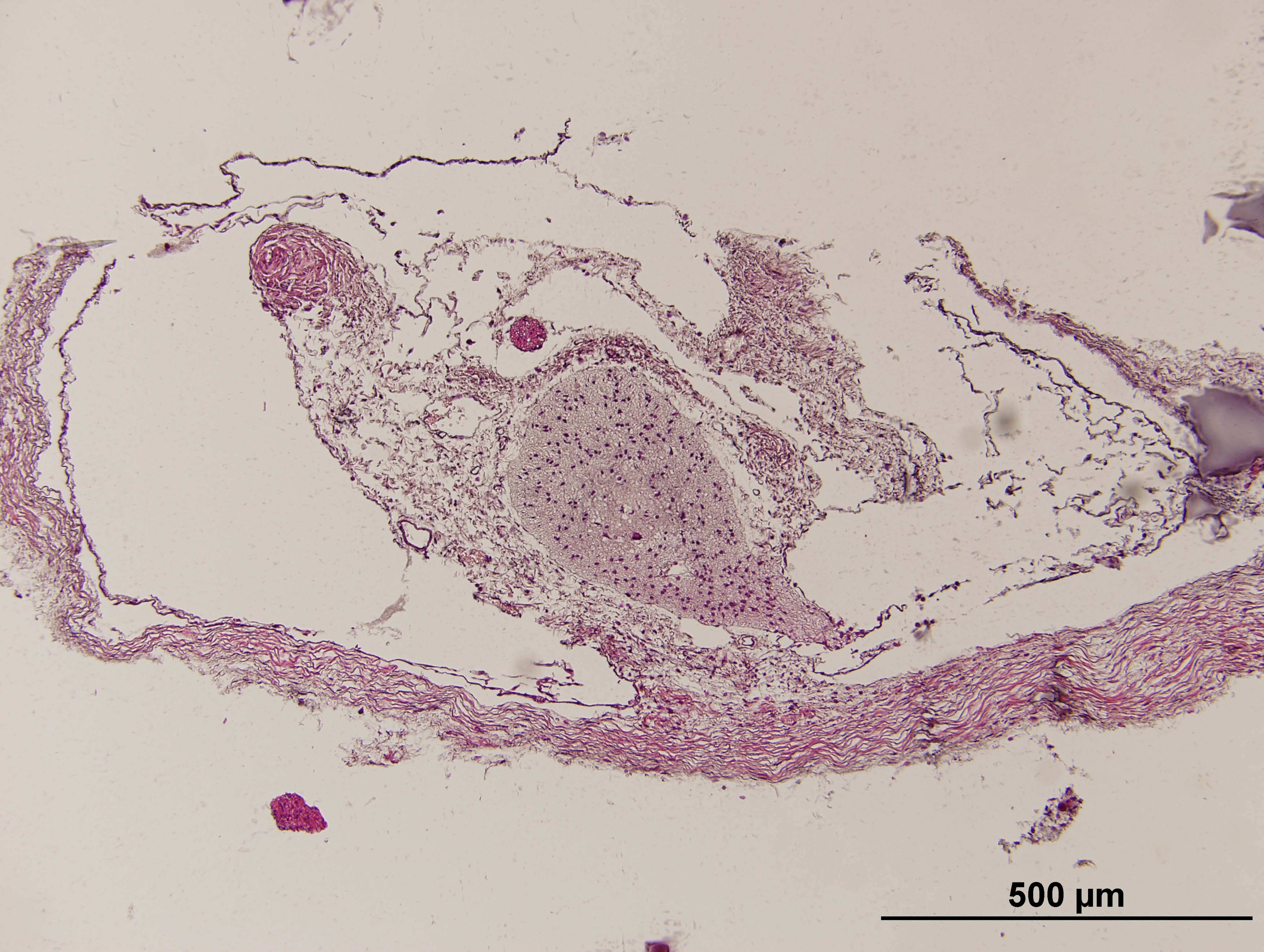
500 µm

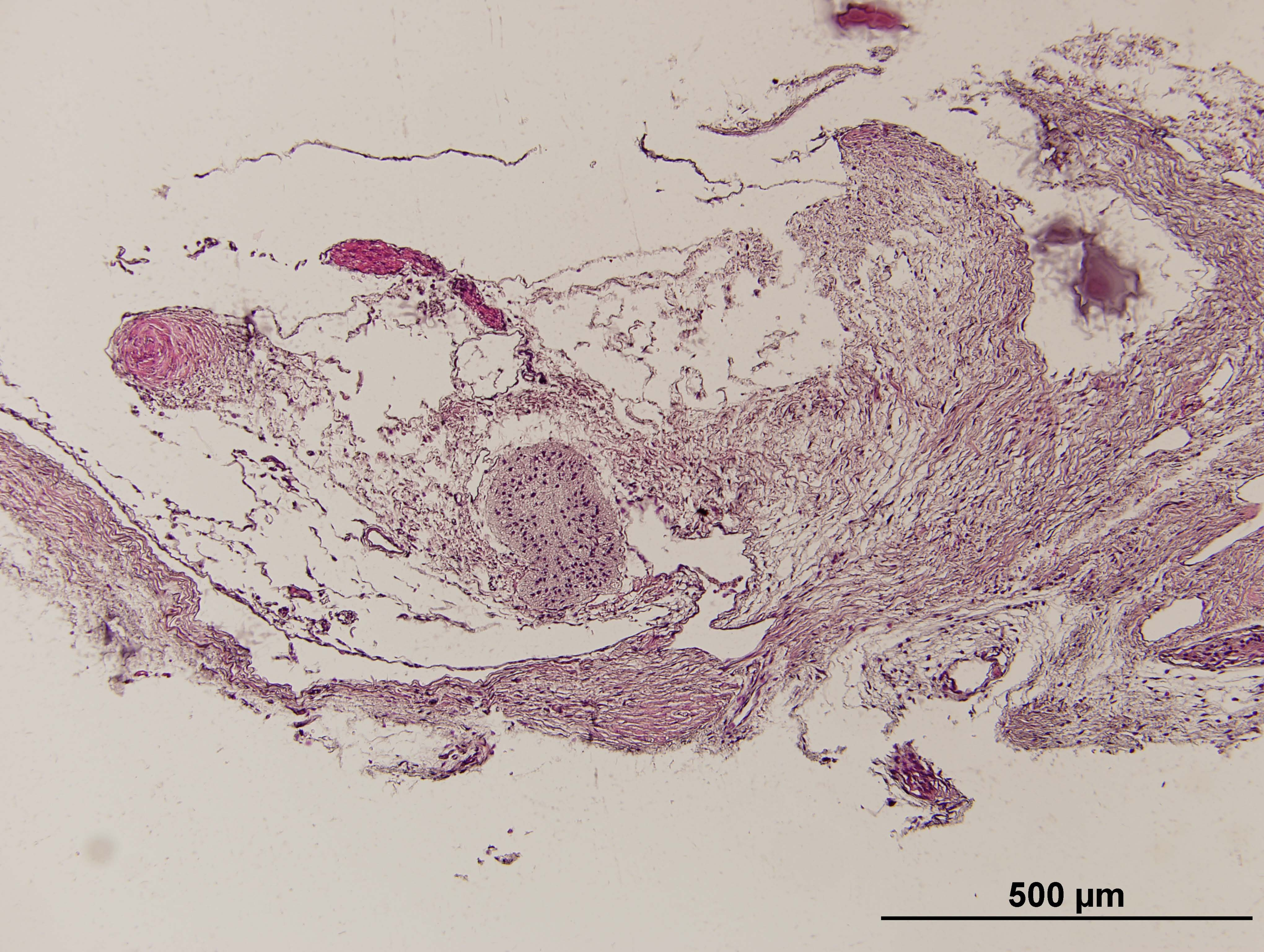
500 µm

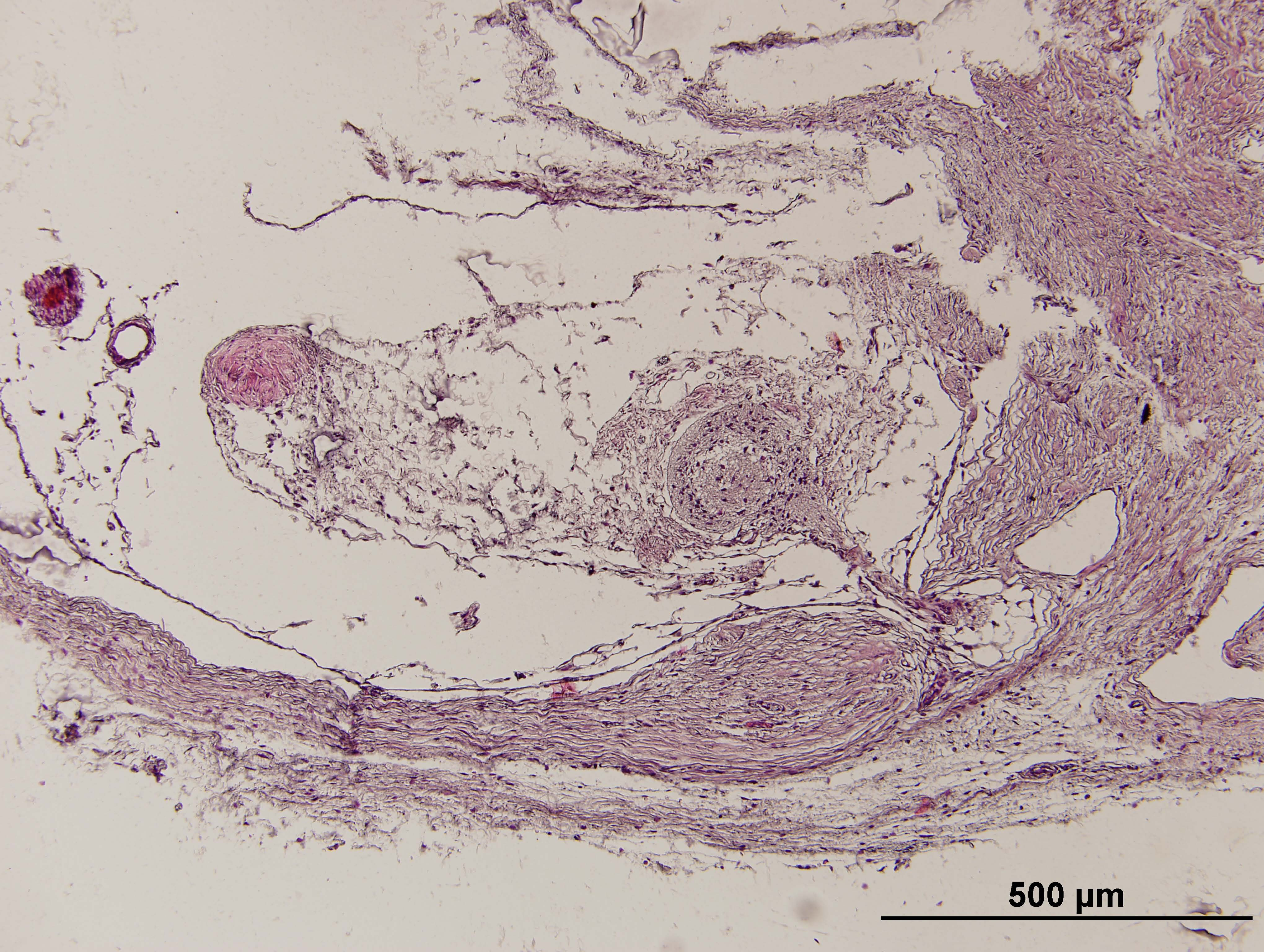
500 µm

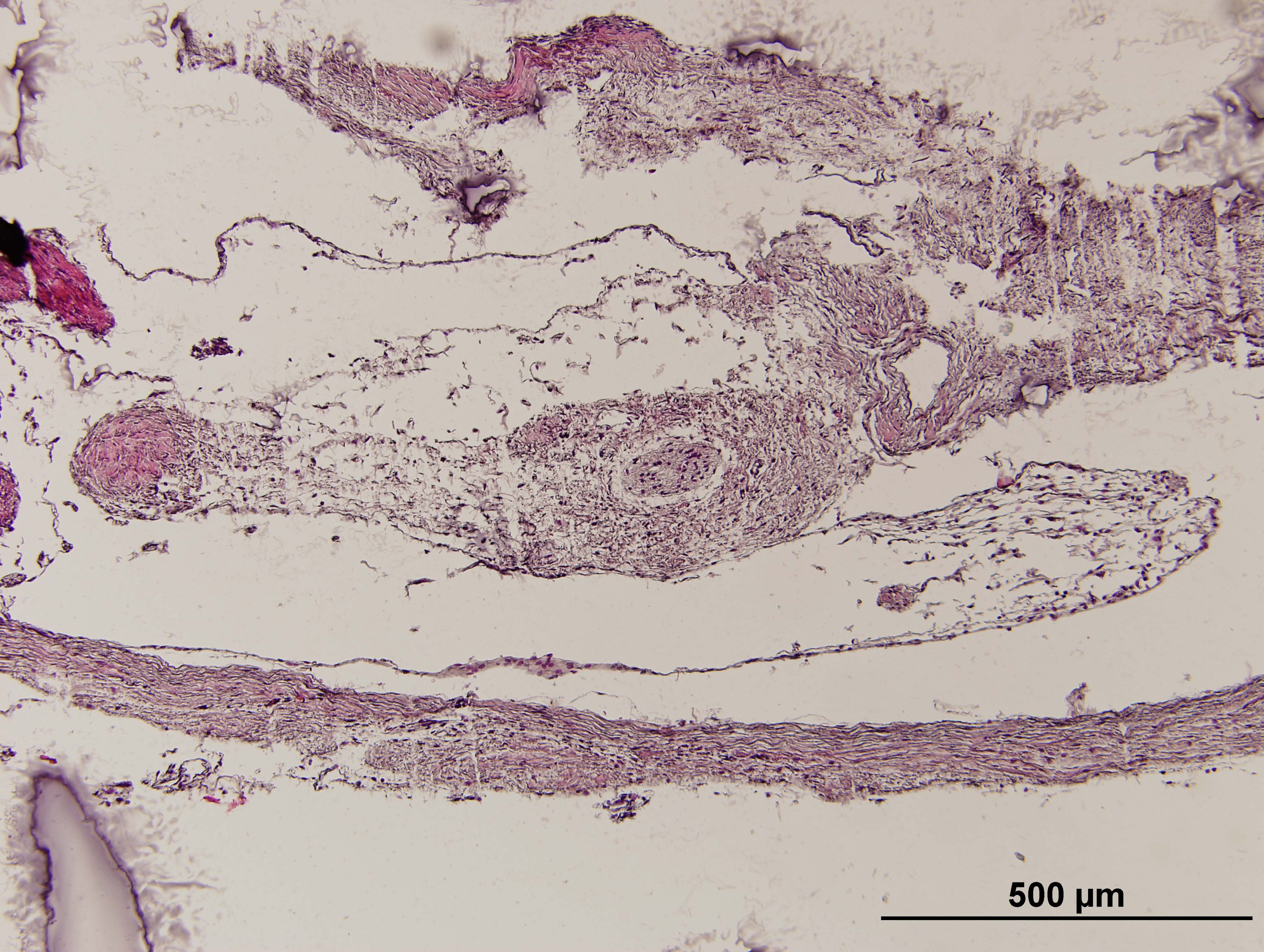
500 µm

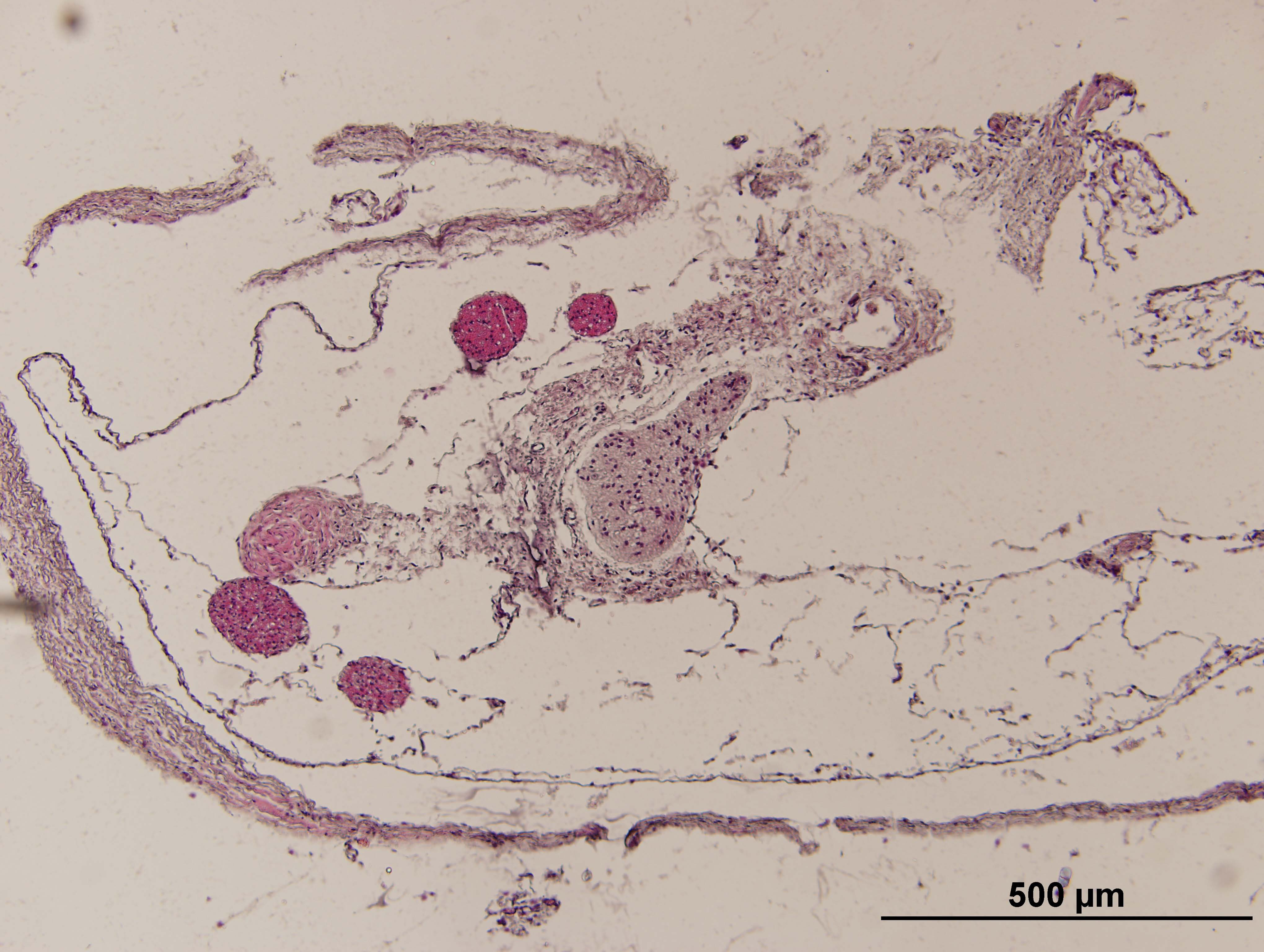
500 µm

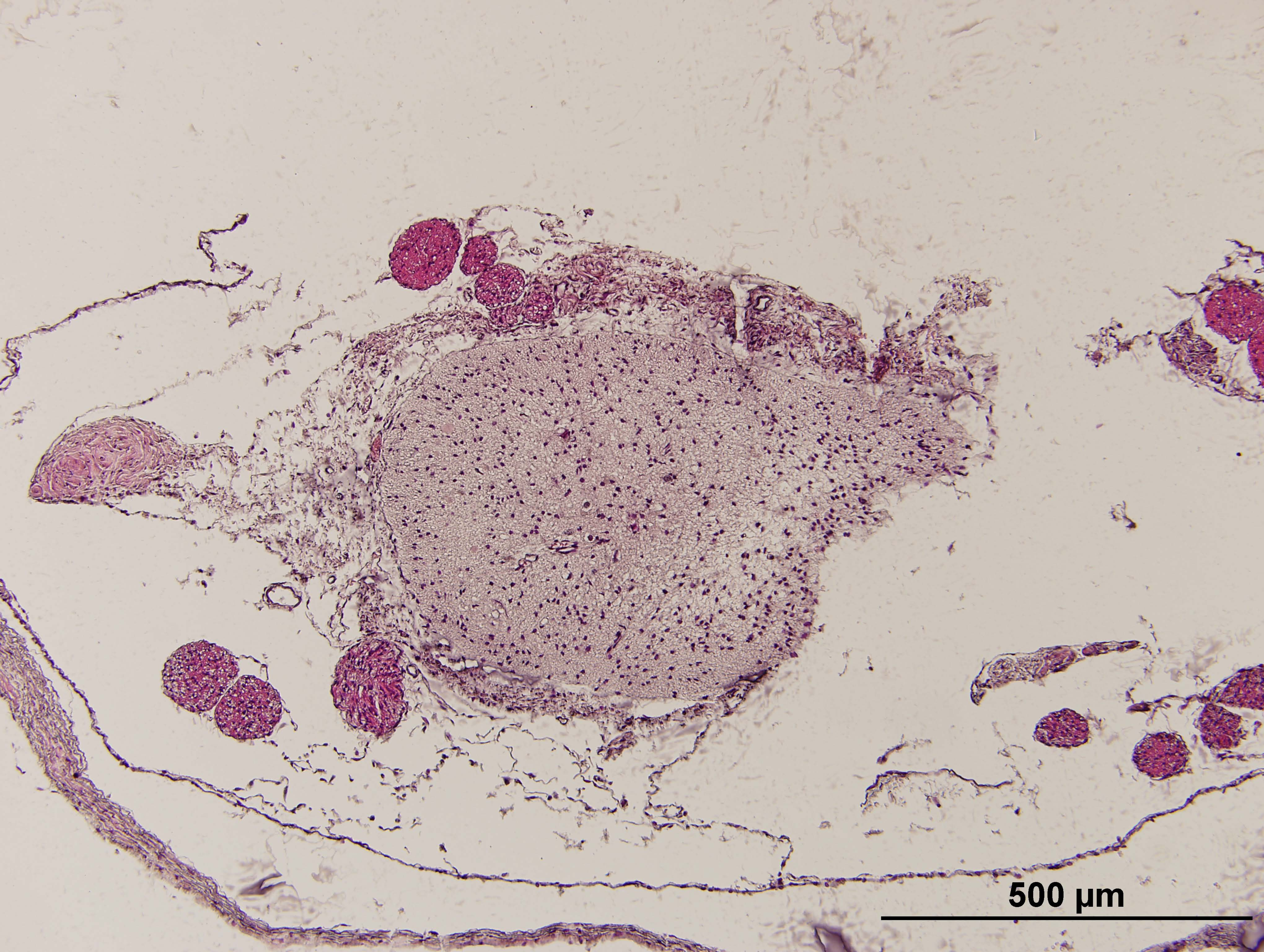
500 µm

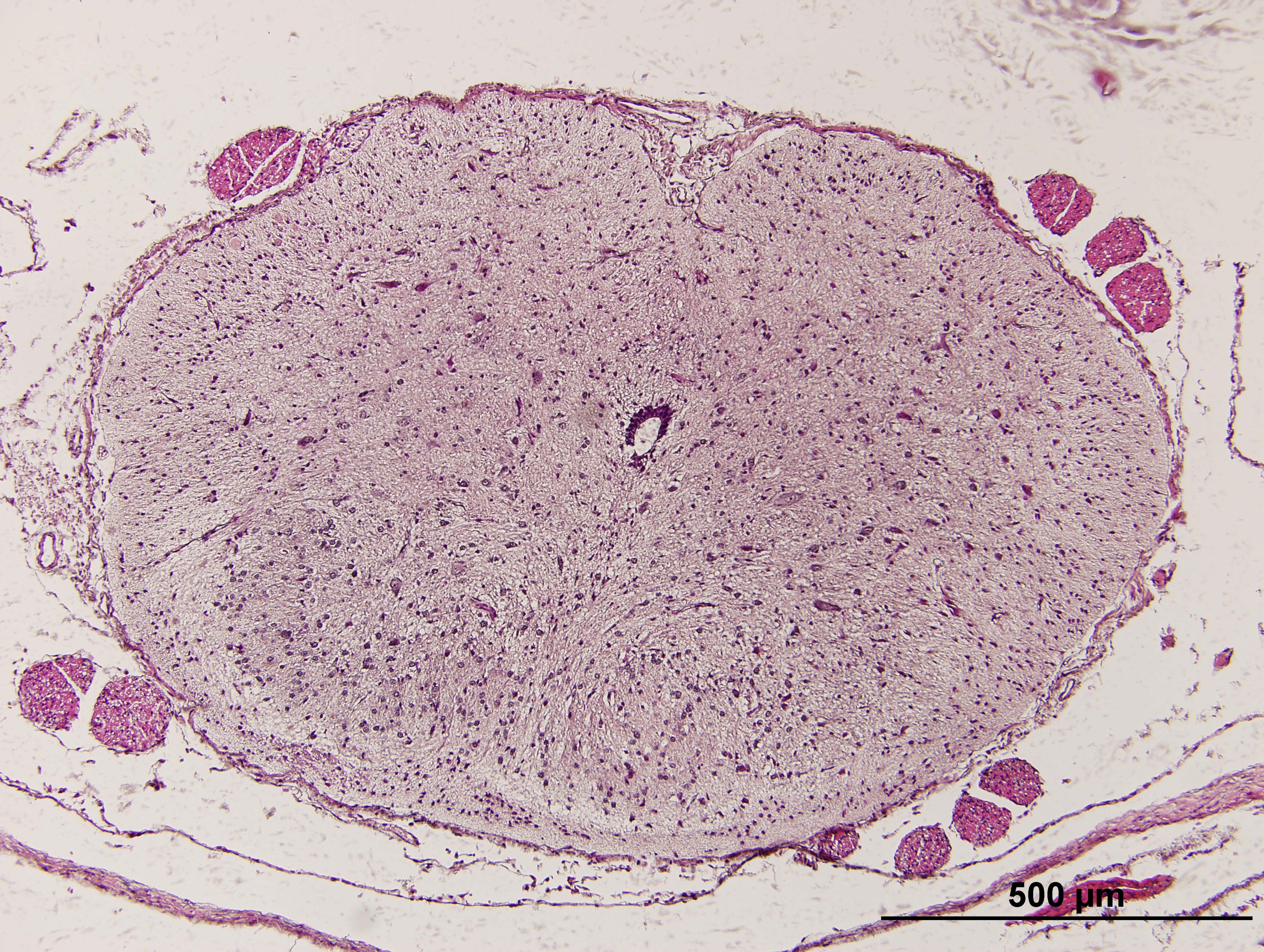
500 µm

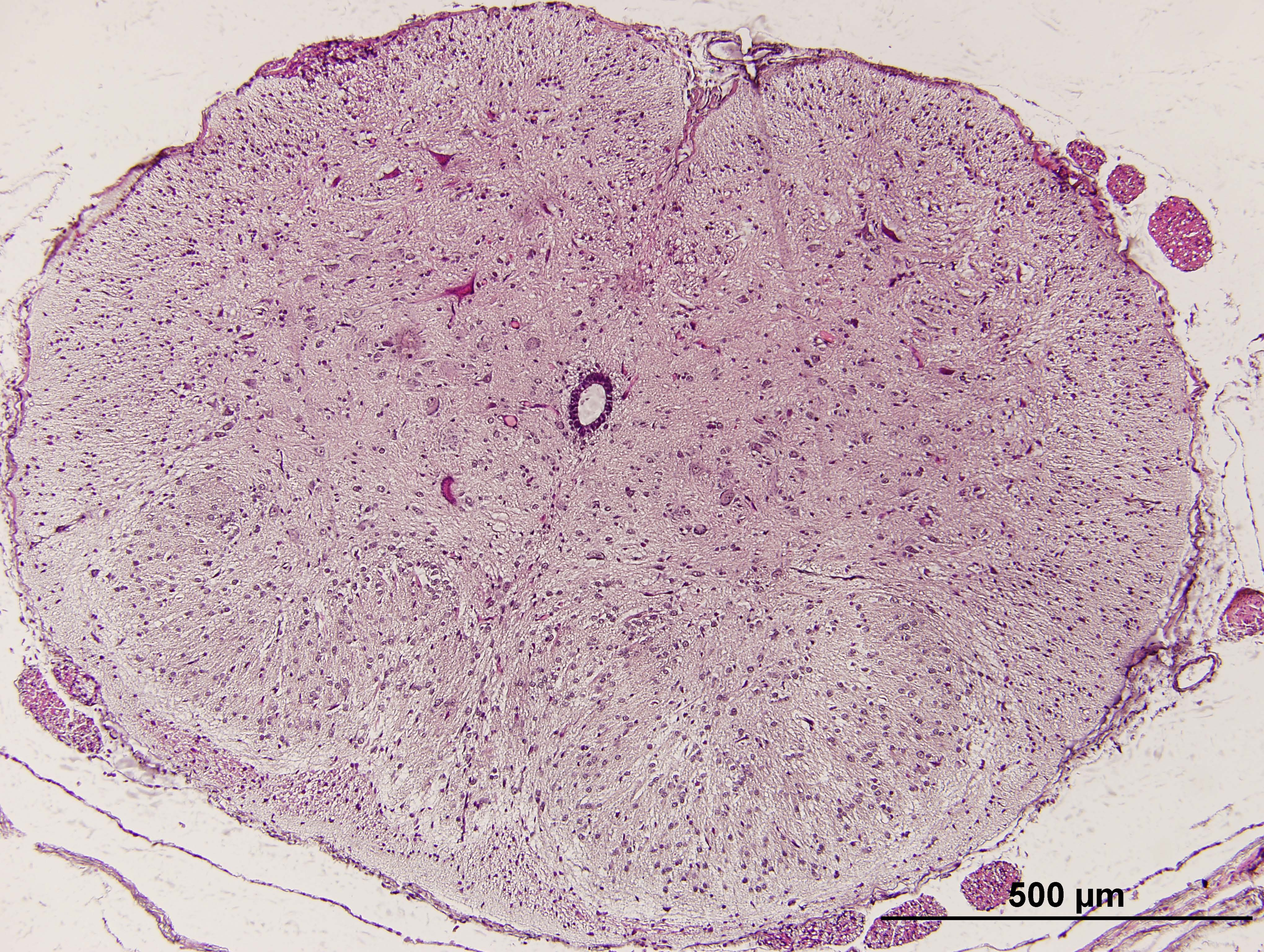
500 µm

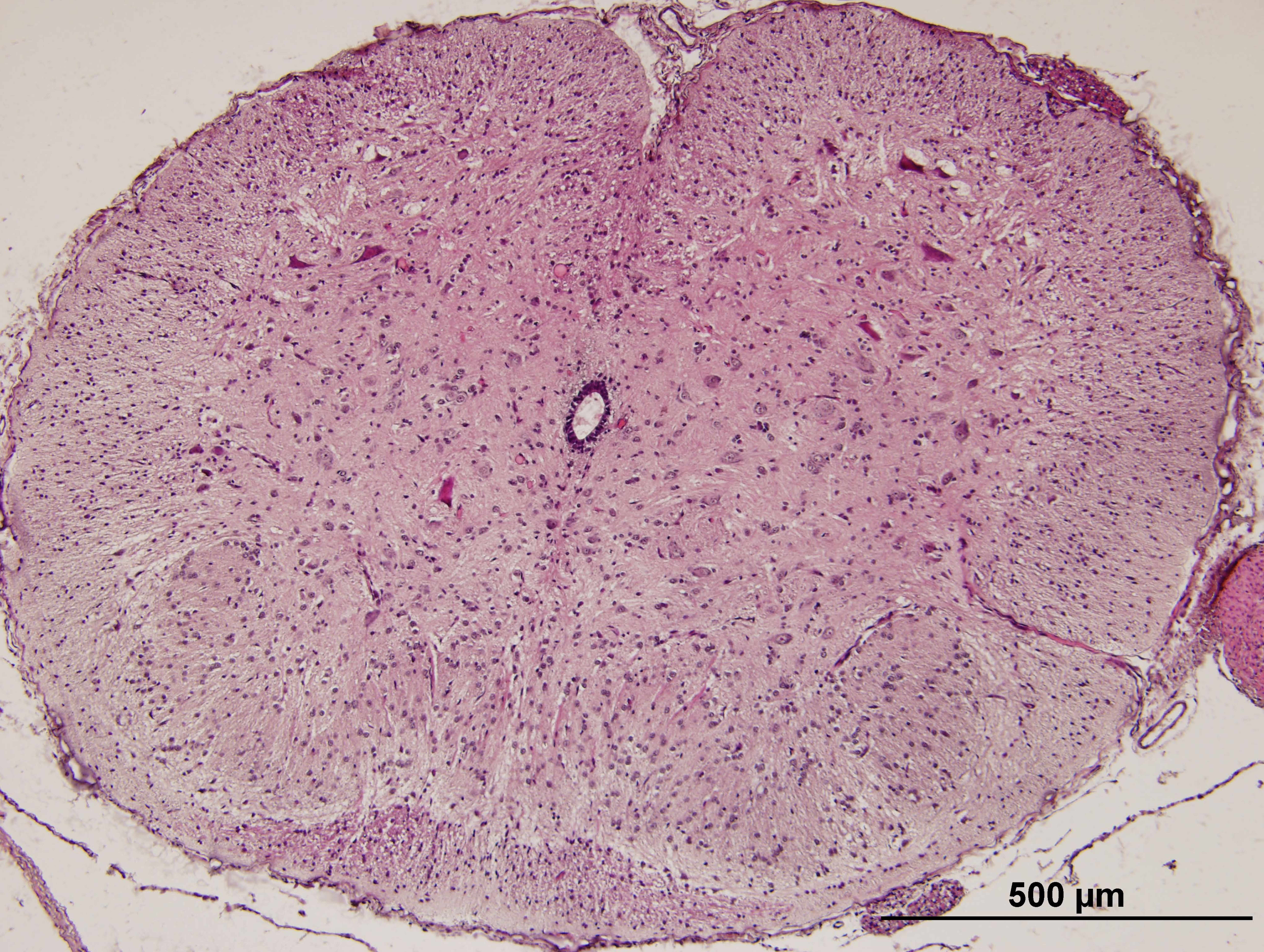
500 µm

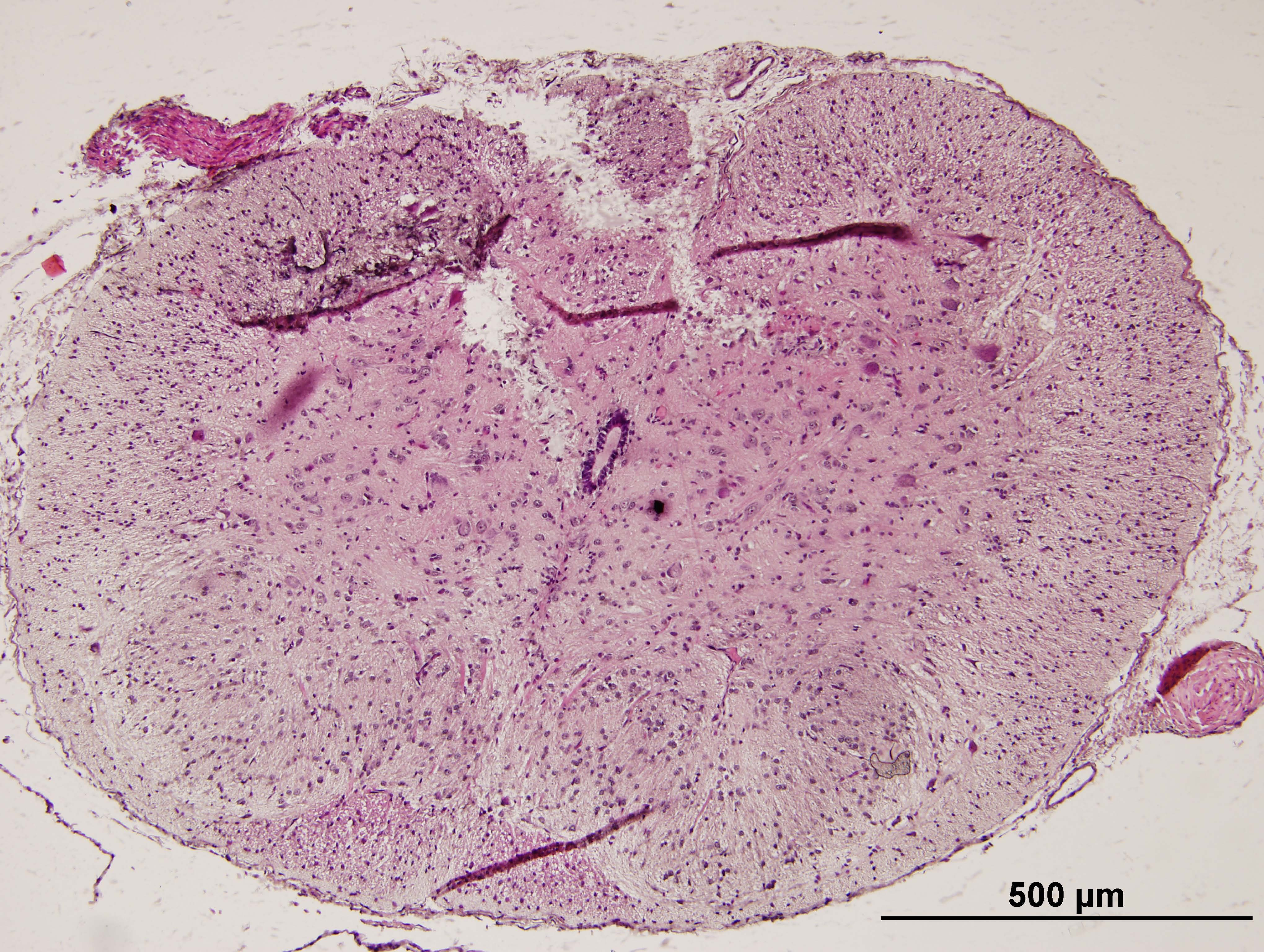
500 µm
