# Supplementary material for: A bipedal mammalian model for spinal cord injury research: The tammar wallaby
Source: F1000Res. 2017 Jun 15;6:921. [Version 1] doi: 10.12688/f1000research.11712.1 (PMC5497825; doi:10.12688/f1000research.11712.1)
Supplement: Archived video and histological data. [file f1000research-6-12654-s0000.tgz › 00cb5ab8-8bc4-420a-83a6-f865b6698448_Dataset_1.zip!/Dataset 1/P7-60 injury centre sections.pdf]

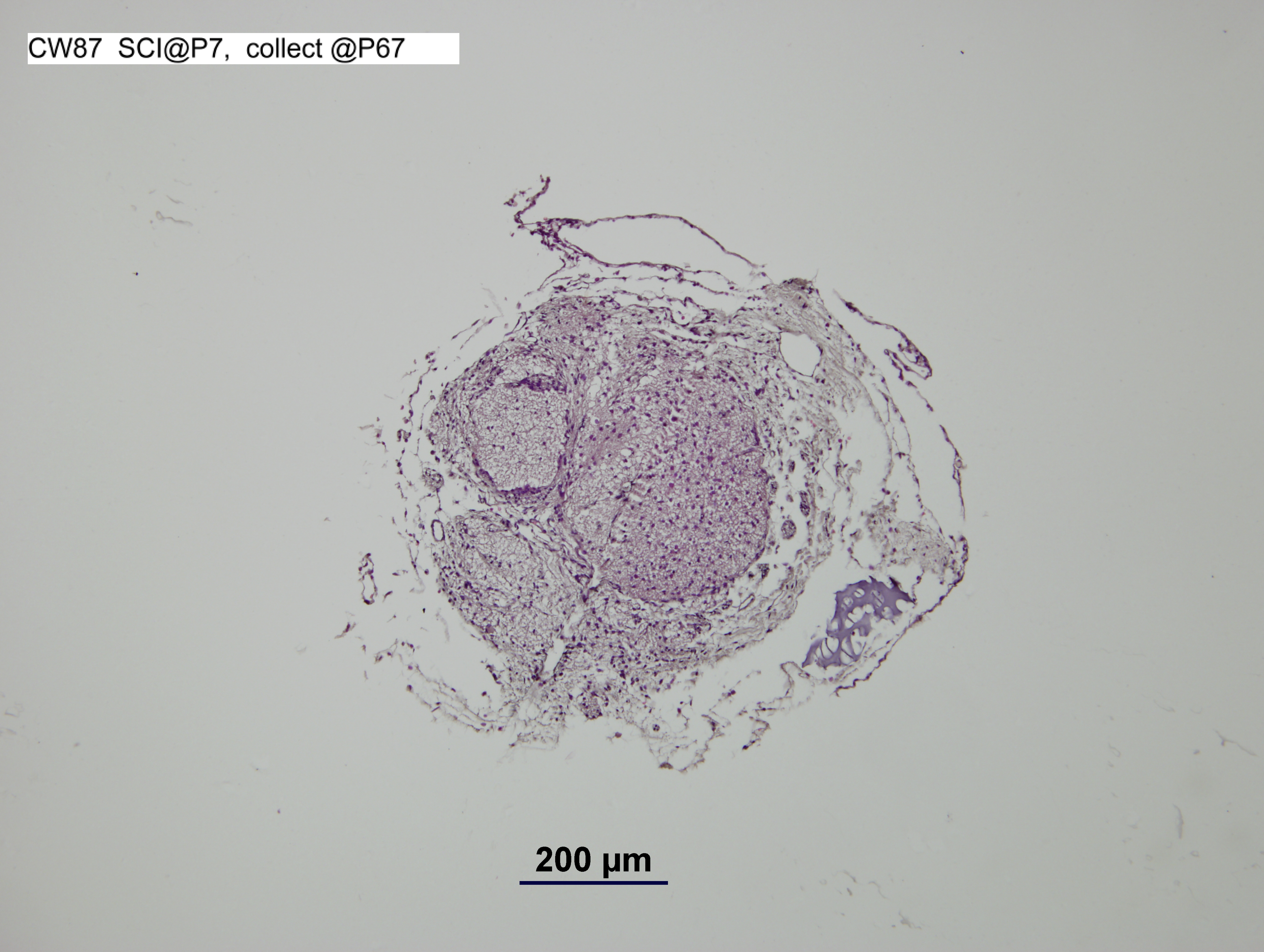
CW87 SCI@P7, collect @P67
200 µm

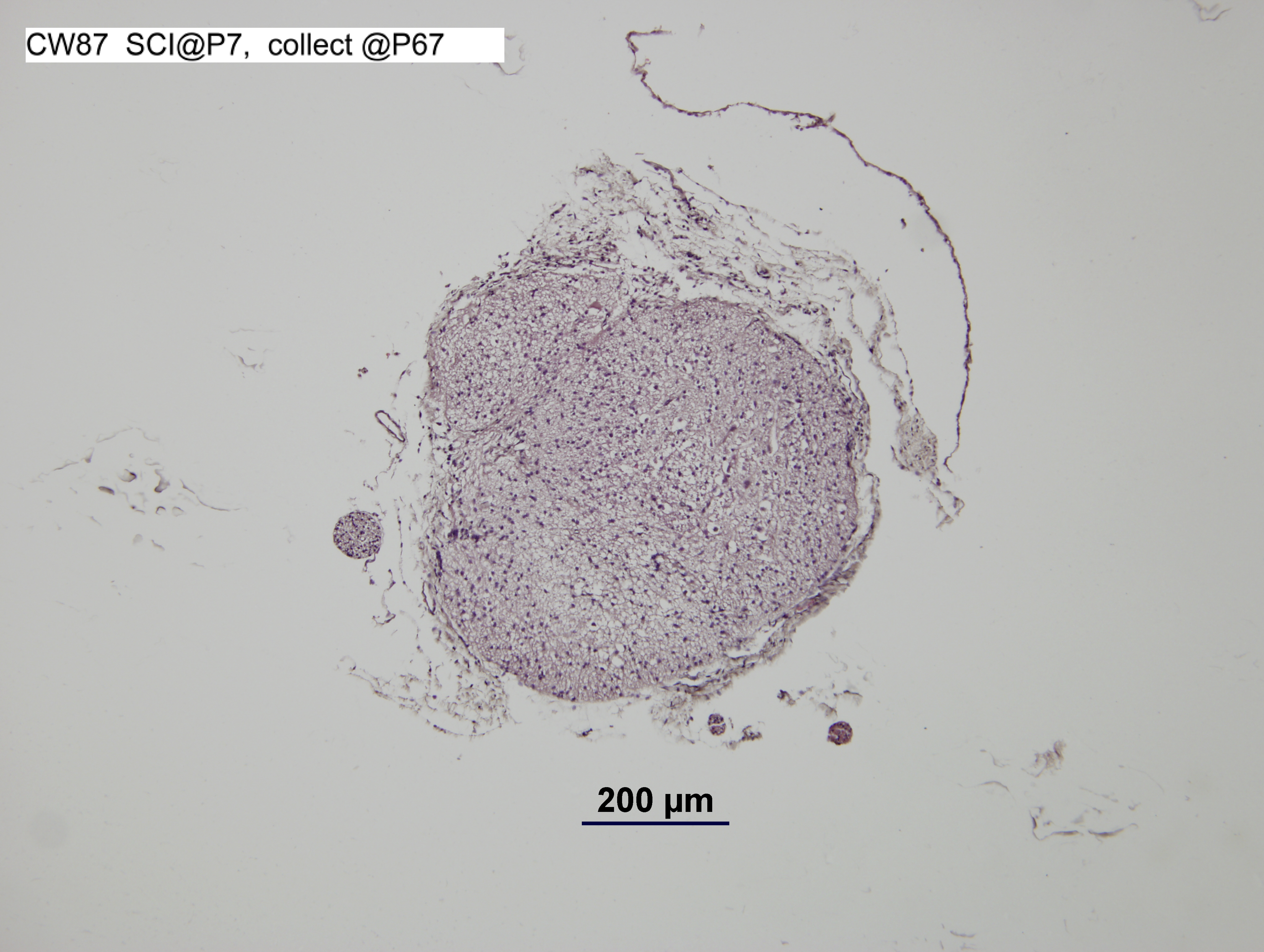
CW87 SCI@P7, collect @P67
200 µm

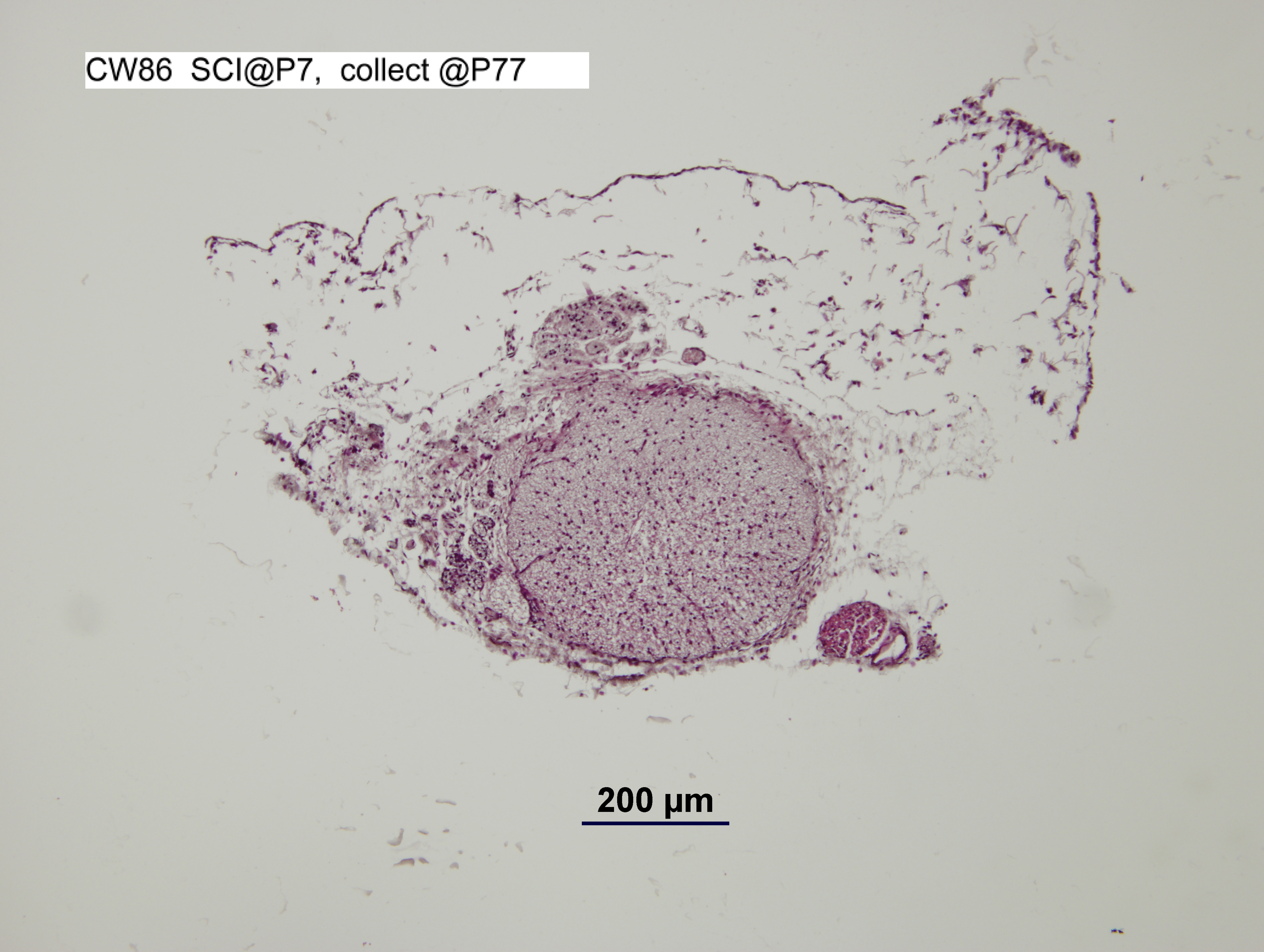
CW86 SCI@P7, collect @P77
200 µm

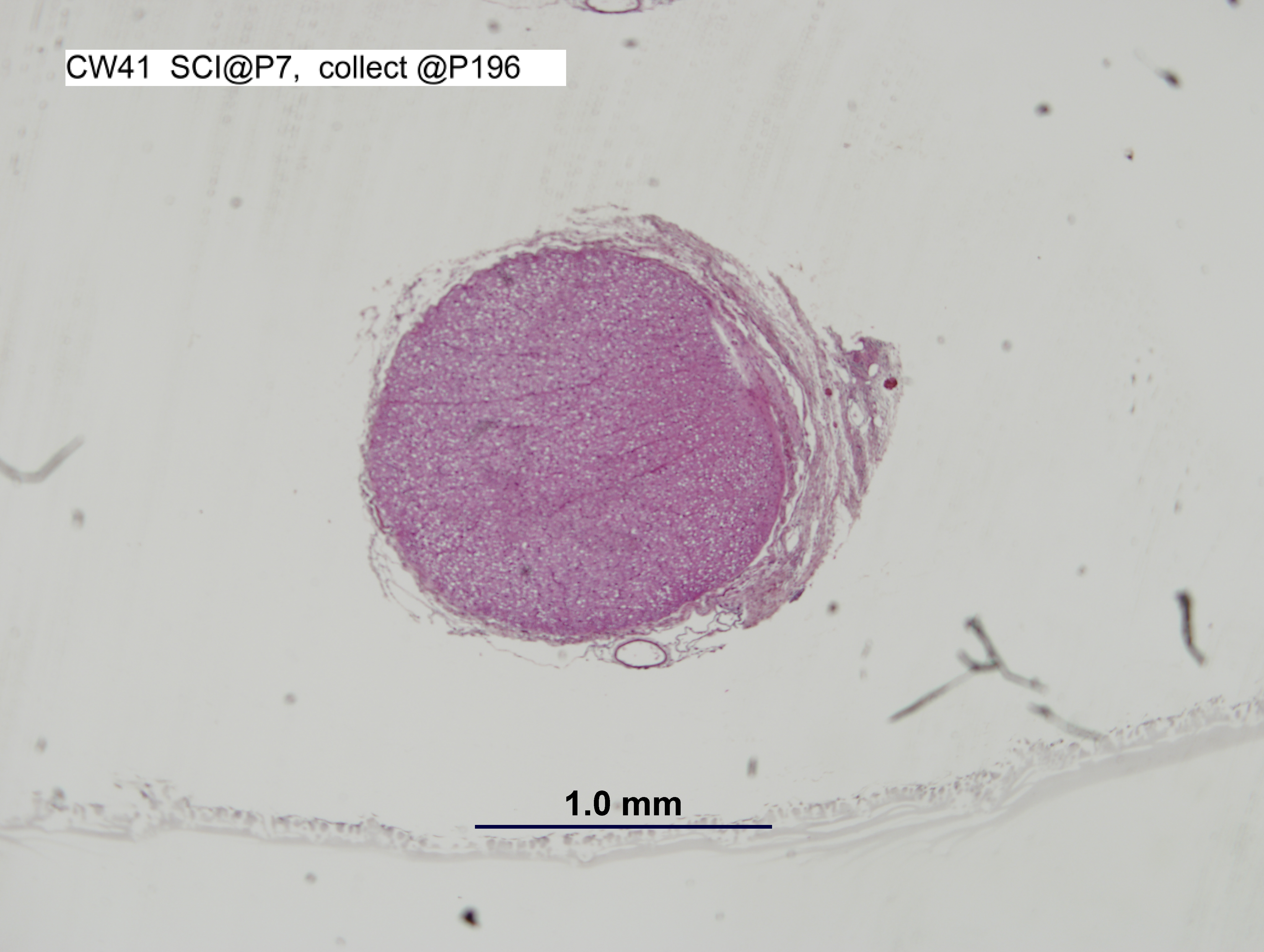
CW41 SCI@P7, collect @P196
1.0 mm

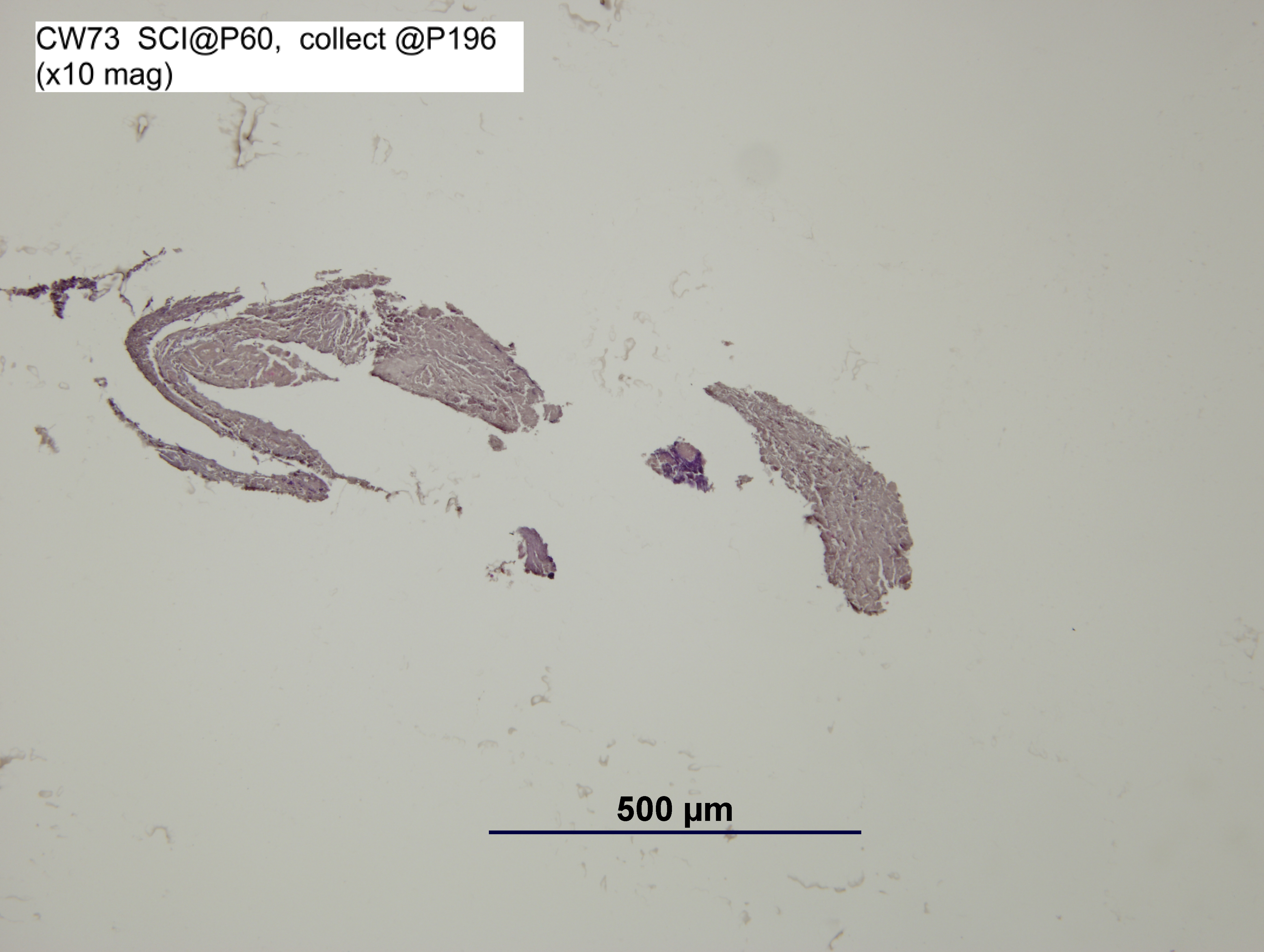

CW73 SCI@P60, collect @P196
(x10 mag)

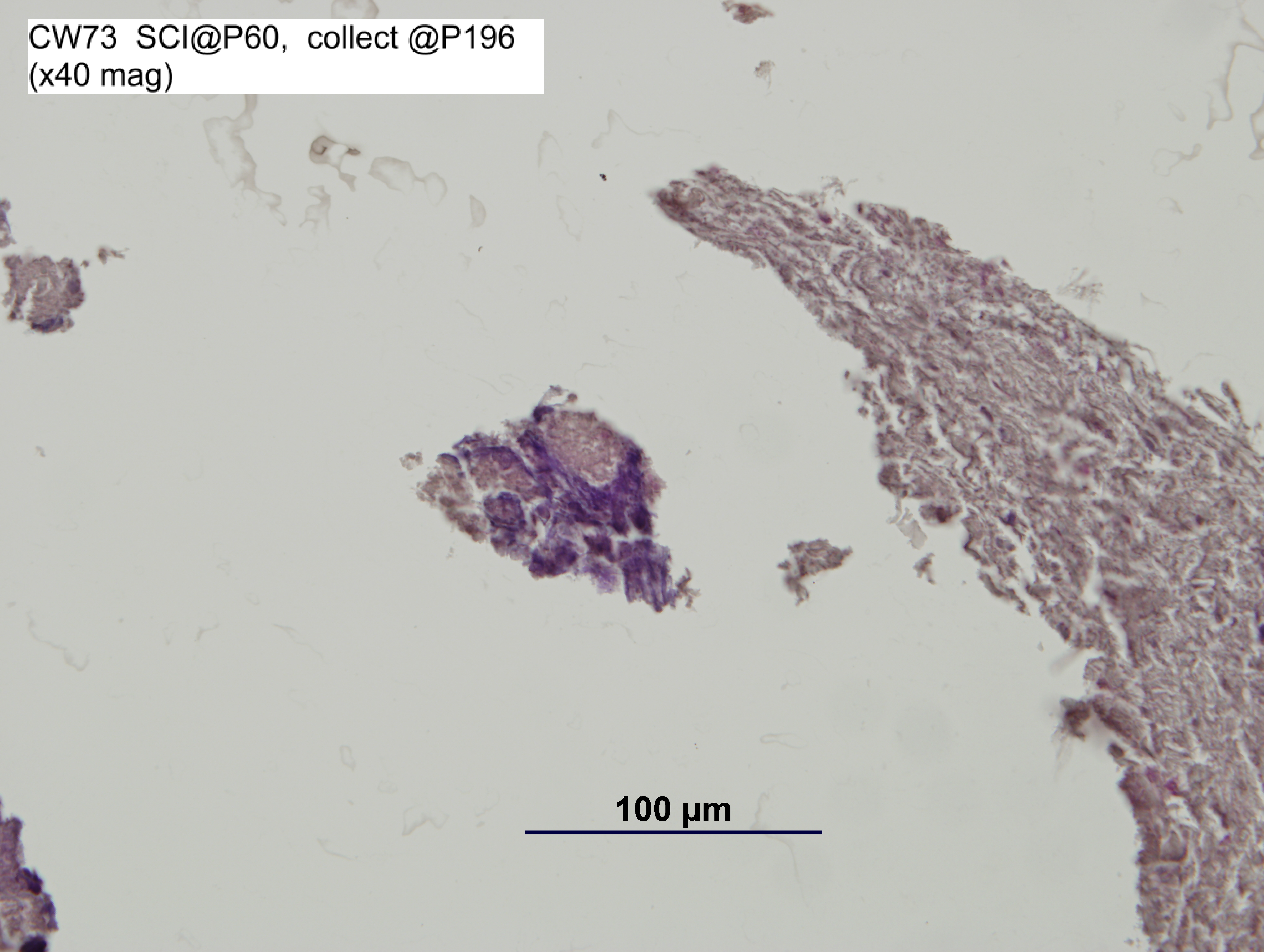
CW73 SCI@P60, collect @P196
(x40 mag)
100 µm
