# Supplementary material for: A bipedal mammalian model for spinal cord injury research: The tammar wallaby
Source: F1000Res. 2017 Jun 15;6:921. [Version 1] doi: 10.12688/f1000research.11712.1 (PMC5497825; doi:10.12688/f1000research.11712.1)
Supplement: Archived video and histological data. [file f1000research-6-12654-s0000.tgz › 00cb5ab8-8bc4-420a-83a6-f865b6698448_Dataset_1.zip!/Dataset 1/Saunders et al Raw data files.pdf]

Head length and body weight measurements

08.07.15					
Animal	Age (days)	Head Length (mm)	Body Weight (g)		
CW68	63	30.06	21.25		
CW70	62	29.84	21.94	pouch empty	16.07.15
CW76	70	32.84	31.59		
CW77	64	30.41	24.8		
CW80	82	37.22	36.65		
CW81	85	38.21	39.5		
CW83	86	38.61	44.55		
CW84	86	38.52	44.1		
CW90	90	39.72	46.34		
CW69	67	31.63	25.163	pouch empty	10.05.15
CW71	59	28.77	19.28	dead in pouch	08.07.15
CW72	66	31.15	24.37	pouch empty	17.05.15
CW73	59	28.68	18.06	killed	21.05.15
CW74	66	31.41	23.96	pouch empty	17.05.15
CW75	66	29.55	21.12	pouch empty	17.05.15
CW78	81	36.66	35.94	pouch empty	17.05.15
CW79	77	35.47	36.12	pouch empty	17.05.15
09.07.15					
CW82	93	41.09	49.7	pouch empty	17.05.15
CW85	90	39.72	46.34		
CW91	3	8.5	0.65		
CW93	4	8.64	0.716		
CW92	4	8.89	0.948		
CW94	5	9.37	0.896		
CW87	6	9.67	1.034		
CW86	7	10.12	0.993		
CW89	7	9.78	1.009		
CW88	7	10.18	1.121		
CW90	7	9.79	1.022		
16.07.15					
CW93	72	33.68	31.48		
CW91	72	32.95	30.38		
CW89	73	33.91	30.86		
CW94	74	34.24	32.6		
CW92	74	34.27	30.95		
CW87	75	34.59	30.87		
CW86	76	34.78	32.03		
CW88	78	35.65	36.62		
CW88	79	36.11	35.88		

04.08.15				
CW68	162	154	57.59	152
CW76	169	157	58.68	205.1
CW77	163	145	55.14	170.9
CW80	181	177	64.19	251
CW81	184	183	66.11	266
CW84	185	171	62.66	252
CW85	189	175	63.38	294.2
CW91	91	95	39.91	48.55
CW93	92	97	40.61	51.1
CW92	92	96	40.39	50.3
CW94	93	101	42	55.6
CW87	94	96	40.32	49.8
CW86	95	93	39.5	47.5
CW89	95	95	40.95	52.5
CW88	95	102	42.38	59.1
CW90	95	100	41.53	57.7

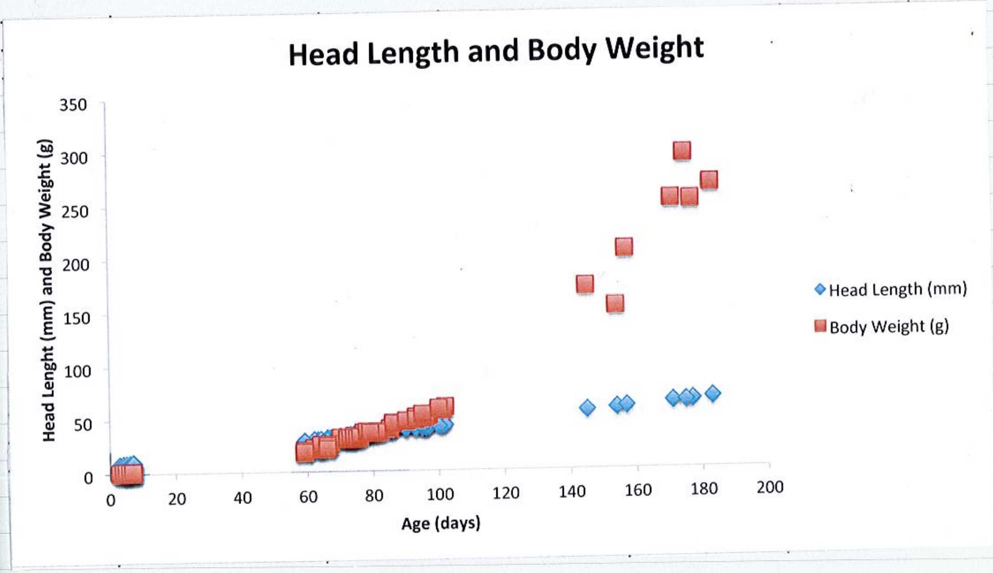

O = up to 5
☆ = up to 10
★ = up to 20
☆ = up to 50

CW65 Control

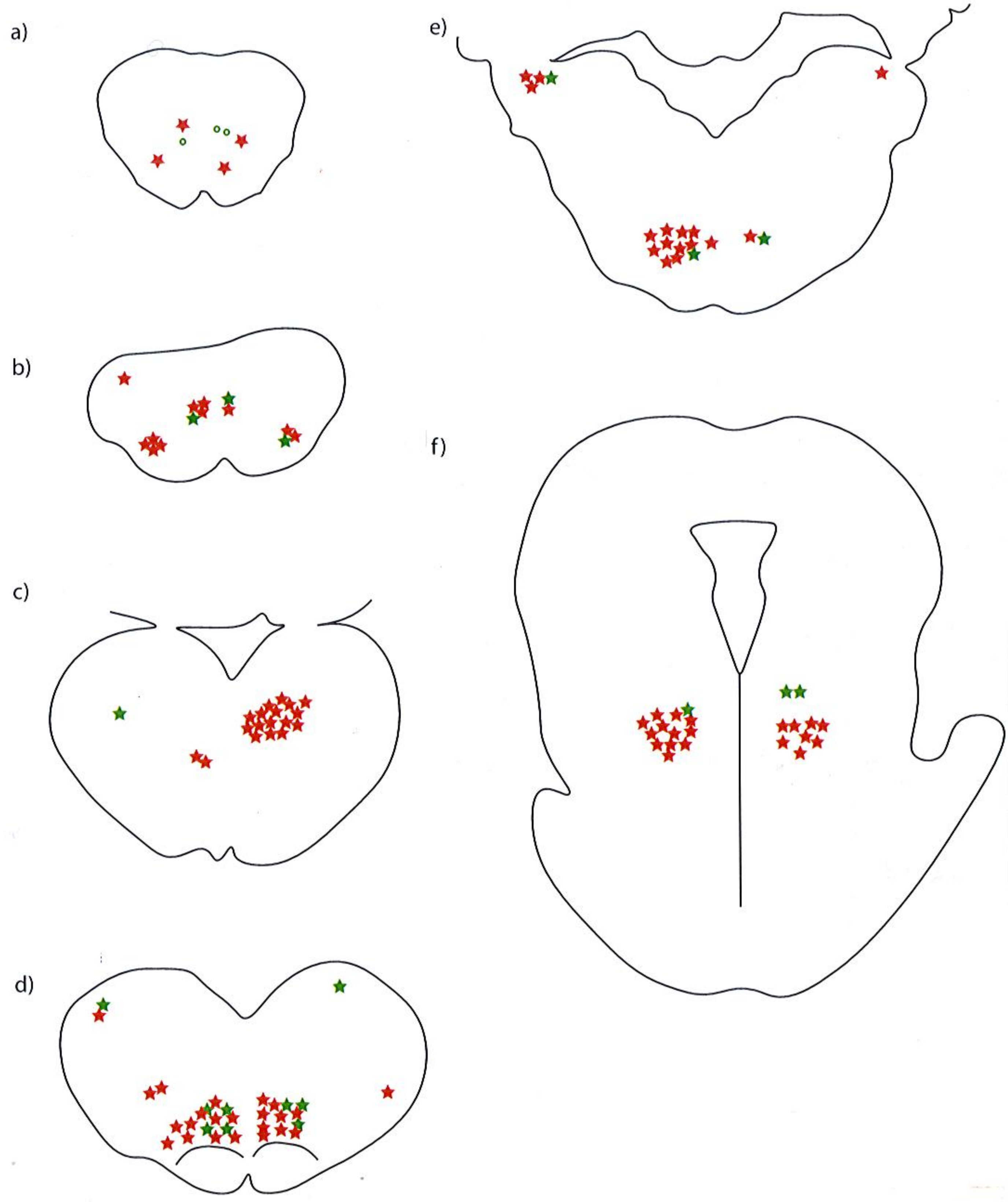

CW65 Control

CW65 Control Slide	Left Red	Ventral horn Green	Right Red	Ventral Horn Green
1.1	8			
1.2	11	1	1	
1.3	17	1	3	
1.4	8	3	10	1
1.5	18		6	1
1.6	22	1	12	
1.7	8	1	12	1
1.8	17	3	4	
1.9	16		3	
2.1	42	4	13	3
2.2	19		15	2
2.3				

Slide	dorsal horn	Ventral horn	Dorsal horn	Ventral Horn
2.4	14	15		11
2.5				
2.6	8	26		17
2.7				
2.8	25	30	10	35
3.1				
3.2	26	51	28	42
3.3				
3.4				
3.5	8	56	5	43
3.6	3	43		27
3.7		66		27

Slide	RGc	RV	Green	Lat/above RV	RGc	RV	Lat/above RV	Green
4.1	35				28			
4.2		59			16			1
4.3		76	1		13			
4.4		105		21		16		
4.5		123		23		19		

Slide	RGc	RV	Lat/above RV	VstM	VstL	RGc	RV	Lat/above RV	VstM	VstL	Green	Midline
5.1		123			3			23				
5.2		100	28		5			25	12			
5.3		107	18	4	7			24	10			
5.4		114	28	8	6		47 (3g)		13	1 2 (1g)		
6.1		126	26 (1)	15(1)	6			44	10	5 (2g)		
6.2		123	18	30 (5)	12 (2g)	6 (1g)	56 (5g)		2 (1g)	3 (1g)		
6.3	11	145		28 (8)	5	6	41 (9g)		21 9 (2)	6 (2)		
6.4	dried out											
7.1		225		24	2		54 (6)			13	9	
7.2		182		8				46		38		
7.3		186					27 (10g)		28 (10 more med))			
7.4		124						20		8		
8.1		101 (22g)				16	24 (8g)			7	21 (16g)	
8.2		98				14		26				
8.3		110				12		26			15	
8.4	34 (5g)	101			2	42		25				
9.1	dried out											
9.2	46	87			17 (8g)	14		7				
9.3	41	66			21 (5g)	4		13				
9.4	dried out				VStS					VstS	14	
10.1	34	36 (1g)			63 (12g)			5			3	
10.2	21 (4g)	89			86 (19g)			5			3	
10.3	6, 8 (lat)	29			38 (17g)			14			7	
10.4	dried out											

Slide	RGc	Lat RGc	RV	VstM	VstL	VstS	RGc	Lat RGc	RV	VstM	VstL	VstS
11.1	31 (7g)	17	45							13	6	
11.2	22 (6g)	3 (10g)	28					31		31		
11.3	24 (11g)	41 (12g)	17					50		6		
11.4	18 (6g)	43 (5g)	17					50		8		

Slide	RGc	Lat RGc	Sup RGc	RV	VstM	VstL	VstS	RGc	Lat RGc	Sup RGc	RV	VstM	VstL	VstS
12.1	22	66 (9g)	50					1		54				
12.2	18 (18g)	73 (11g)	61 (10g)	12					59 (3)			7		
12.3	14 (7g)	62 (2g)	46 (12g)	12					66 (4)			6		
13.1	22	54	71 (11)	8						64				
13.2	12 (9g)	40	46 (9g)							67				
13.3	24 (12g)	25	30 (10g)							58				
14.1	24 (4g)		42 (7g)							68				
14.2	22 (9g)		63 (20g)							62				
14.3	36 (9g)		48							59				
15.1	48 (18g)		19							43	7			
15.2	45 (9g)		24							52	9			
15.3		39 (11g)								44	7			
16.1	dried out													
16.2			13 (5)							54	14			
16.3										19	3			

Slide	RP	RP
17.1	2	6
17.2		
17.3		
18.1	14	

CW66 Control

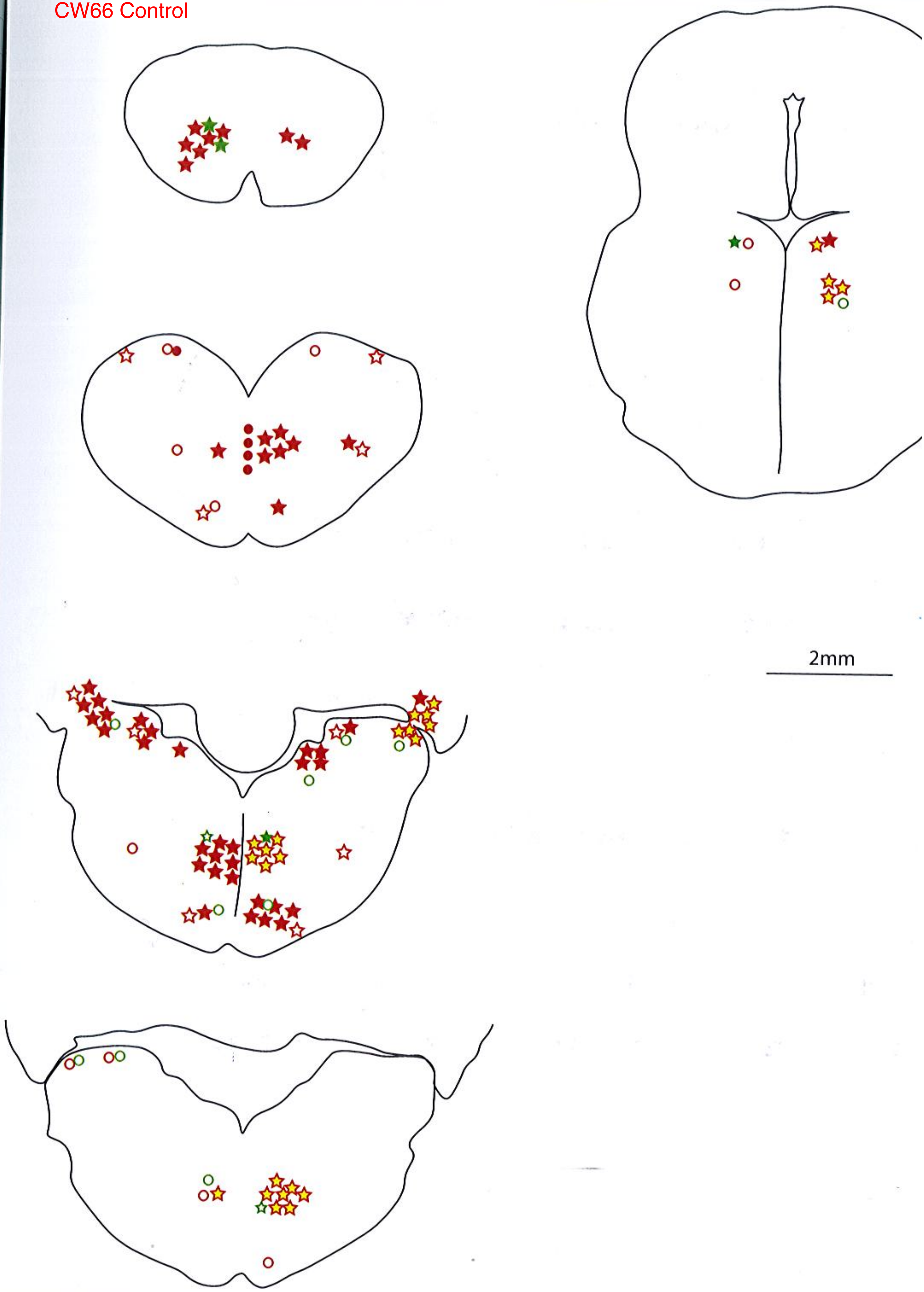

CW66 Control Slide	Left Red	Ventral horn Green 2 (medial to midline)	Right Red	Ventral Horn Green
38.1	8		15	
38.2	10		11	
38.3	2		6	
38.4	5		6	
37.1	11	3		
37.2	36	4		
37.3	17	5		
37.4	15	6		
37.5	13	9		
37.6	10	11		
total	127	40	38	

	VstL	VstM	Med to VstM	RV	RGc	Lat to RGc	VstL	VstM	Med to VstM	RV	RGc	Lat to RGc	midline
36.1	2				4						4	2	
36.2	4				2							2	4
36.3					2						1	3	
36.4	1				3							3	
36.5					2							4	
36.6						5						3	
35.1					3						13	1	
35.2					1						3		
35.3		3									6		
35.4		1									7	1	
34.1		1			2		3				14		
34.2	3	1					5	3			19	1	
34.3											15	5	
34.4											12	1	
Total	10	6			19	5	8	3			94	26	4
33.1	6	13		6				10	19 (1g)		25 (1g)		
33.2	9	7		7	2		6	7	12		19	1	
33.3	8	5	5					7	11		23		2
33.4					3g		9 (1g)	2 (1g)	6	1	27 (3g)	5	
32.1			1	3	3				1	8	22		
32.2	3	11	3	3g	6 (3g)			1	2	11 (3g)	31		
32.3	4	23	3				1		1		32		
32.4		10	9					2	21	2	30		
Total	30	69	21	16	11	5	16 (1g)	29 (1g)	82 (1g)	22 (3g)	209	6	2
31.1					17		3			6	43		
31.2				2	31					2	45		
31.3				2	25		1			10	37		
31.4				5	9					5	29		
Total				9	82		4			23	154		
30.1	7				6		34				30		
30.2	6	1g			14		19				18 (2g)		
30.3	10			2	22(3g)		7				31 (3g)		
29.1	4				1 (1g)		88			8	18		
29.2	2 (3g)			1			71			11	21		
29.3	12			1g			42			7	11		
28.1					2		2			1	37 (4g)		
28.2					3		19			1	41 (3g)		
28.3				2	2		75			4	27		
	VstL	VstM	Med to VstM	RV	RGc	Lat to RGc	VstL	VstM	Med to VstM	RV	RGc	Lat to RGc	midline
[illegible]	[illegible]	[illegible]	[illegible]	[illegible]	[illegible]	[illegible]	[illegible]	[illegible]	[illegible]	[illegible]	[illegible]	[illegible]	[illegible]

	VstL	VstM	Med to VstM	RV	RGc	Lat to RGc	VstL	VstM	Med to VstM	RV	RGc	Lat to RGc	midline
27.1	1	1			5 (1g)						45 (3g)		
27.2					5					1	39 (4g)		
27.3					5						53 (1g)		
26.1					1						42		
26.2											29		
26.3					31						31		
Total	1 (3g)	1 (1g)			47 (1g)					1	244 (8g)		
25.1					1						32		
25.2					1						31		
25.3					2						52		
Total					4						115		
											359		

	RP	above RP	RP	Above RP
24.1	4		32	
24.2	1		34	21
24.3			36	13
23.1	2	4 (8g)	13	3
23.2	1	3 (4g)	9	7 (4g)
23.3	2	4 (4g)	20 (4g)	5
22.1				3
22.2			5	3
22.3			1	6
21.1				2
21.2				1
21.3				3
20.1				1
20.2				5
20.3				
19.1				
19.2				
19.3				2
Total	10	11 (16g)	150 (4g)	75

CW52 Control

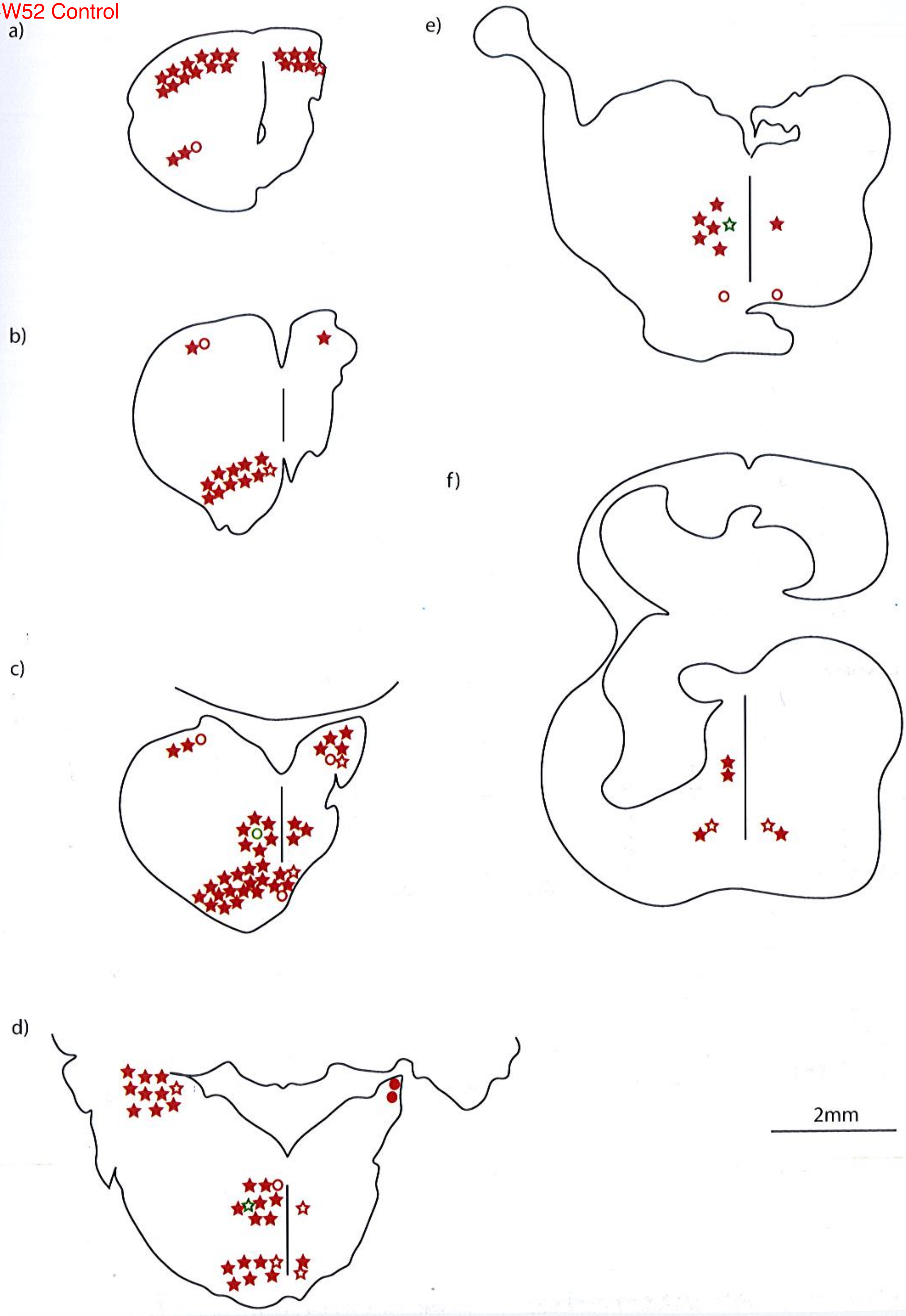

CW52 Control

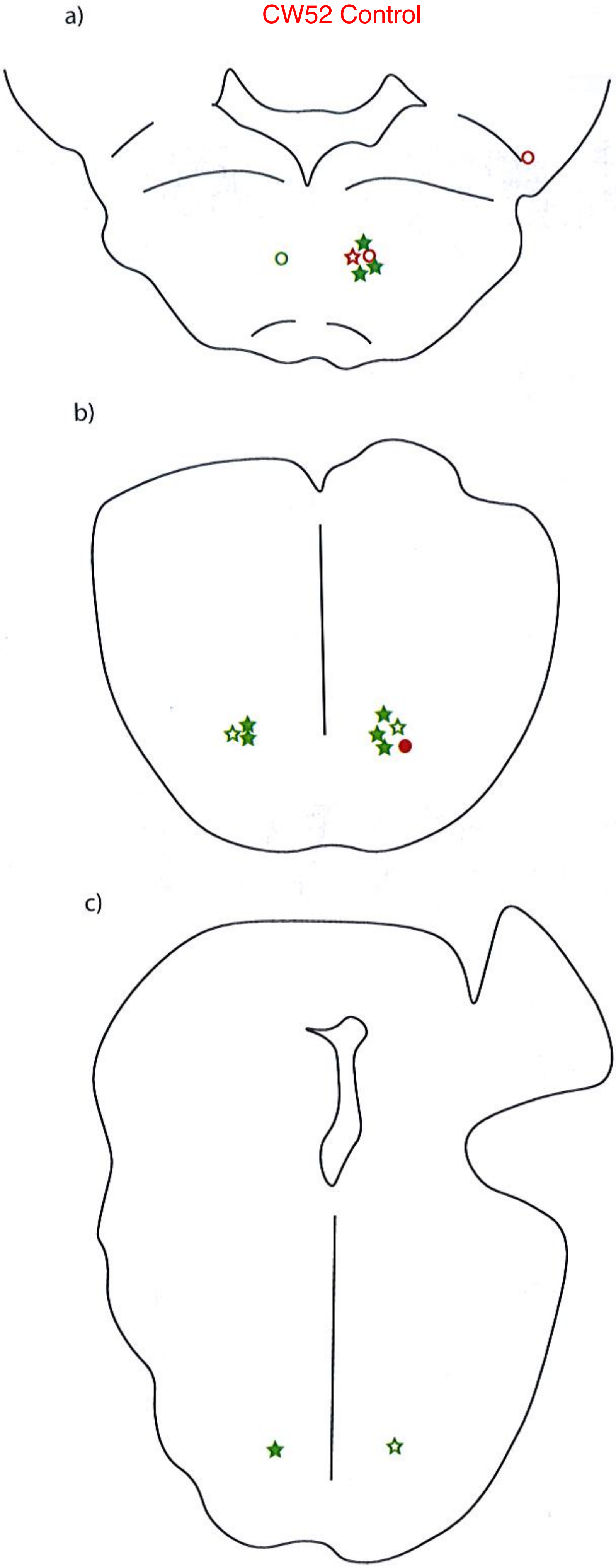

CW52 Control

	Slide	Left VstL or Coe	RGc	RV		Total	Right VstL	RGc	RV		
	1.1	10									
	1.2	7					1				
	1.3	7									
	1.4	7									
	1.5	13					3				
	1.6	3					5				
	1.7	2					2				
	1.8	9					2				
	1.9	6					5				
	1.10	4					5				
	2.1						3				
	2.2	6					2				
	2.3	8					4				
	2.4	8					8				
	2.5	6					11				
	2.6	15					7				
a)	2.7	15					3				
	2.8	11					8				
	2.9	15					5				
	2.10	23					5				
	3.1	25					10				
	3.2	50					9				
	3.3	55					7				
	3.4	58					10				
	3.5	46					10				
	3.6	73					15				
	3.7	52					12				
	3.8	57					16				
	3.9	60					21				
	3.10	52					25				
	4.1	28		5			27				
	4.2	29		6			21				
	4.3	31					12				
	4.4	23		3							
	4.5	VstL	RGc	RV		Total	VstL	RGc	RV		Total
	5.1	12		4		16					0
	5.2	9		9		18					0
	5.3	3		7		10					0
	5.4			19		19					0
b	5.5			21		21					0
	6.1			42		42					0
	6.2			43		43	6				6
	6.3			44		44	13				13
total		**24**		**189**		total	**19**				
	6.4	6		64		70	14				14
	6.5			44		44	17				17
	7.1	5		52		57	19				19
	7.2	13		65		78	5		16		21
	7.3	11		59		70	8		17		25
	7.4	8	10	56		74	3		15		18
c	7.5	3	19	24		46	18		19		37
	8.1		34	14		48	11		16		27
	8.2		24	31		55	2		25		27
	8.3		15	16		31		20	0		20
	8.4		12	40		52		27	3		30
	8.5		7	16		23		15	4		19
Total		**46**	**121**	**481**		total	**97**	**62**	**115**		
	9.1	3	7	36		46		3	13 (6 midline)		16
	9.2	11	3	7		21					0
	9.3	20	12	16		48					0
	9.4	29	14	28		71	1				1
	9.5	28	2			30					0
d	10.1	36	21	21		78					0
	10.2	36 damage	damage			36					0
	10.3	18	31	17		66			5		5
	10.4	10	12			22	1		5		6
	10.5		23	1		24		5	5		10
	11.1		20	3		23		3			3
Total		**191**	**145**	**129**	total		**2**	**11**	**28**		
	11.2		14			14					0
	11.3		11	3		14			4		4
e	11.4		22 (2 midline)			22		5			5
	12.1		21			21					0
	12.2		31			31		10			10
total			**99**	**3**	total			**15**	**4**		
	12.3		7			7		4			4
	12.4		31			31		8			8
	13.1			RP		0			RP	2	2
f	13.2				11	11				5	5
	13.3				2	2					0
	13.4				9	9				12	12
	14.1				4	4				8	8
total			**38**		**26**			**12**		**27**	

CW56 Control		Left				Right		
Slide		VstL or Coe	RGc (Red)	RGc (green)	Total	VstL or Coe	RGc (Red)	RGc (green)
	38.1							
	38.2						near central	1
	38.3							
	38.4							1
	38.5							
	36.6						in dorsal hor	2
	34.4							1
total								
	25.2					2 red	1	
	24.2					2 red	1	6
	24.3							5
	23.1						2	7
	23.2						2	10
	23.3						3	7
	22.1							4
	22.2						5	5
	22.3							5
	21.1							2
	21.2							
	21.3							3
**Total**			**0**	**0**		**4**	**14**	**54**
	20.1			18				26
	20.2			14				11
	20.3							
	19.1			20				20
	19.2			6				3
	18.1			3			1	10
**Total**				**61**			**1**	**70**
	18.2			13				8
	17.2			2				3

OWb1 SCT

Slide	RN	GRc	Irt	VstL	VstM	PN	RN	GRc	Irt	VstL	VstM	PN	RV
25.5	1												
23.4	1						1						
23.3							1						
22.2							1						
19.1													
18.4								1					
16.2		2						1					
16.3		1						1					1
16.4									1				1
16.5		3											
16.6								2					
15.2		3											
14.3		1											
14.4		1											
14.5		2											
13.2		1											
12.1		1											
12.2		1											
12.4			2										
11.1				2									
11.2				83						109			
11.3				210	56					164	70		
11.4				37						72			
10.3		1											
9.2		1						1					
9.3		1											
9.4		7											
9.5		2											
8.5		2											
7.1						1							
6.3												1	
6.4												1	
total	2	30	2	332	56	1	3	6	1	345	70	2	2

852

CW86 SCT –
none

CW87 SCT

Slide	GRc	RV	GRc	
12.3	1			
10.2			1	
9.1	1	1		
				4

CW92 SCT

none.

CW88 SCT

Slide	RN			RV			Irt			DPGi			GRc		
10.2															
9.6															
9.5															
9.4															
9.2													2		
9.1	2												2		
8.5		2	2			1							1		
8.4													4		
8.3													5		
8.2													1		
8.1															
7.3			1												
7.2															
7.1															
6.3															
6.2															
6.1															
5.3															
5.2															
5.1															
4.3															
4.2															
4.1															
3.4															
3.3															
3.2															
3.1															
2.4															
2.3															
2.3															
2.1															
Total	2	2	3	0	0	1	0	0	0	0	0	0	15	0	0

Slide	SubCD			SubCV			PnC			Coe			VstL		
10.2															
9.6															
9.5															
9.4															
9.2															
9.1															
8.5															
8.4															
8.3															
8.2															
8.1															
7.3															
7.2															
7.1															
6.3															
6.2															
6.1													1		
5.3															
5.2															
5.1															
4.3															
4.2															
4.1															
3.4															
3.3															
3.2															
3.1															
2.4															
2.3															
2.3															
2.1															
Total	0	0	0	0	0	0	0	0	0	0	0	0	1	0	0

Slide	VstM			Pr			VstM			VstL		
10.2												
9.6												
9.5												
9.4												
9.2												
9.1												
8.5												
8.4												
8.3										1		
8.2										8		4
8.1										10		2
7.3										6		2
7.2										6		2
7.1										16		1
6.3							4			6		
6.2										28		9
6.1										35		3
5.3							1			34		2
5.2										24		1
5.1												
4.3												
4.2												
4.1												
3.4												
3.3												
3.2												
3.1												
2.4												
2.3												
2.3												
2.1												
Total	0	0	0	0	0	0	5	0	0	174	0	26

Slide	Coe			PnC			SubCD			SubCA		
10.2												
9.6												
9.5												
9.4												
9.2												
9.1												
8.5												
8.4												
8.3												
8.2												
8.1												
7.3												
7.2												
7.1												
6.3												
6.2												
6.1												
5.3												
5.2												
5.1												
4.3												
4.2												
4.1												
3.4												
3.3												
3.2												
3.1												
2.4												
2.3												
2.3												
2.1												
Total	0	0	0	0	0	0	0	0	0	0	0	0

Slide	SubCV			GRc			DPGi			Irt	
10.2											
9.6											
9.5											
9.4				1							
9.2				3							
9.1											
8.5				2							
8.4											
8.3											
8.2											
8.1						1					
7.3											
7.2											
7.1				1							
6.3											
6.2											
6.1											
5.3											
5.2				1							
5.1										1	
4.3											
4.2											
4.1											
3.4										1	
3.3										3	
3.2											
3.1											
2.4											
2.3											
2.3											
2.1											
Total	0	0	0	8	0	1	0	0	0	5	0

	RV	GRc	
1			
		1	
1	1		
			4

	RV			Irt			DPGi			GRc			SubCD			SubCV			PnC			Coe			VstL			VstM			Pr			VstM			VstL			Coe			PnC			SubCD			SubCA			SubCV			GRc			DPGi			Irt			RV			Pr	
																																																															1				
																																																															3				
																																																															1				
																																																				1													
									2																																											3													
									2																																																									
			1						1																																											2											9	2		
									4																																																									
									5																											1																																	
									1																											8		4																												1		
																																				10		2																			1							2				
																																				6		2																										4		1		
																																				6		2																														
																																				16		1																1									3				
																																		4			6																															
																																				28		9																														
																								1												35		3																														
																																		1			34		2																														
																																				24		1																1													
																																																													1							
																																																													1							
																																																													3							
3	0	0	1	0	0	0	0	0	0	15	0	0	0	0	0	0	0	0	0	0	0	0	0	0	1	0	0	0	0	0	0	0	0	5	0	0	174	0	26	0	0	0	0	0	0	0	0	0	0	0	0	0	0	0	8	0	1	0	0	0	5	0	0	14	9	4	0	0

WEDNESDAY 16.12.15 P40 + 30d SCT results.

CW103
SCT

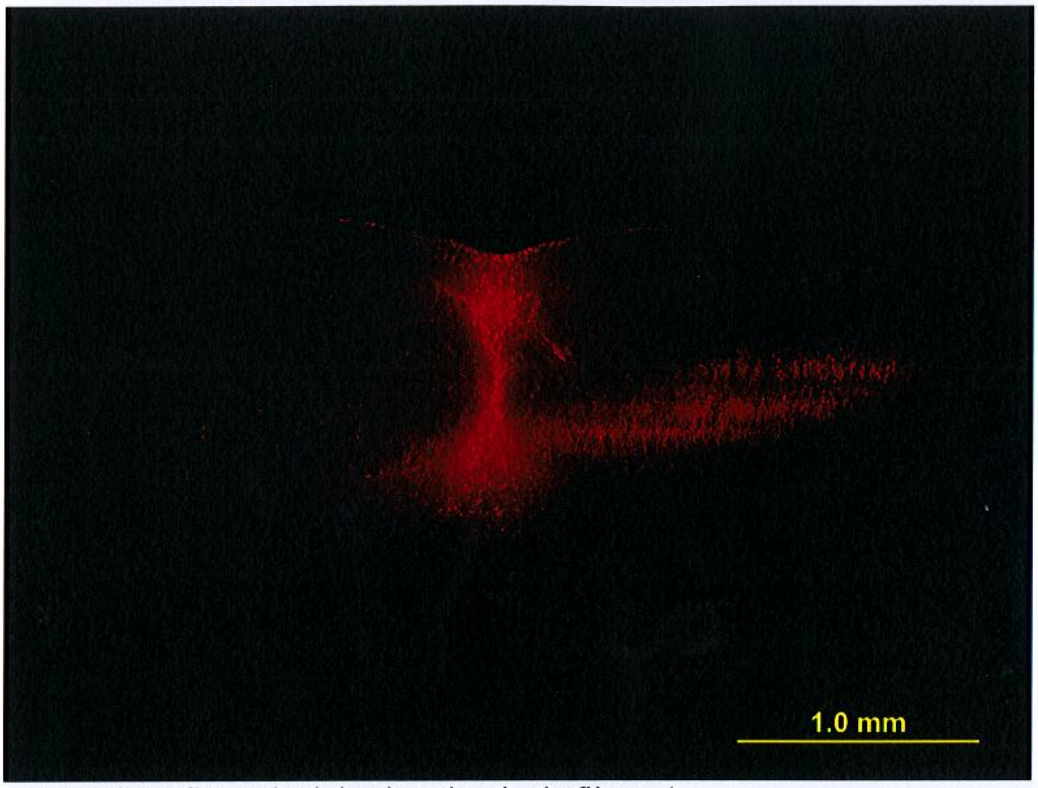

CW103 SCT fluroruby injection site single filter x4

given at P41 + 30d.

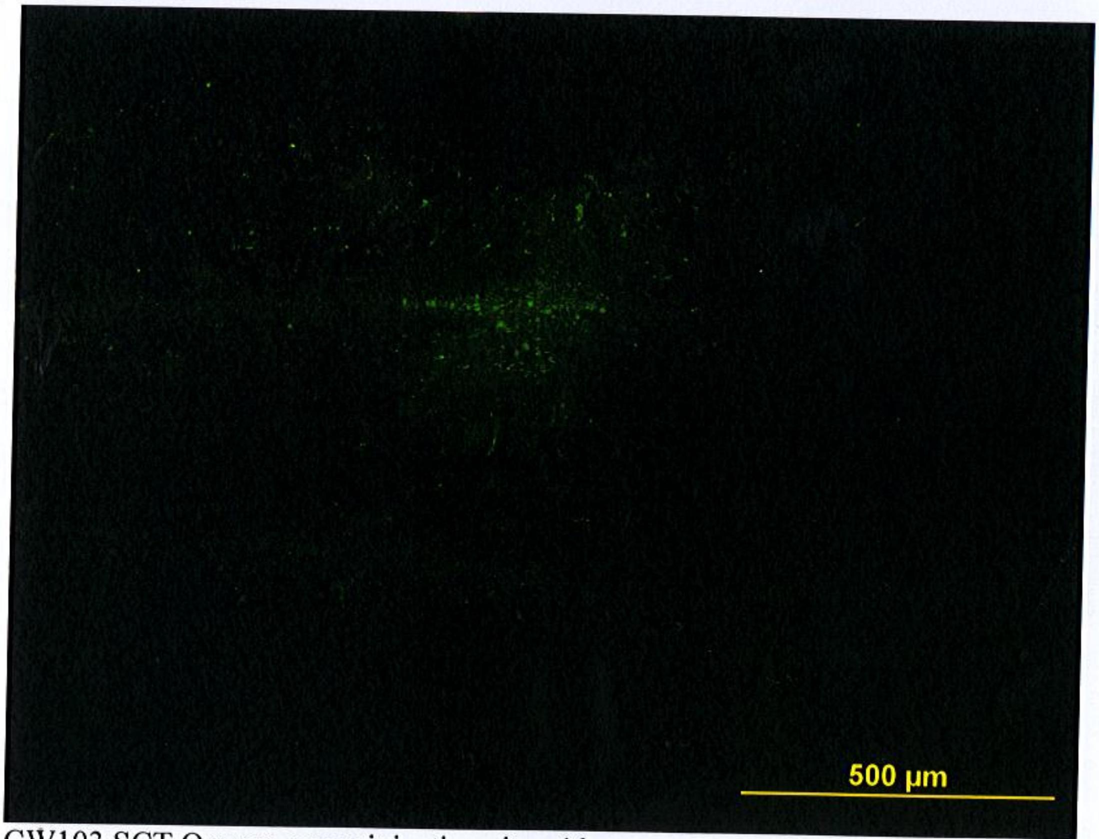

CW103 SCT Oregon green injection site x10

given at P41 at time of injury

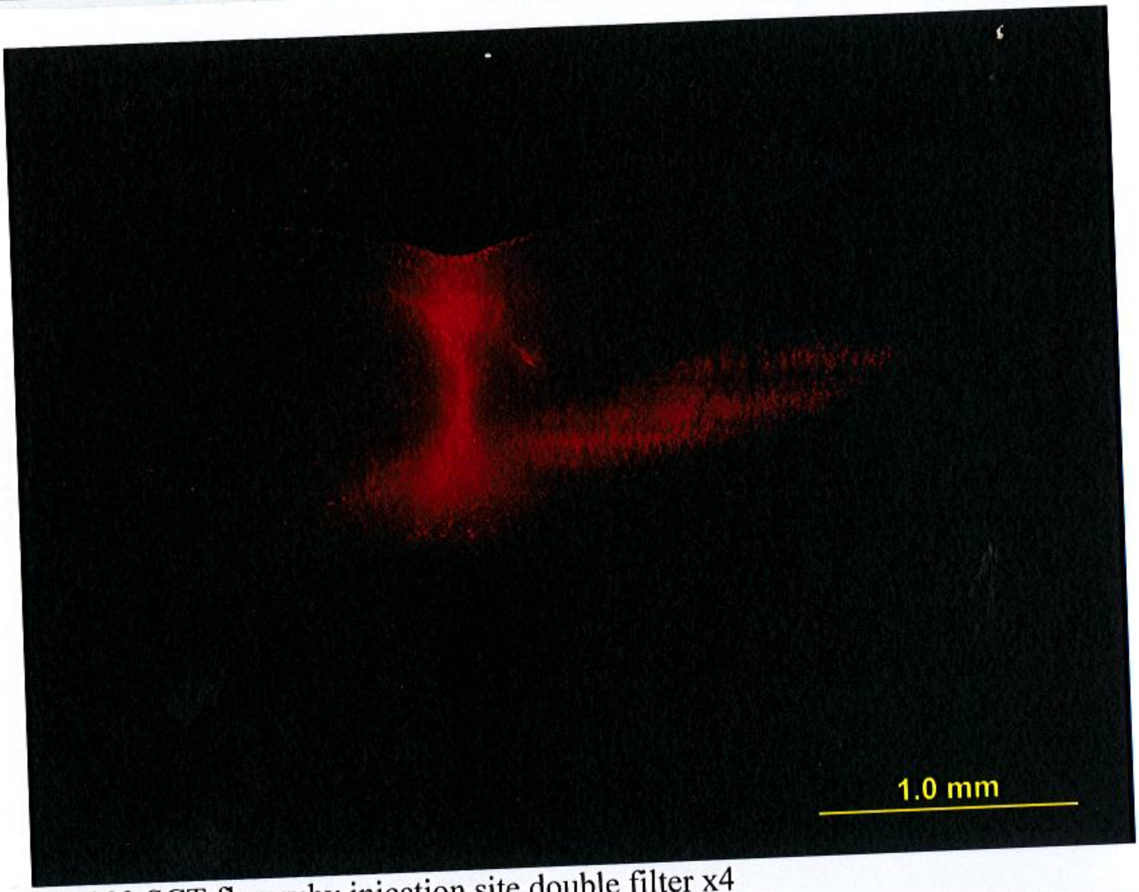

CW103 SCT fluroruby injection site double filter x4

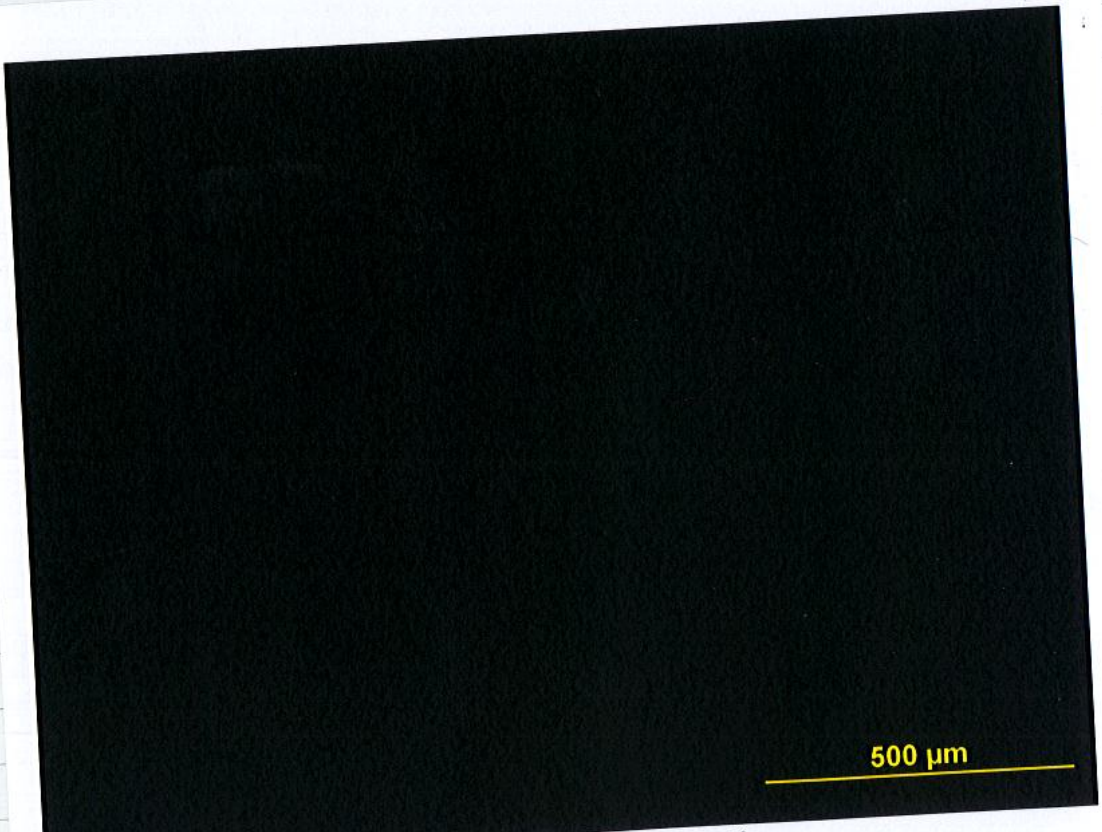

CW103 SCT most caudal end of rostral segment x10

no Fluroruby or oregon green positive cells/tissue above injury site.

CW96 SCT.

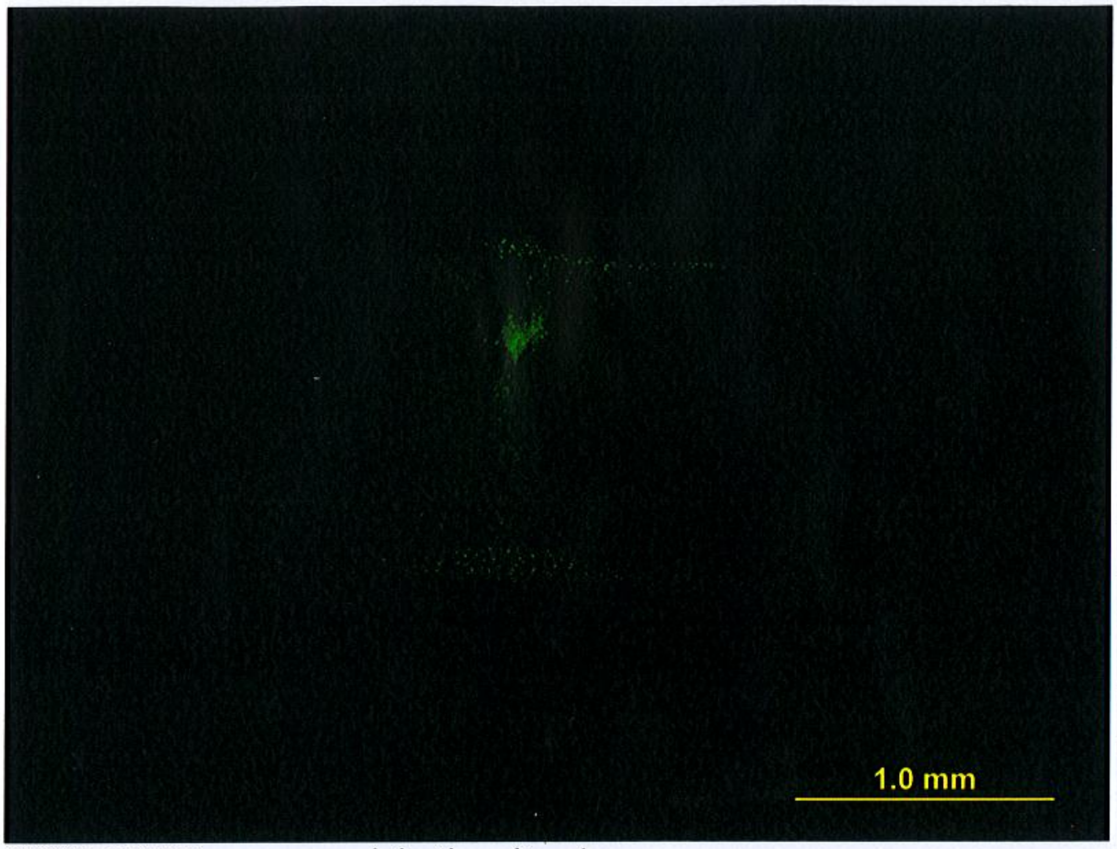

CW96 SCT Oregon green injection site x4

given at P41

CW96

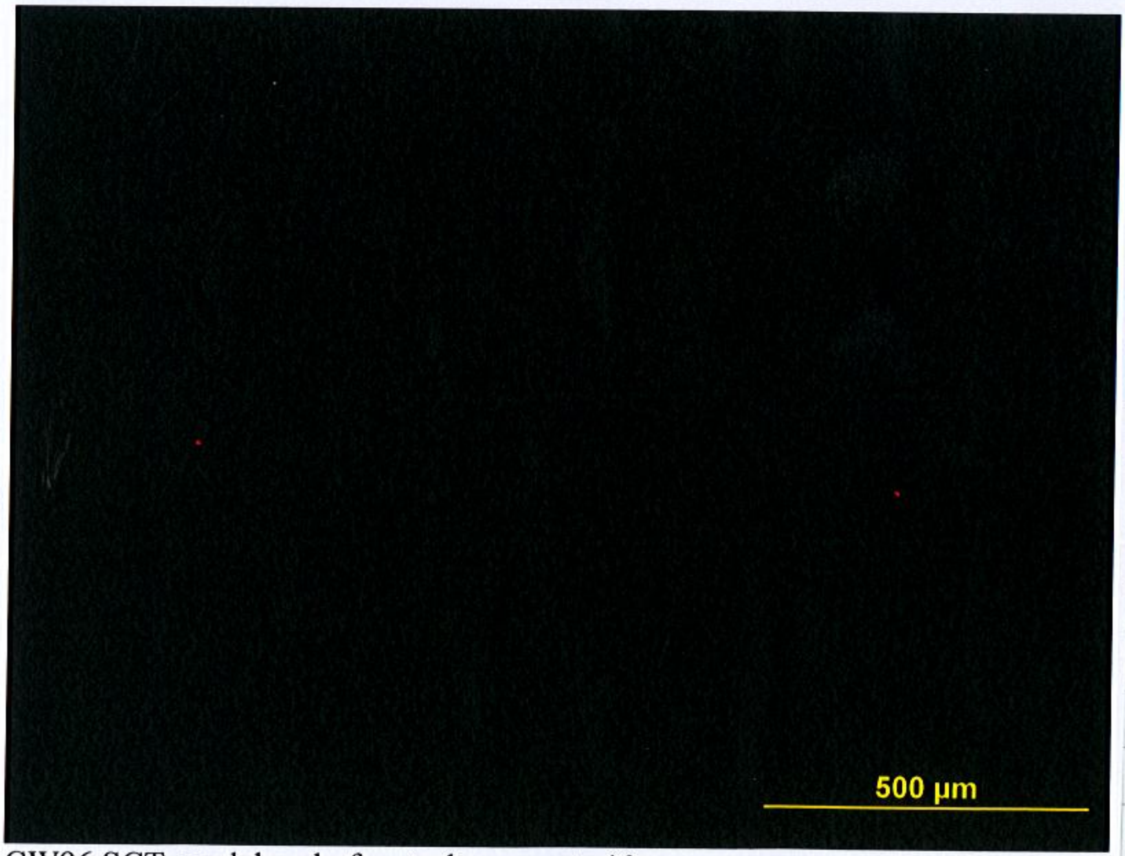

CW96 SCT caudal end of rostral segment x10

given at P41 + 30d.

CW98 SCT

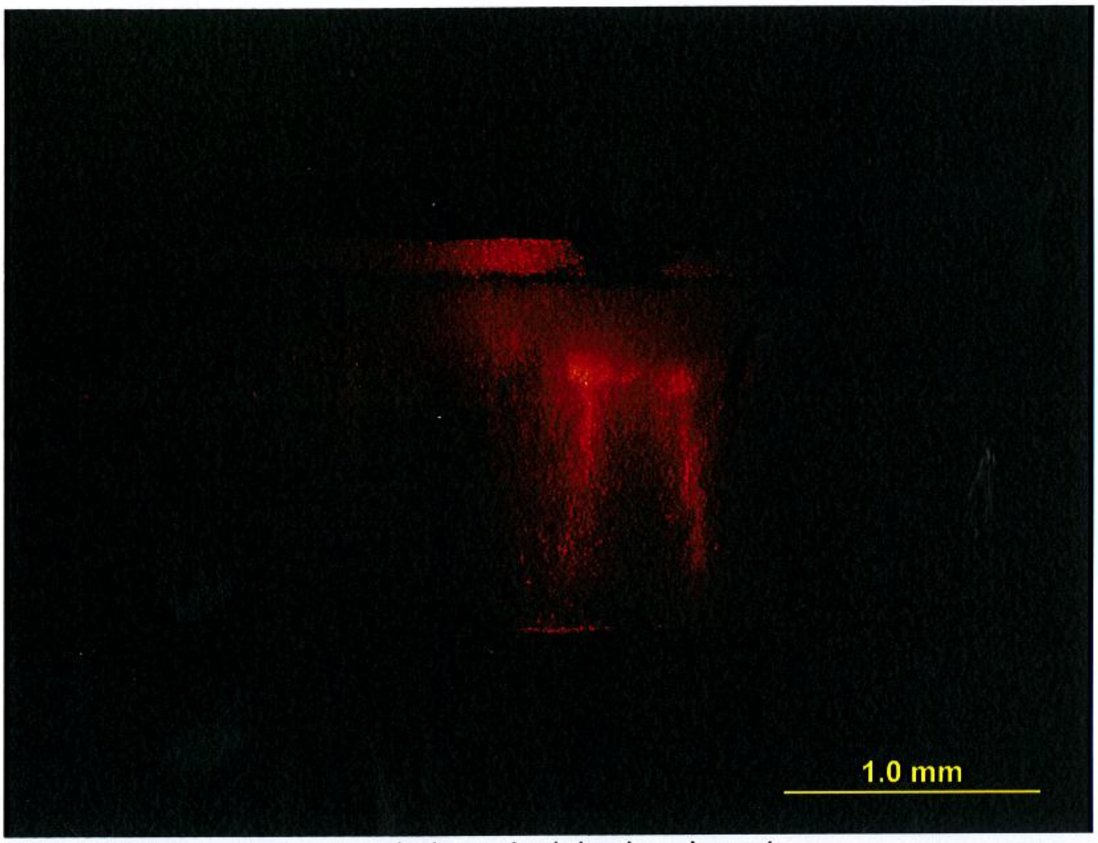

CW98 SCT Oregon green and Fluroruby injection sites x4

double filter

CW98 SC

~~blood vessels~~ ←

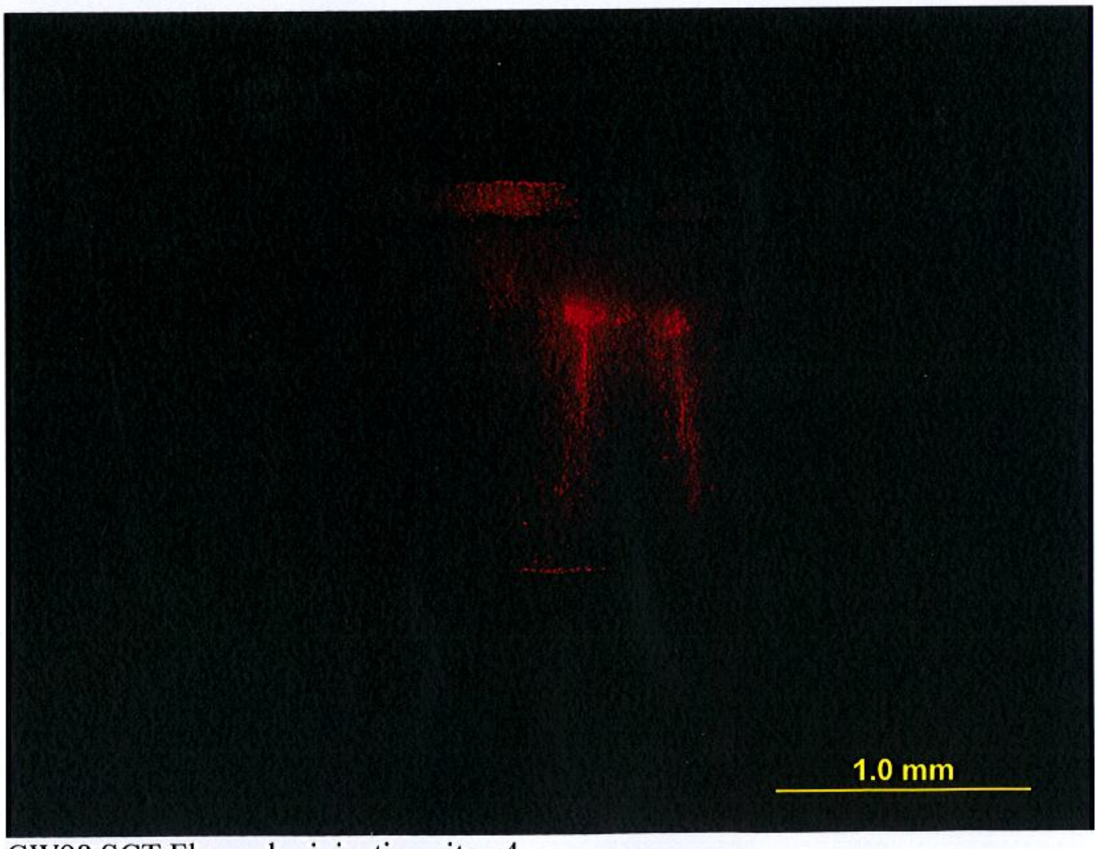

~~→ blood vessel positive for Fluoro~~

CW98 SCT Fluroruby injection site x4

~~above injury site~~

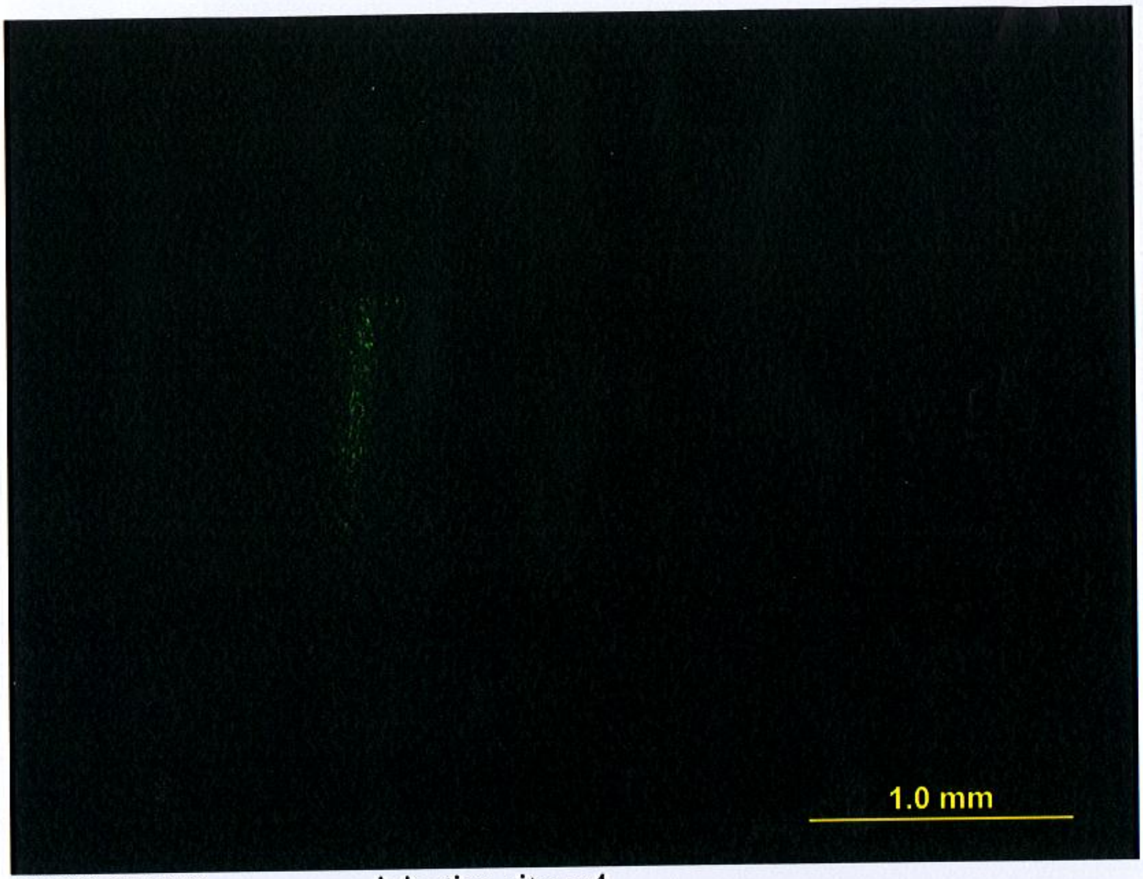

CW98 SCT Oregon green injection sites x4

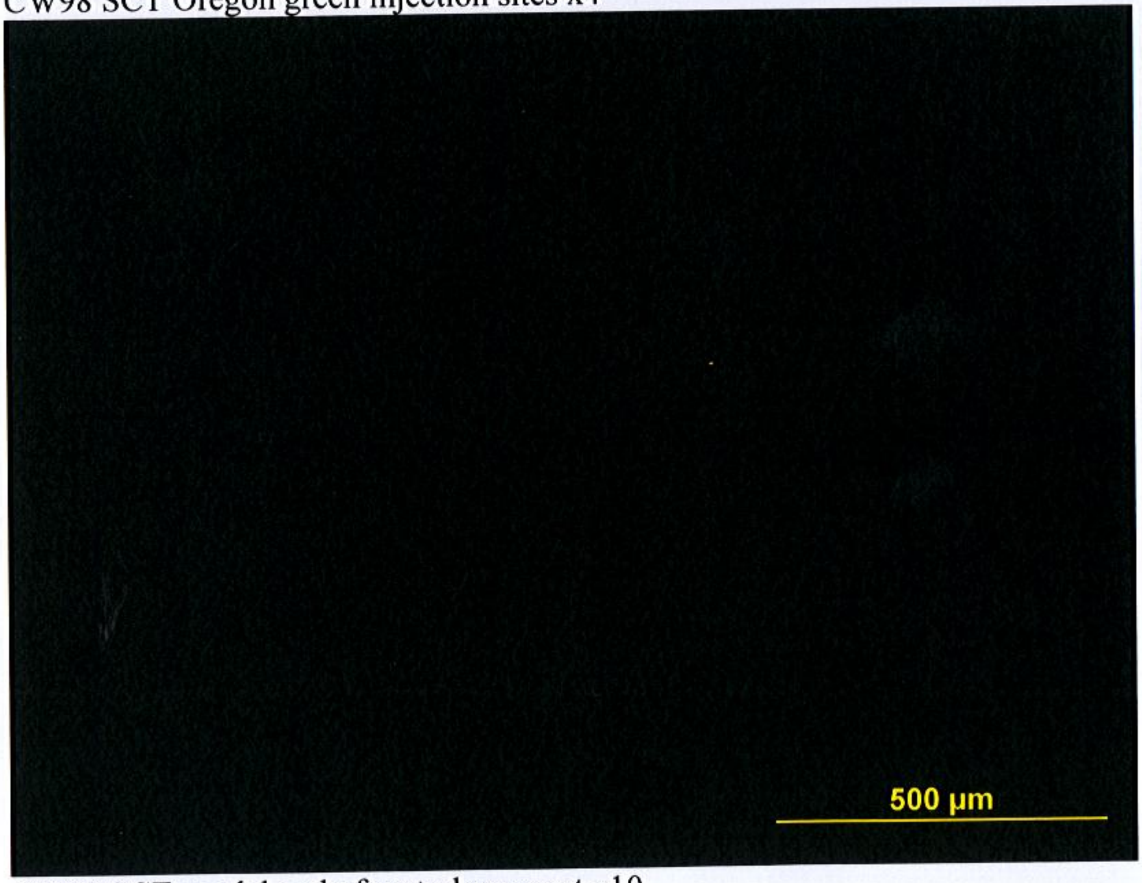

CW98 SCT caudal end of rostral segment x10

not Fluororuby or oregon green positive cells/tissue above injury site

CW104 control

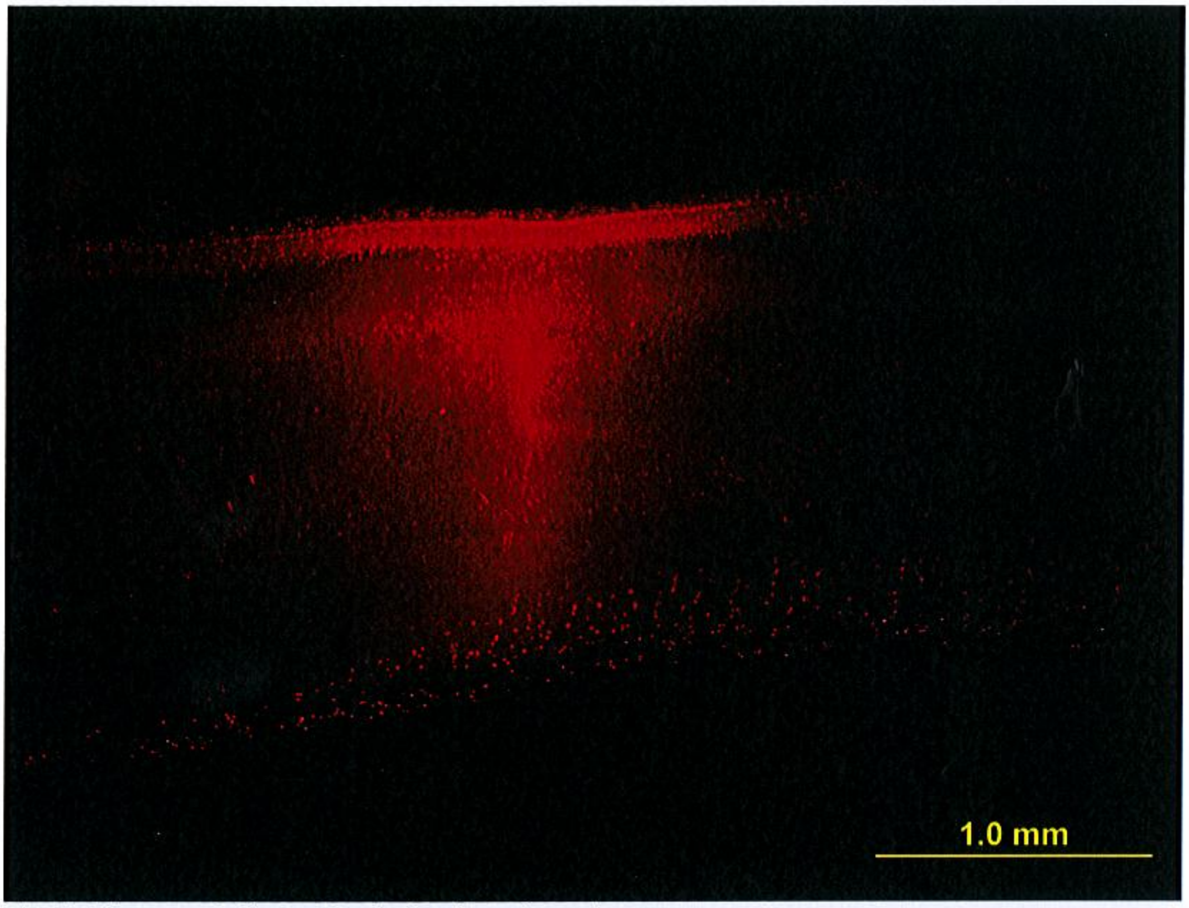

CW104 control fluroruby injection site x4

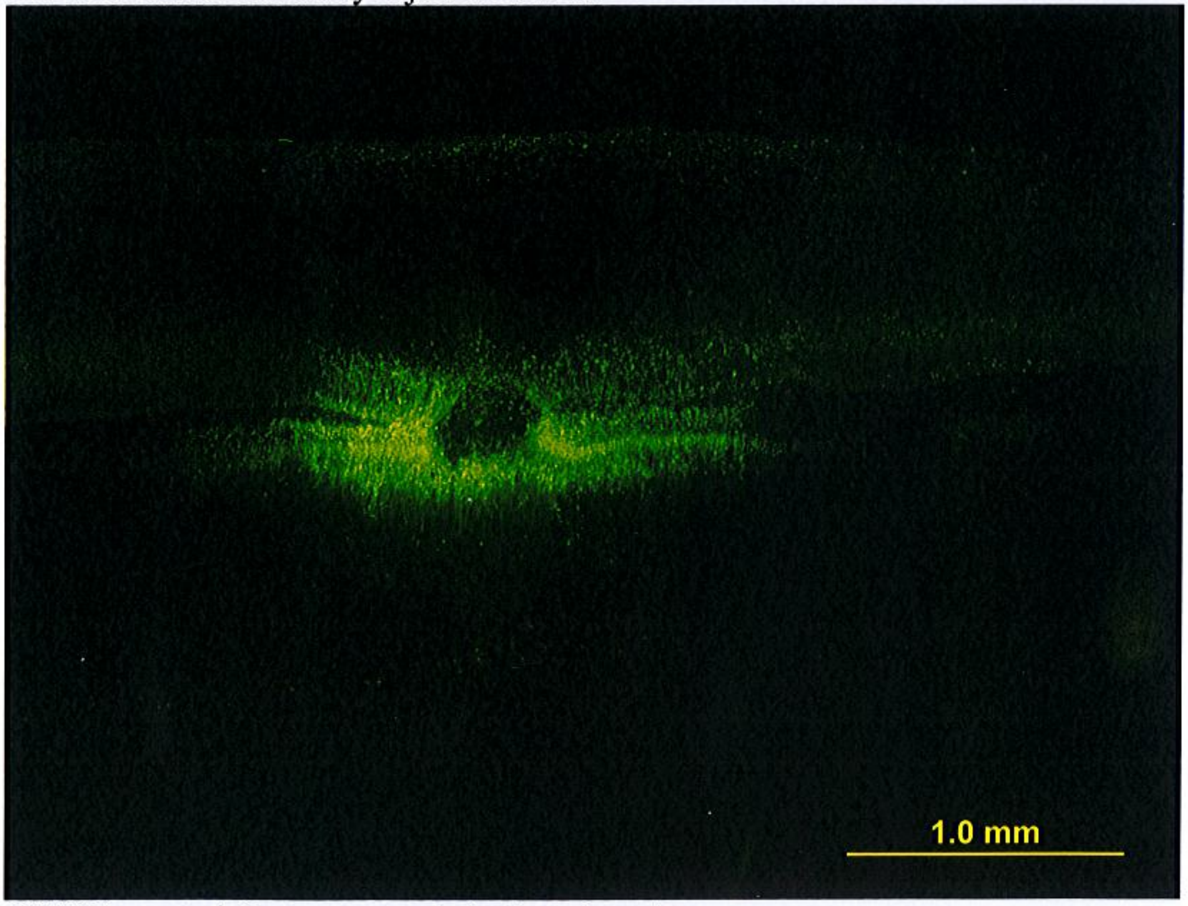

CW104 control Oregon green injection site x4

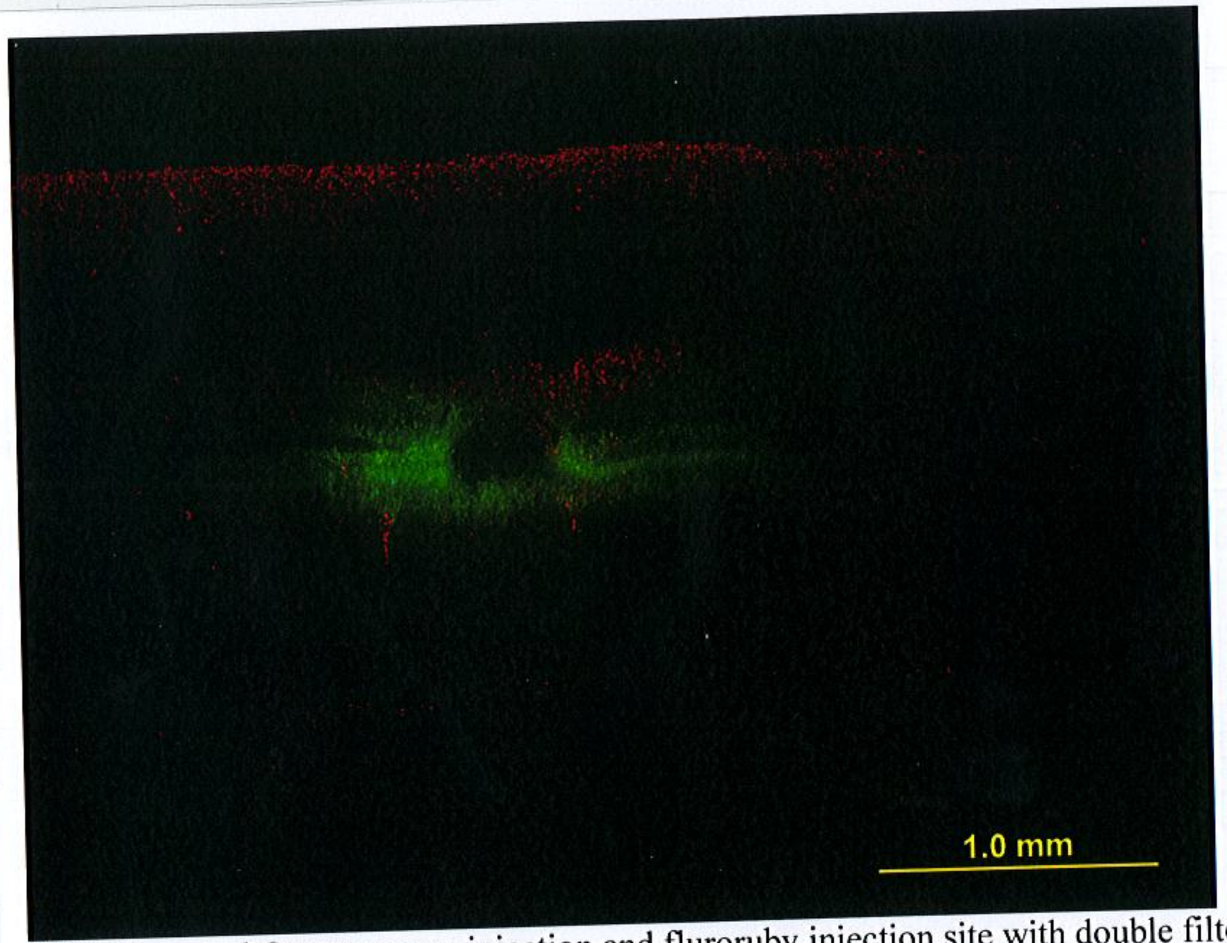

CW104 control Oregon green injection and fluroruby injection site with double filter x4

~~[illegible]~~ OG injections given at P40 and
F given at P70 days

CW104

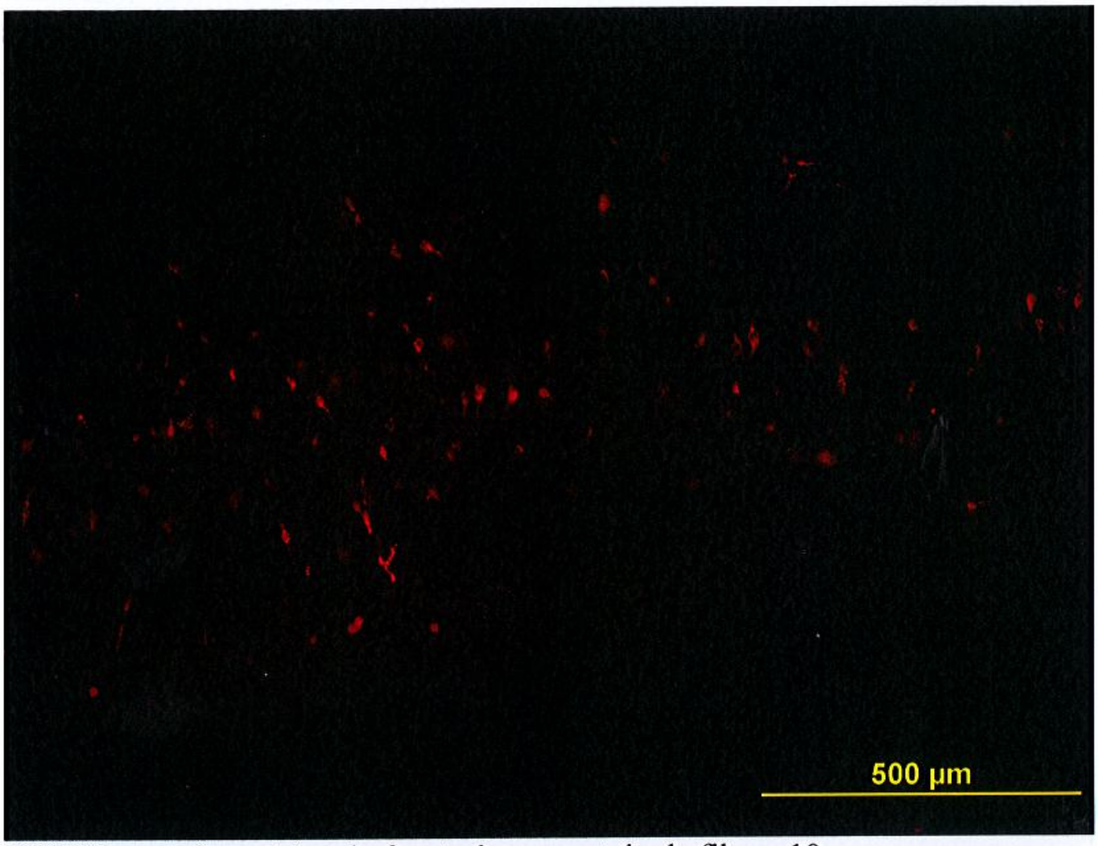

CW104 control caudal end of rostral segment single filter x10

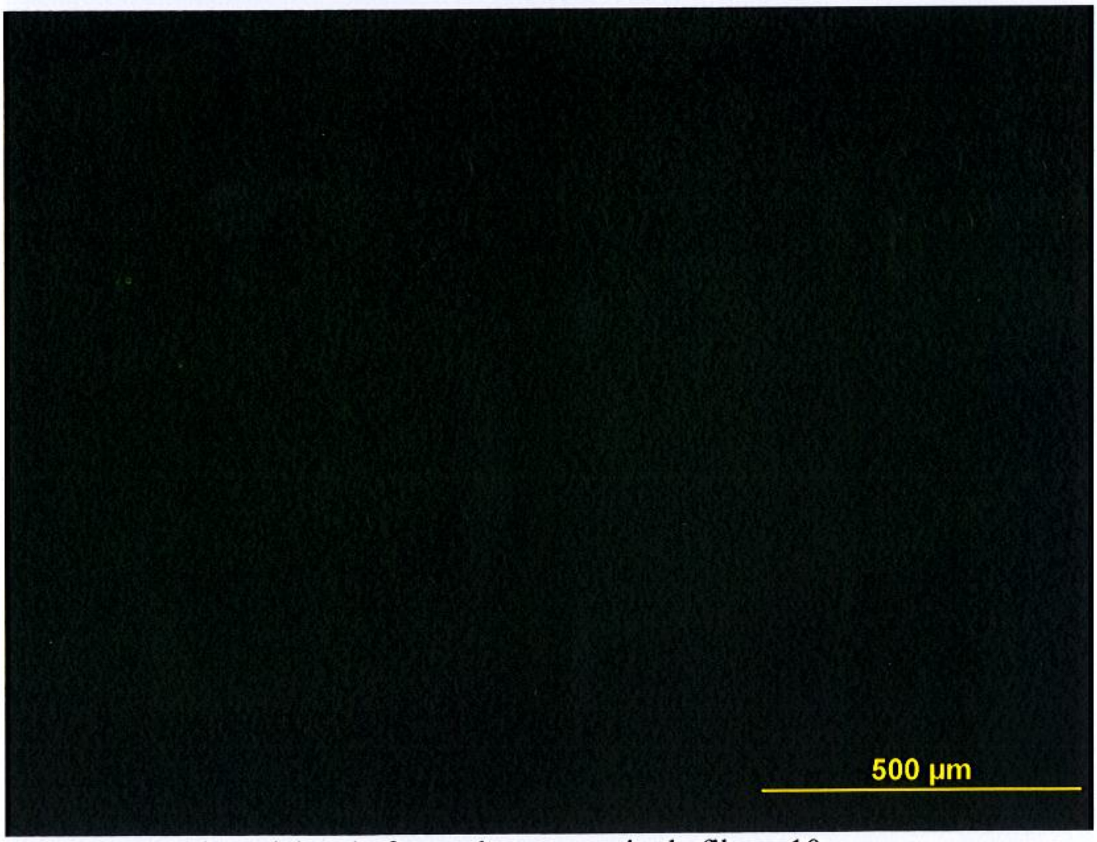

CW104 control caudal end of rostral segment single filter x10

DA and F positive cells/tissue above injection sites in rostral segment throughout cord

CW107 control

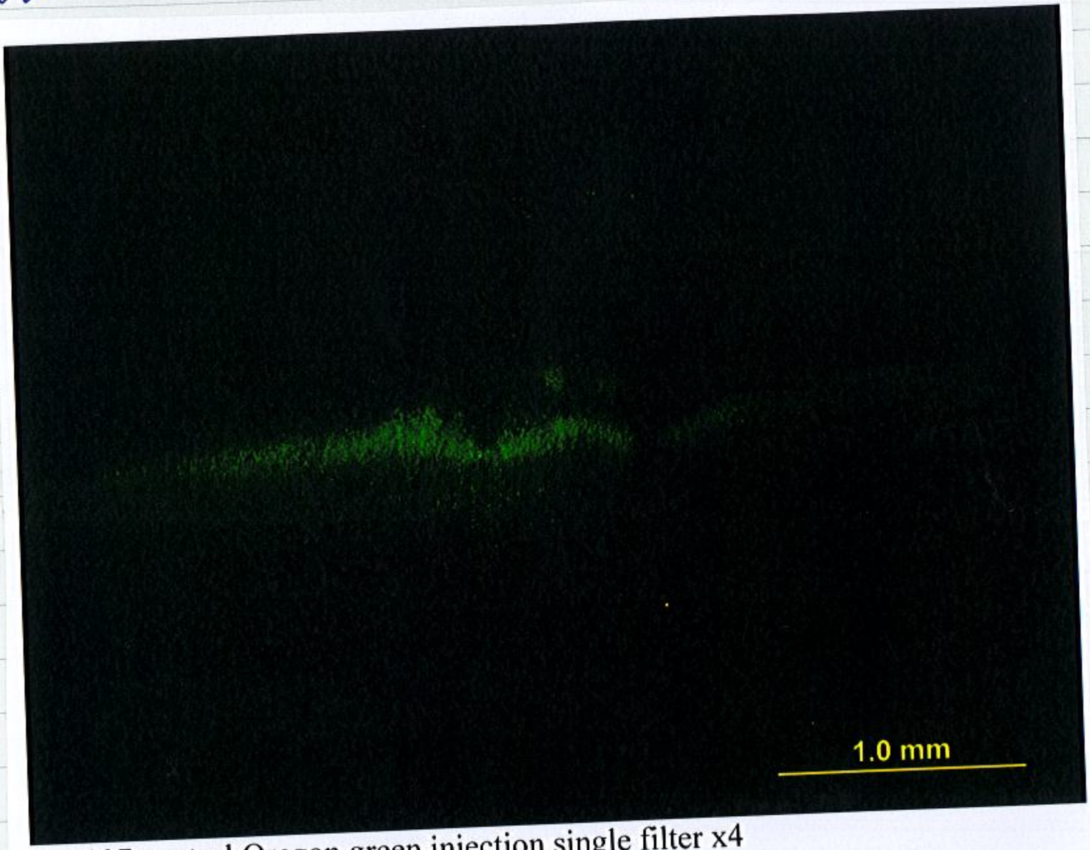

CW107 control Oregon green injection single filter x4

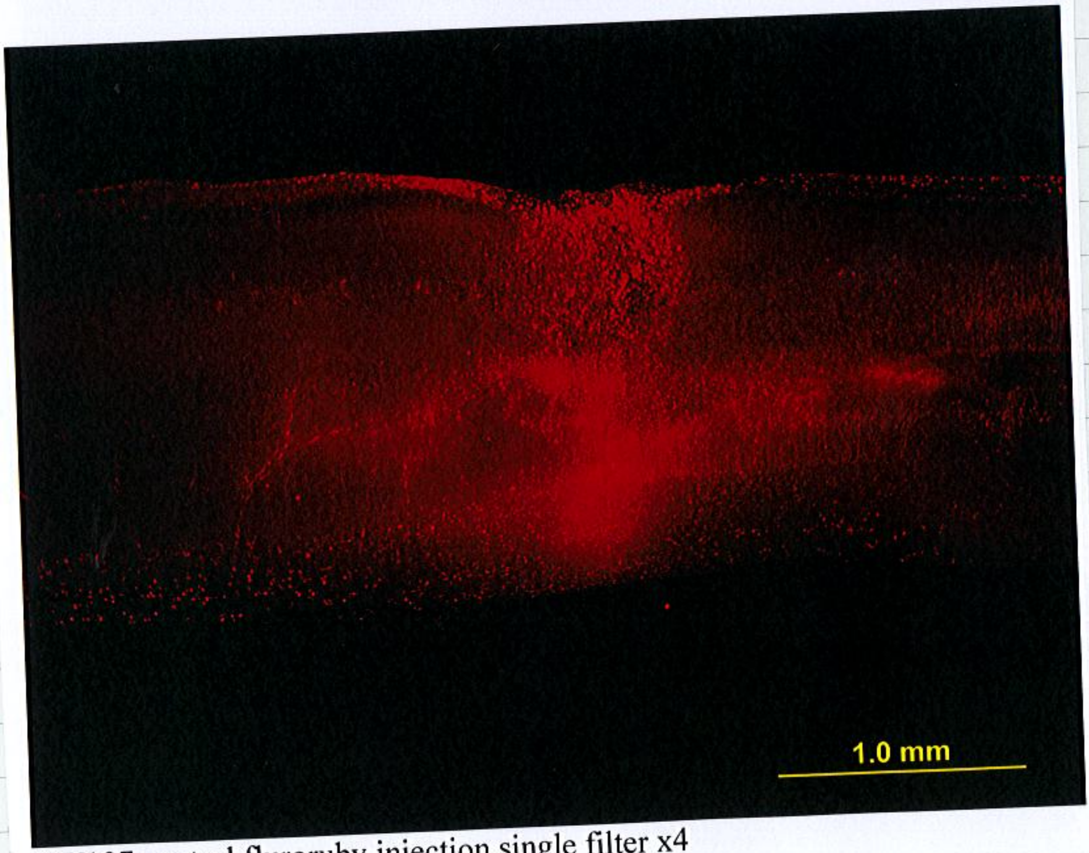

CW107 control fluroruby injection single filter x4

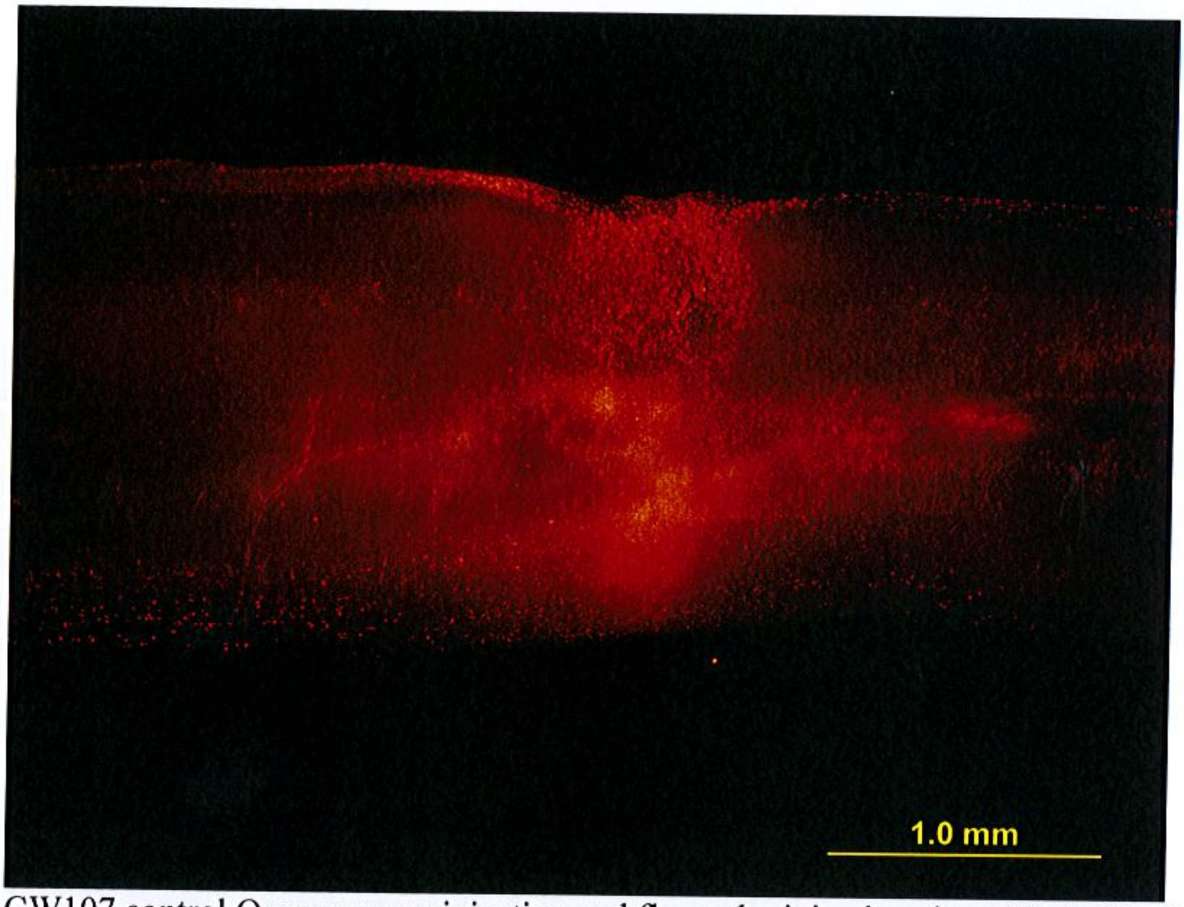

CW107 control Oregon green injection and fluroruby injection site with double filter x4

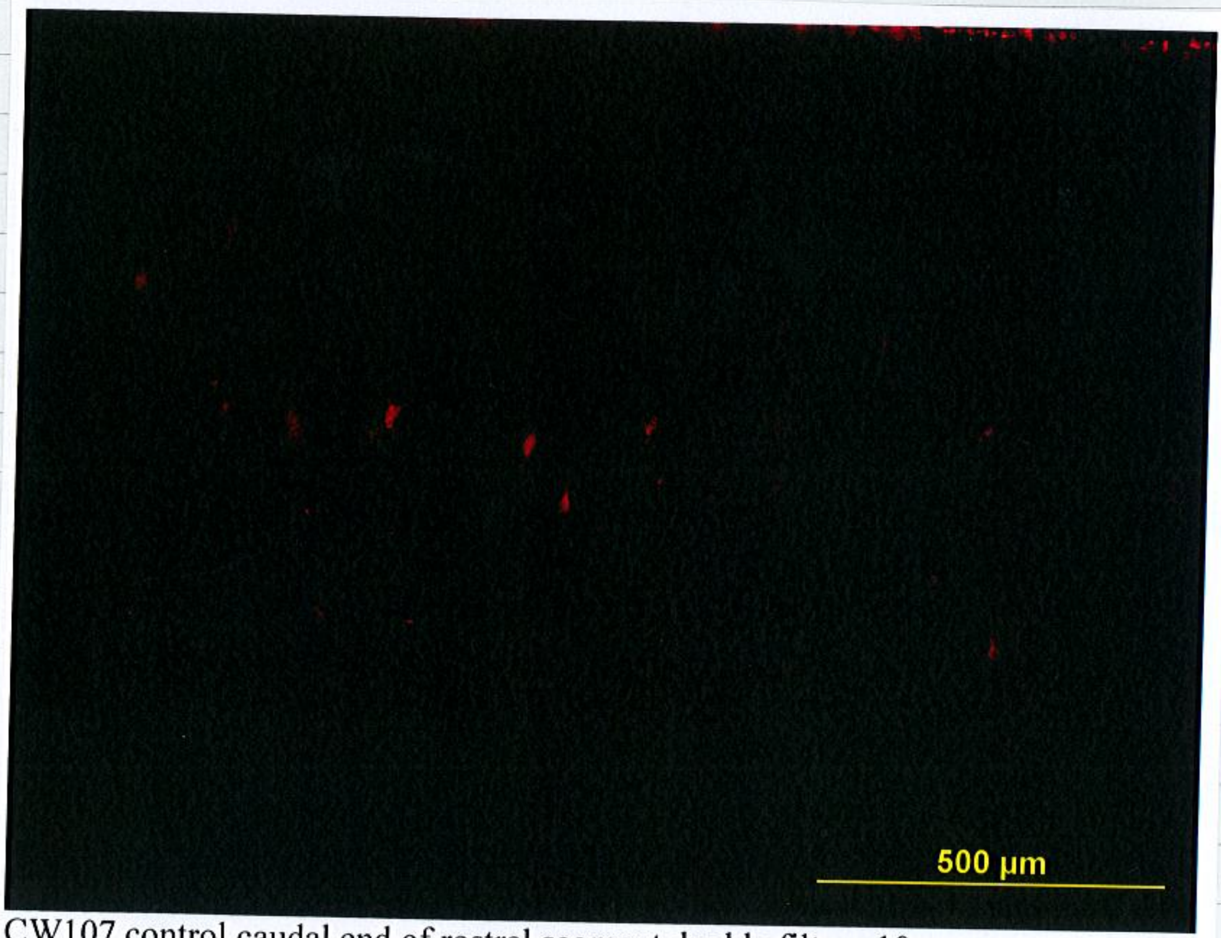

CW107 control caudal end of rostral segment double filter x10

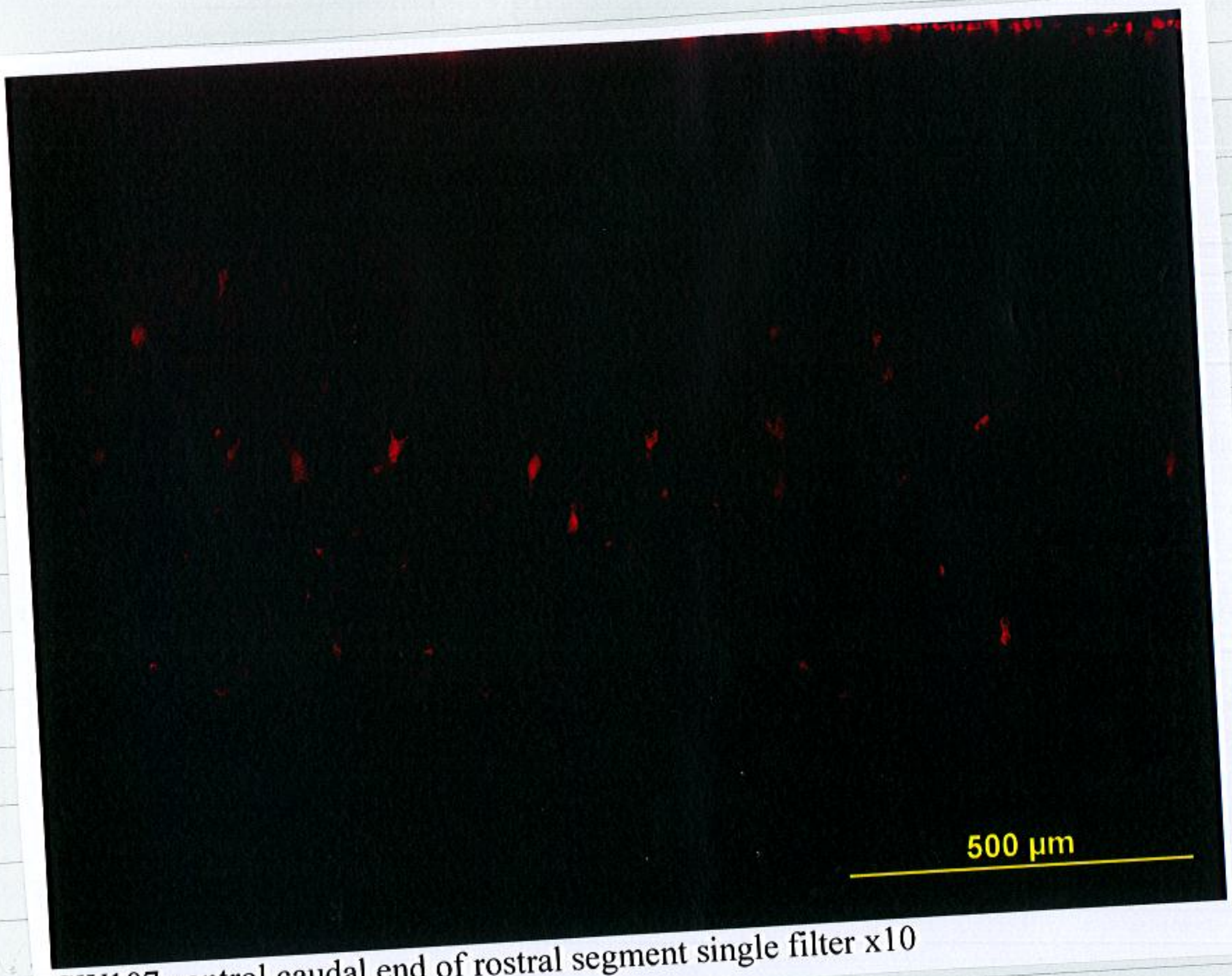

CW107 control caudal end of rostral segment single filter x10

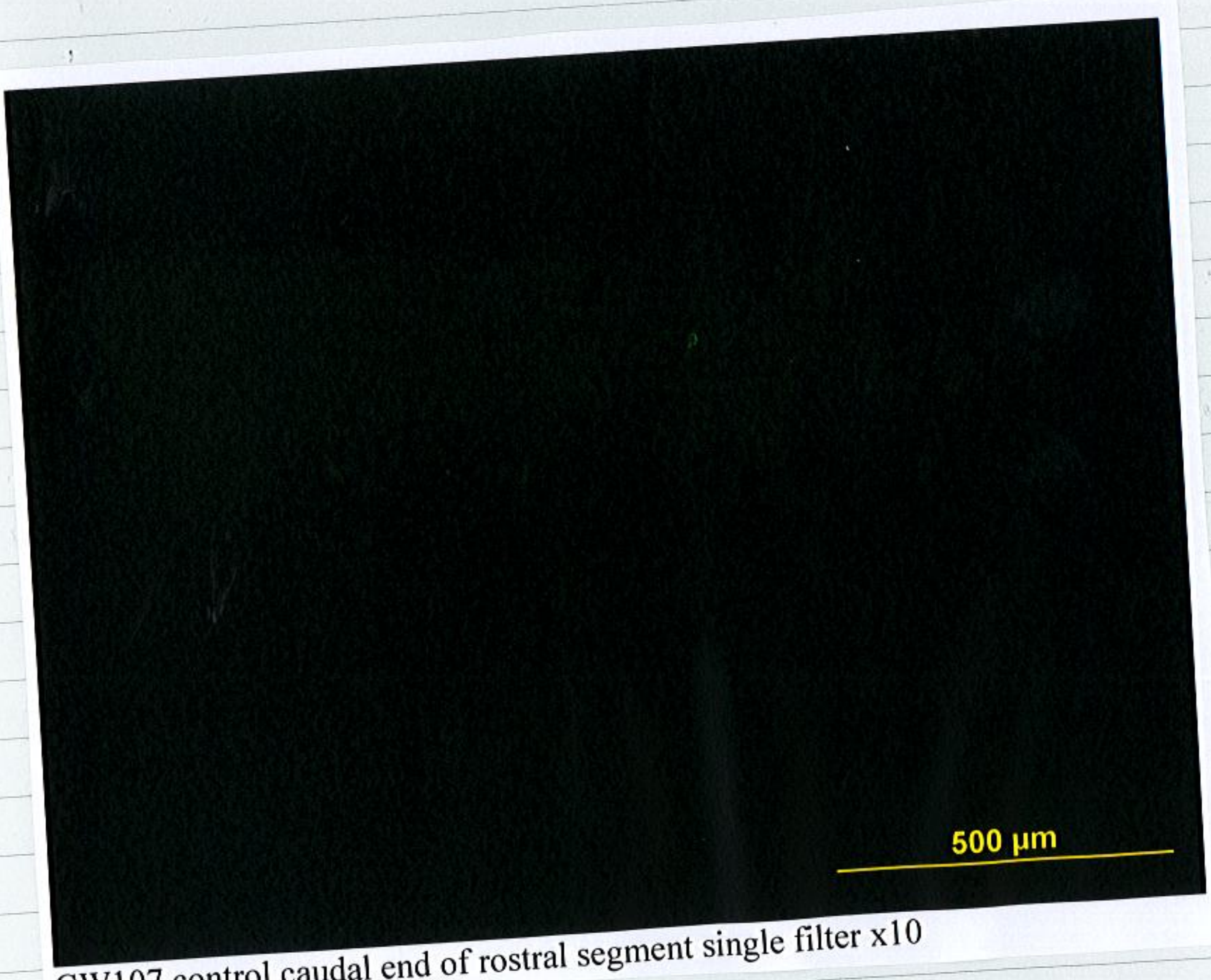

CW107 control caudal end of rostral segment single filter x10

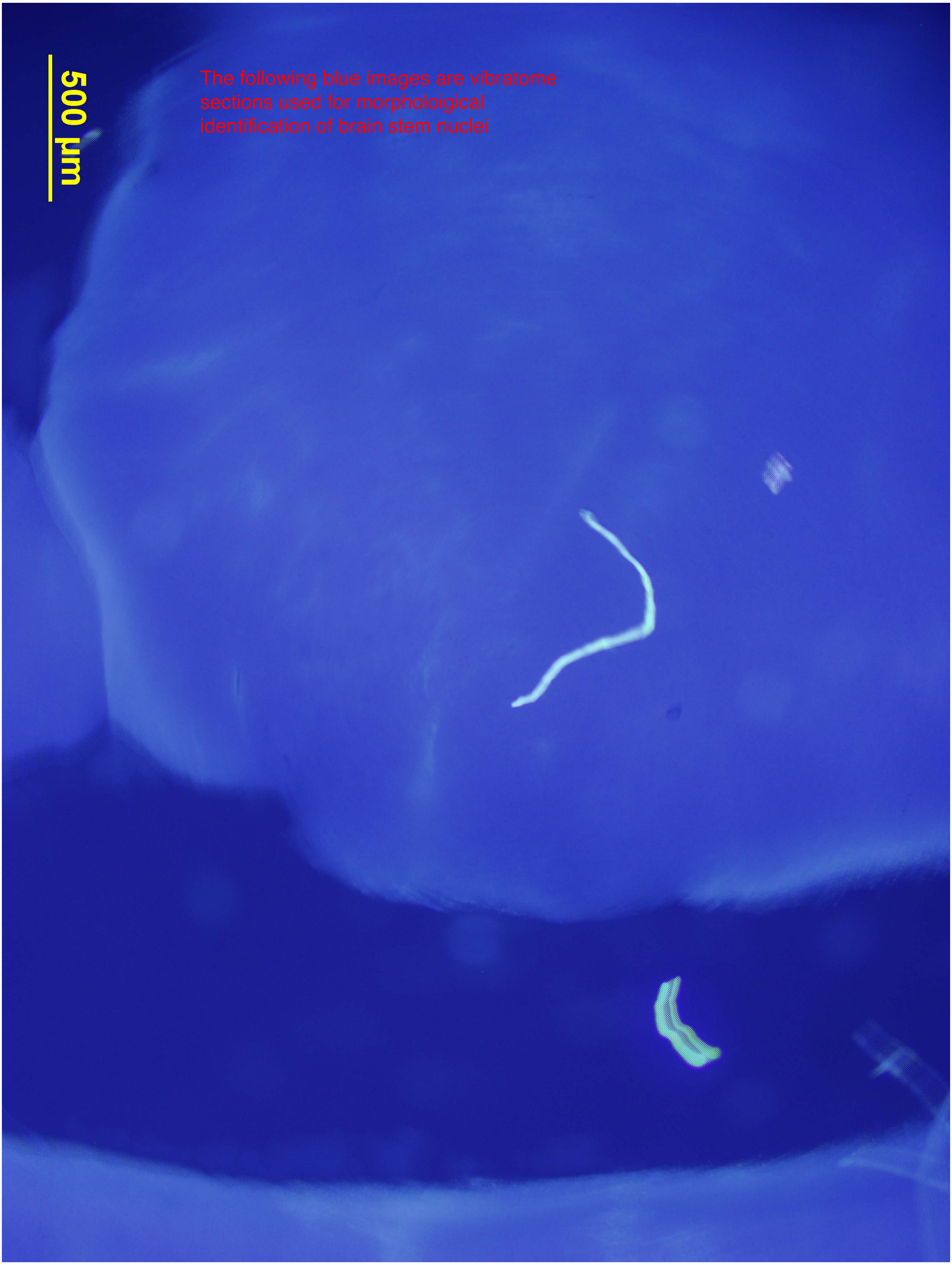
500 µm
The following blue images are vibratome
sections used for morpholoigical
identification of brain stem nuclei

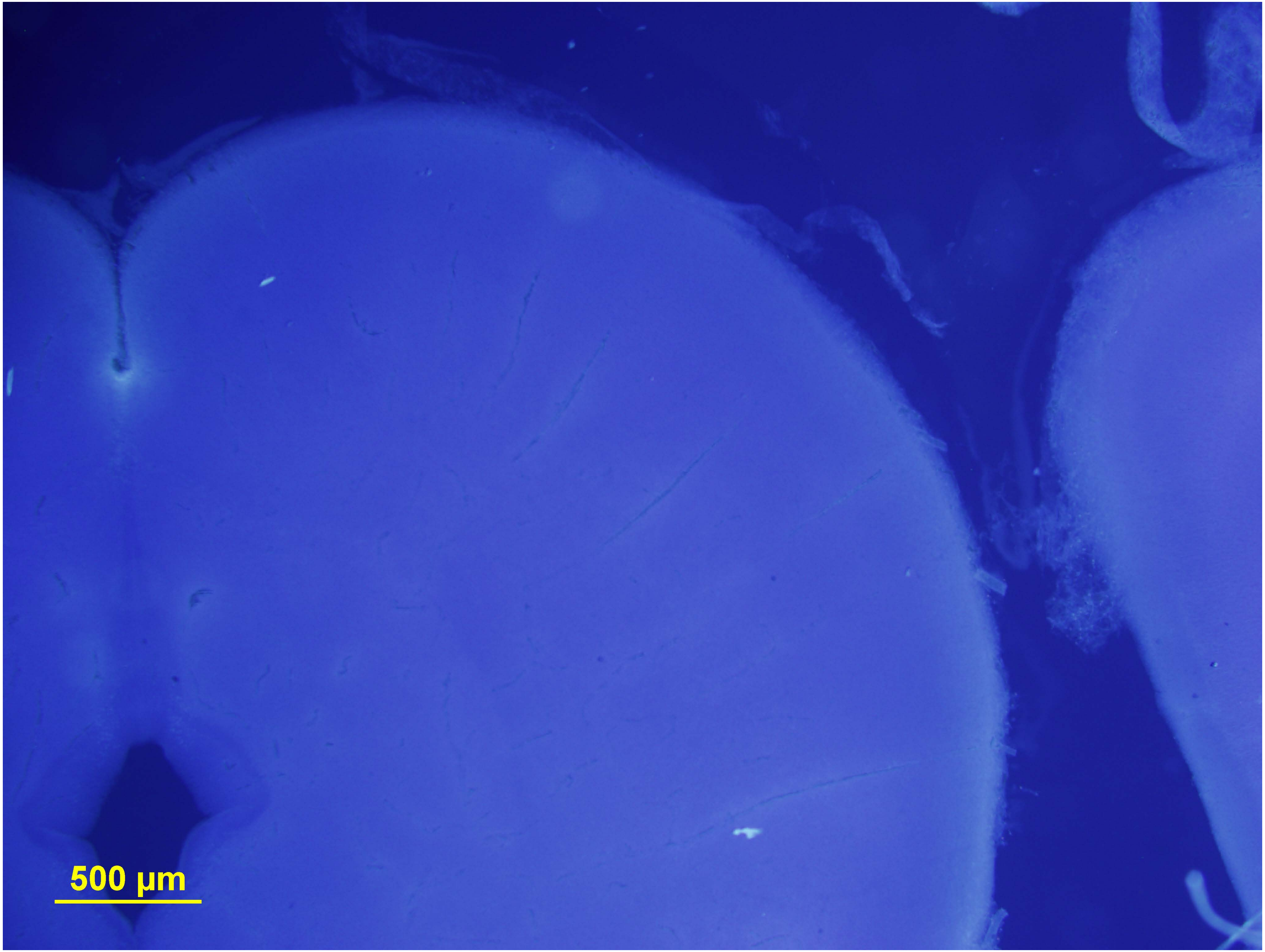
500 µm

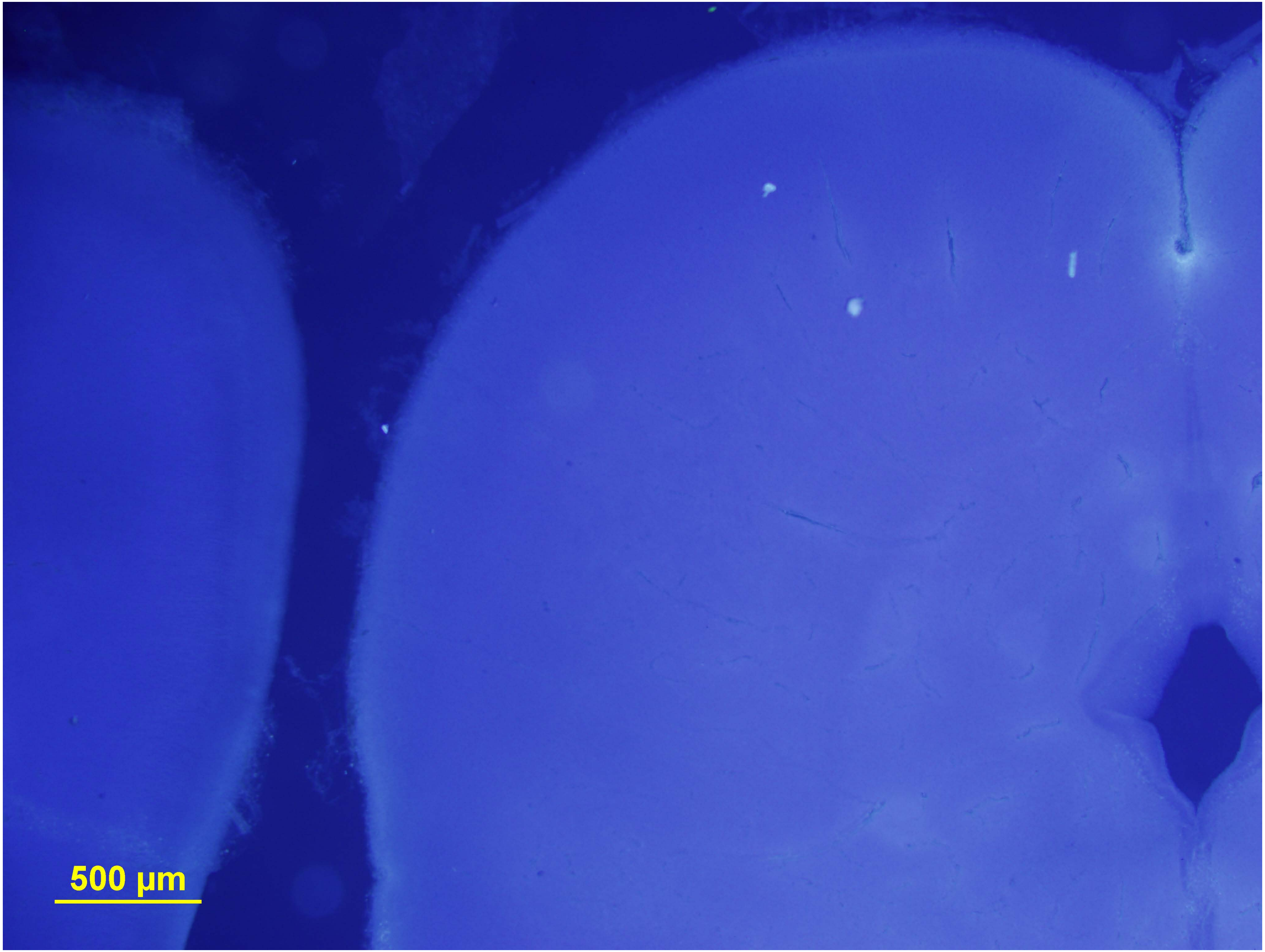
500 µm

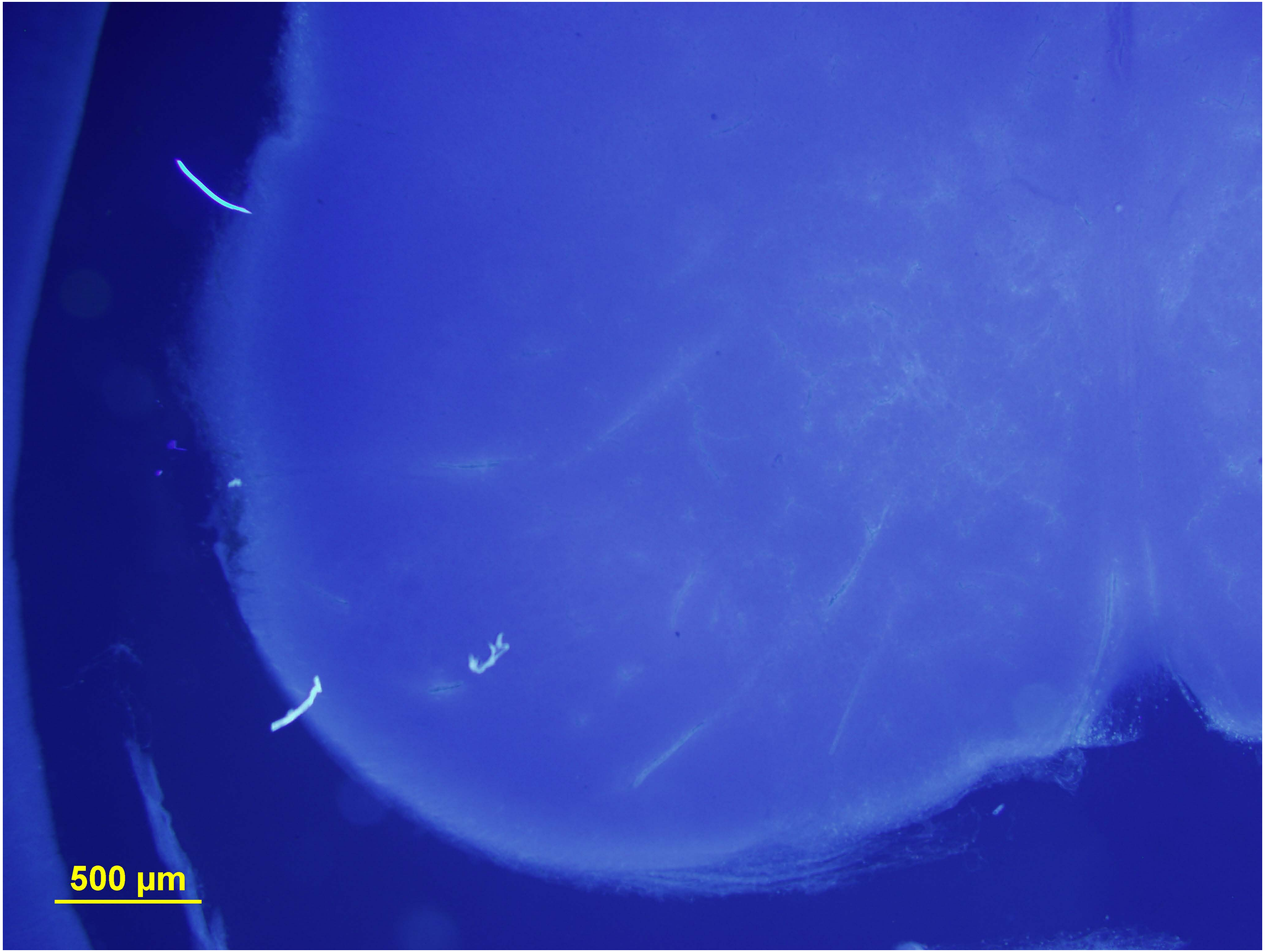
500 µm

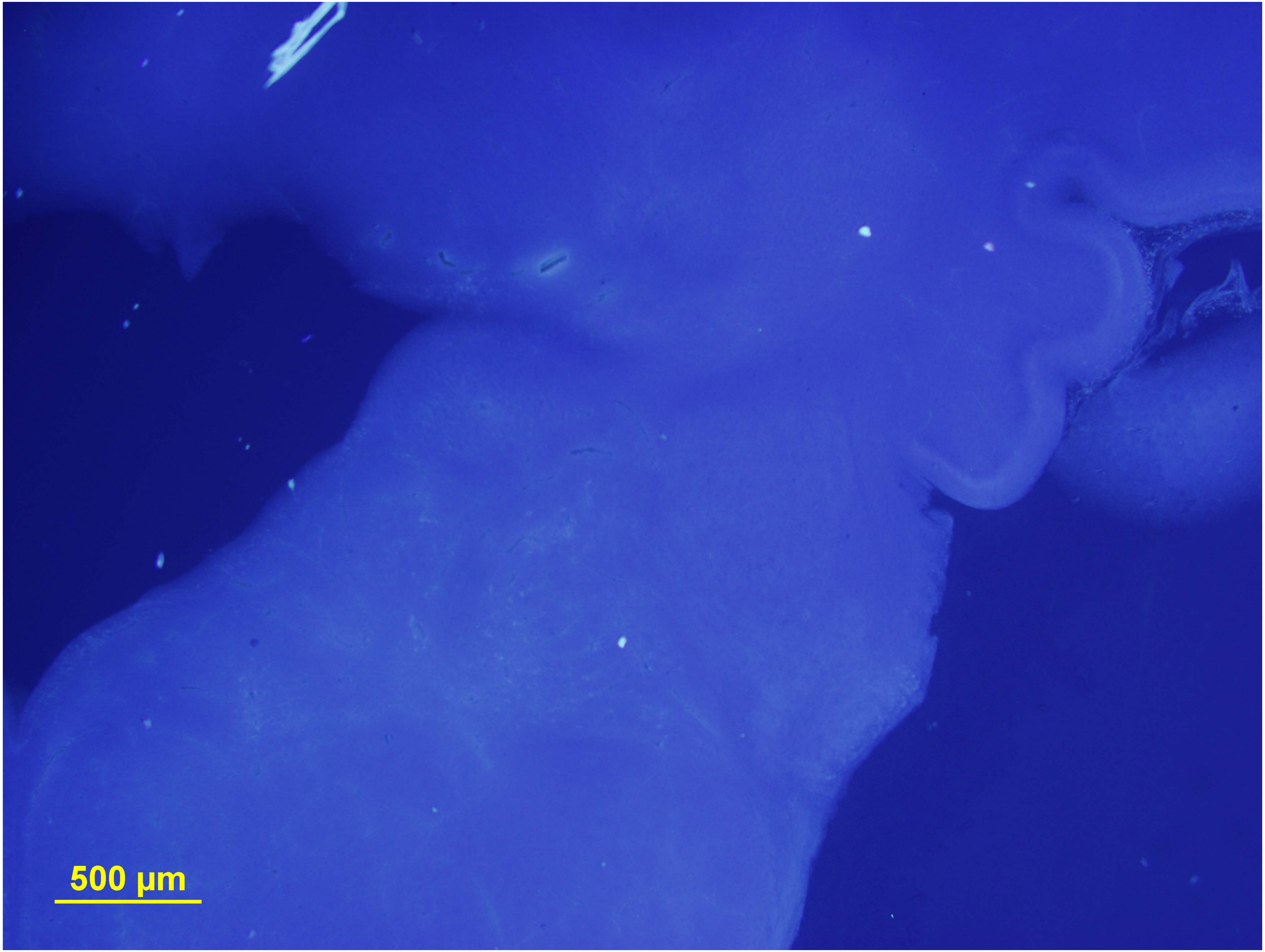
500 µm

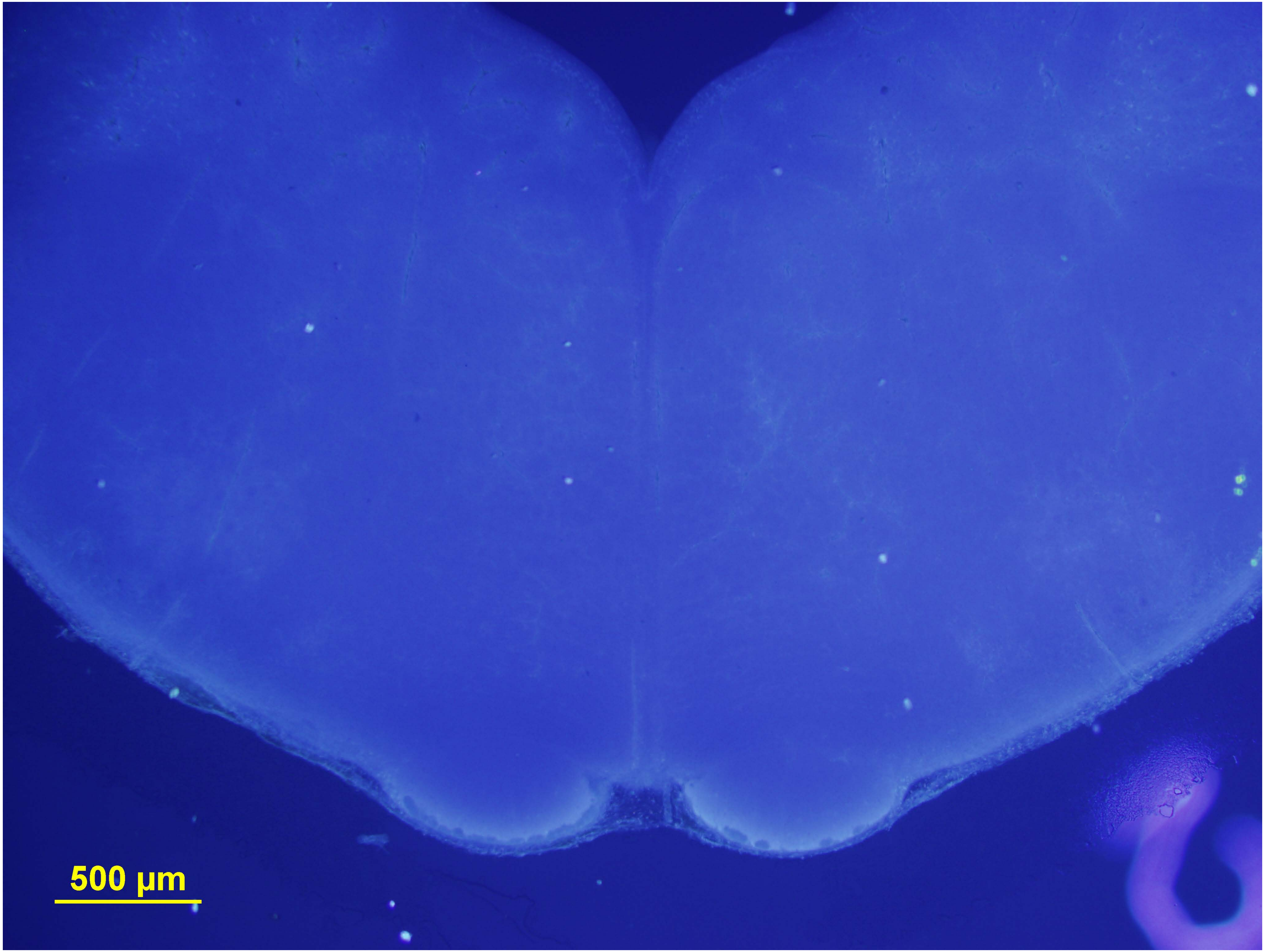
500 µm

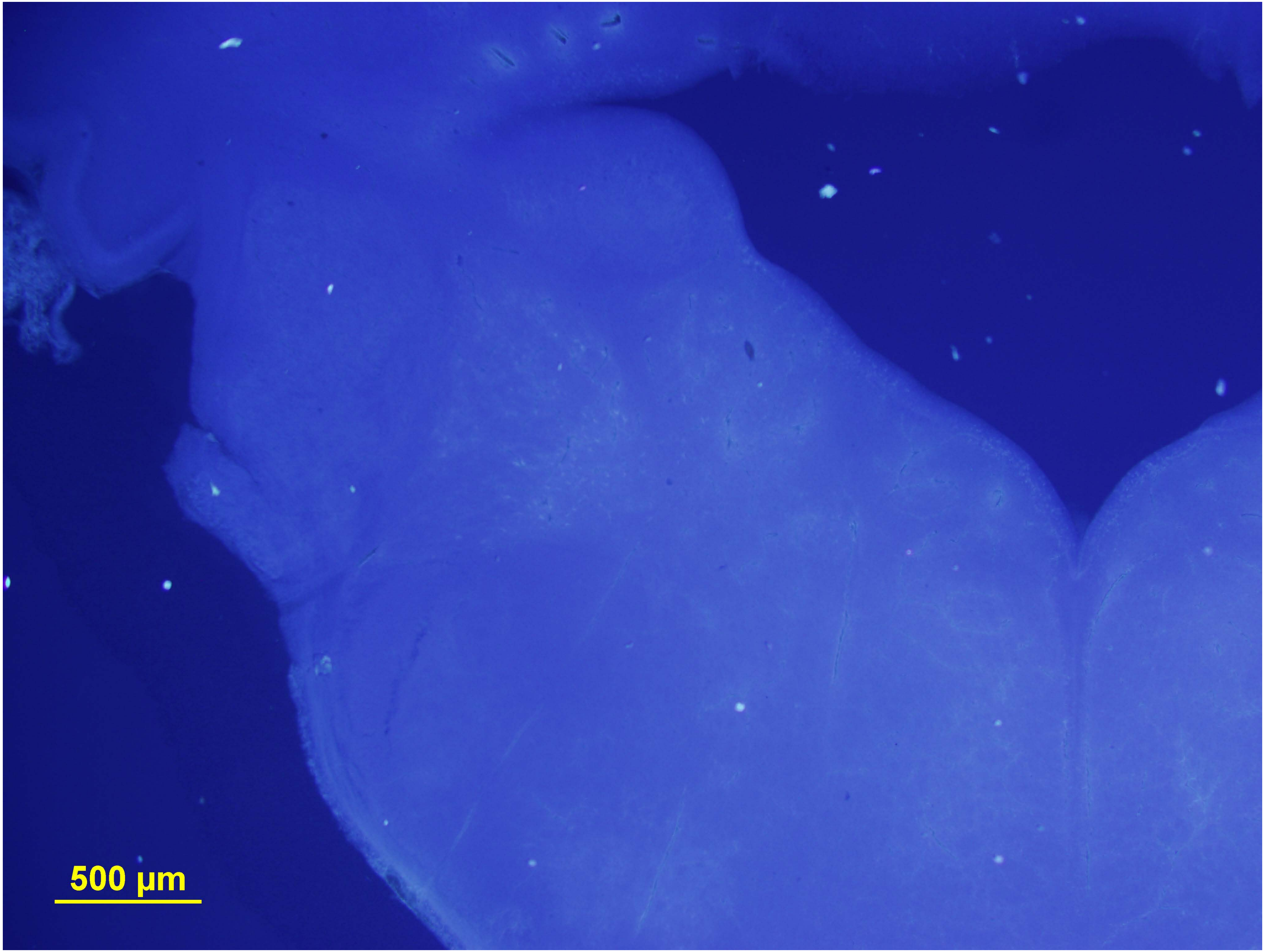
500 µm

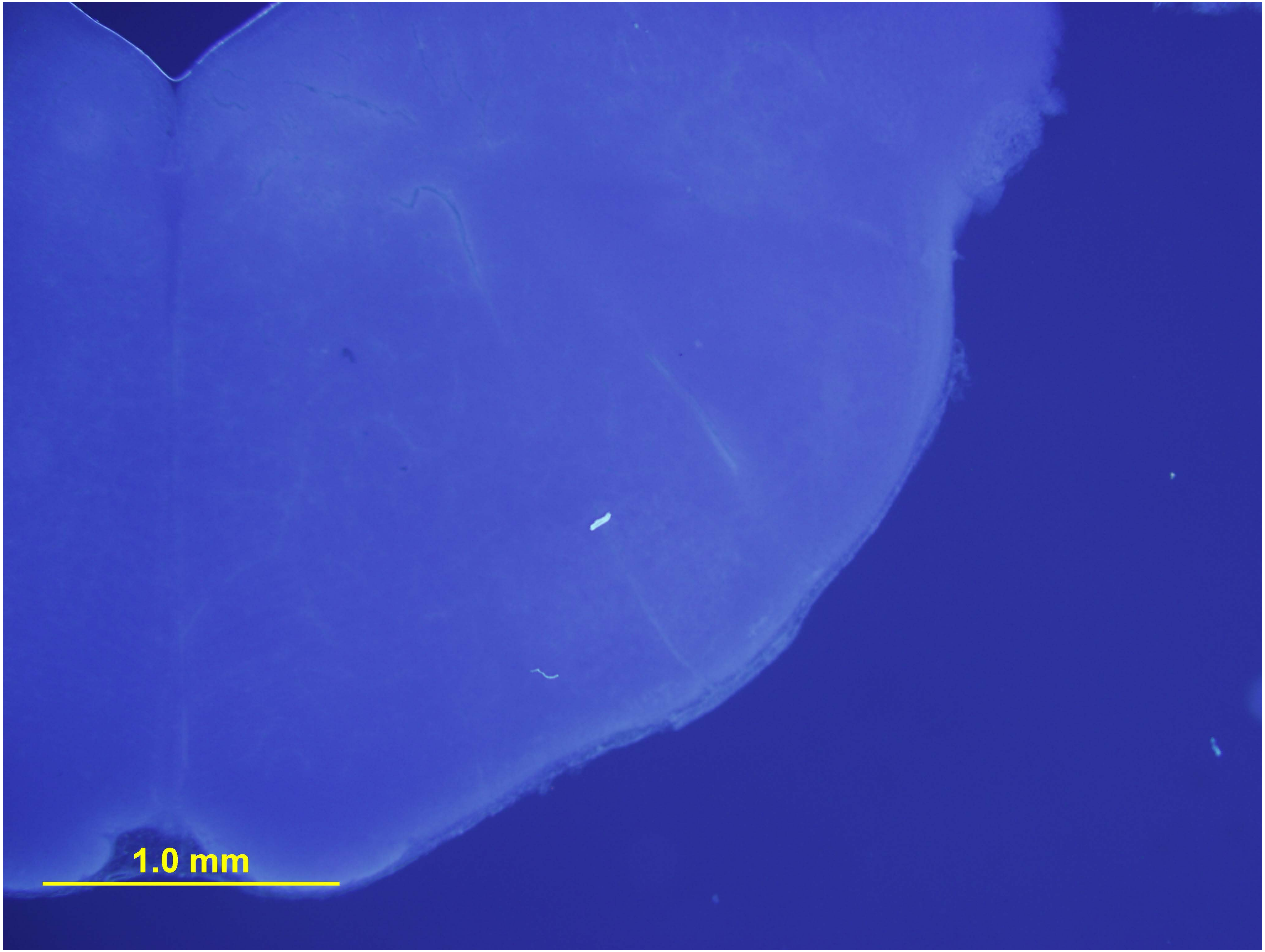
1.0 mm

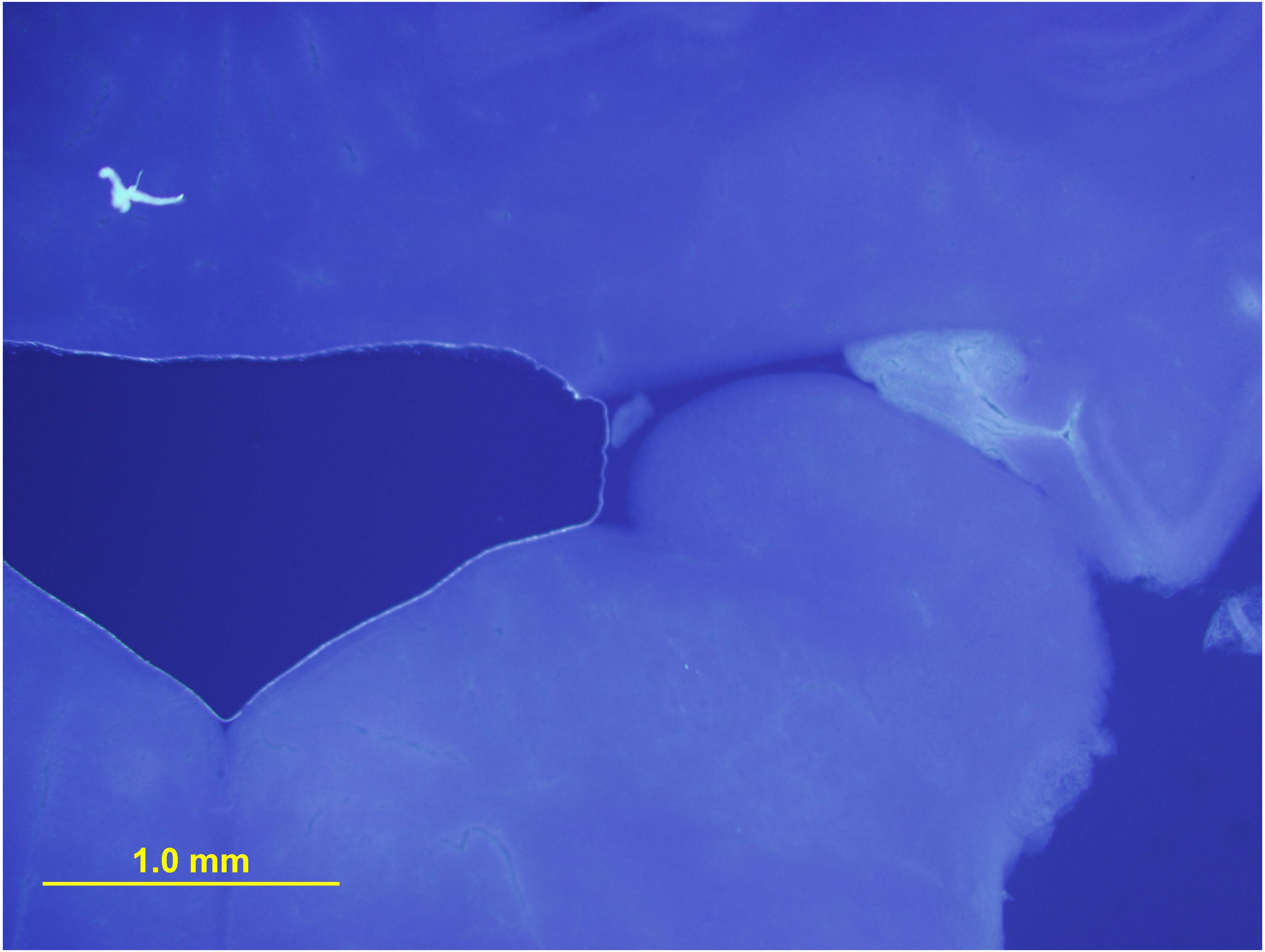
1.0 mm

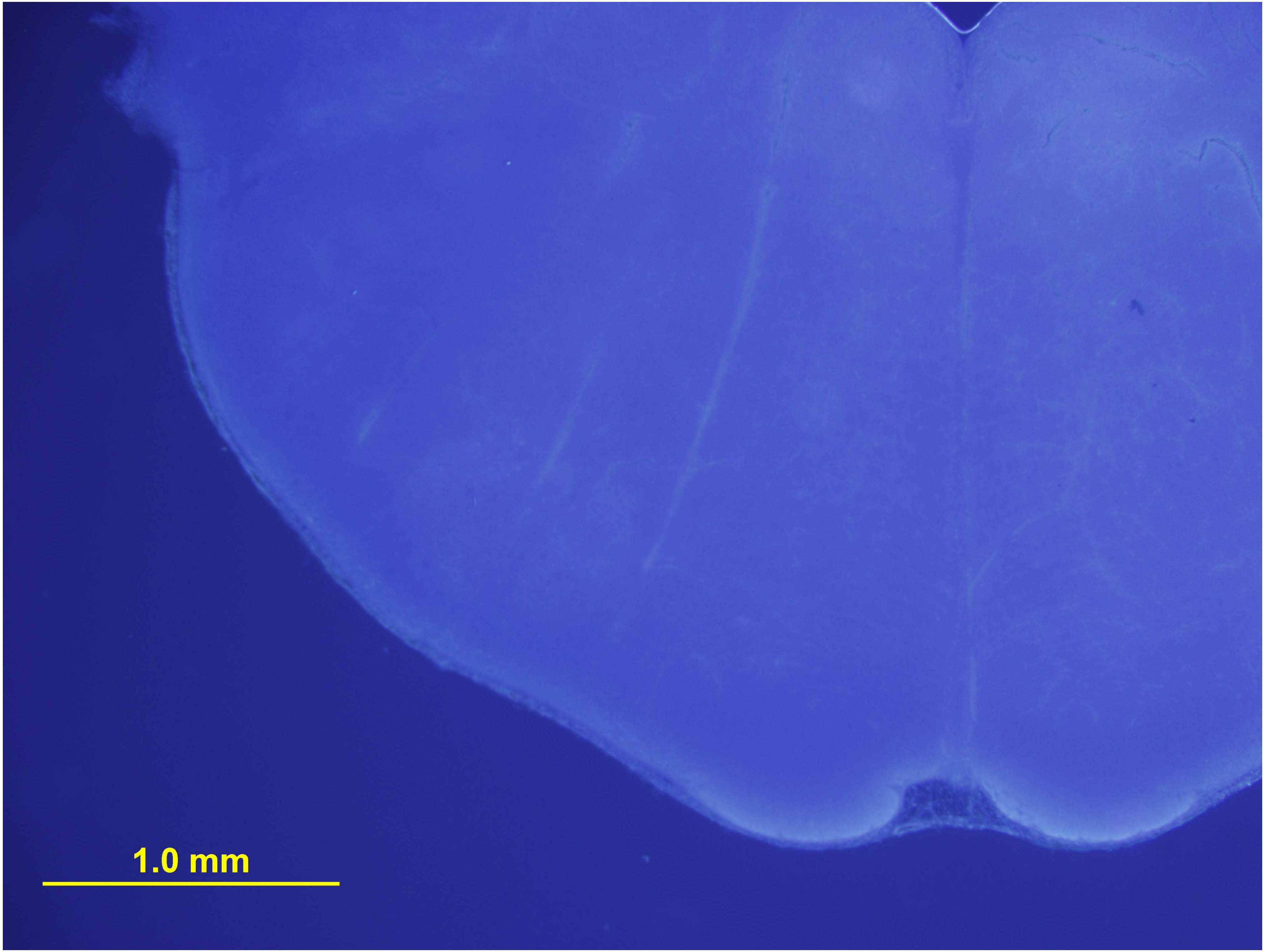
1.0 mm

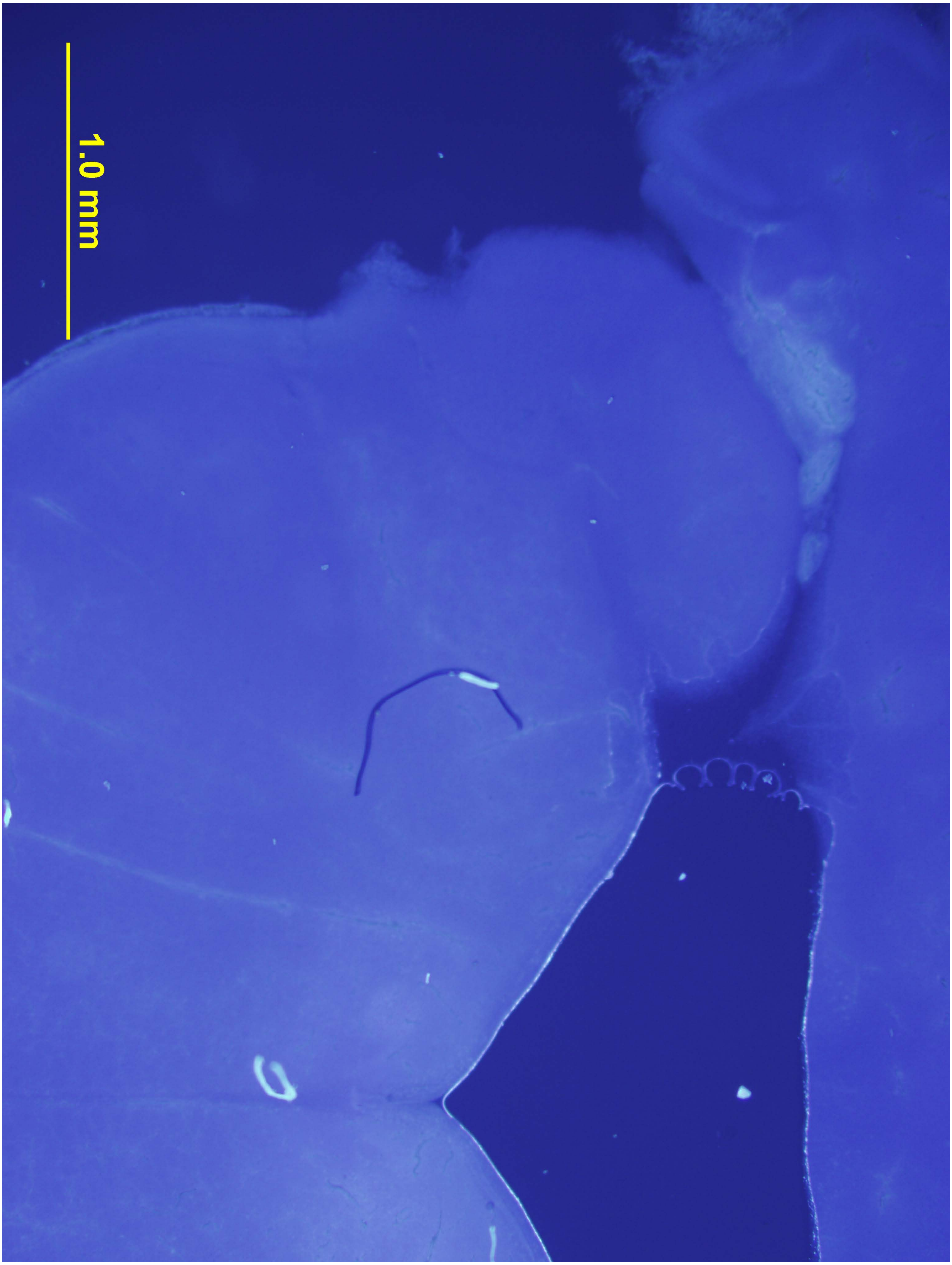
1.0 mm

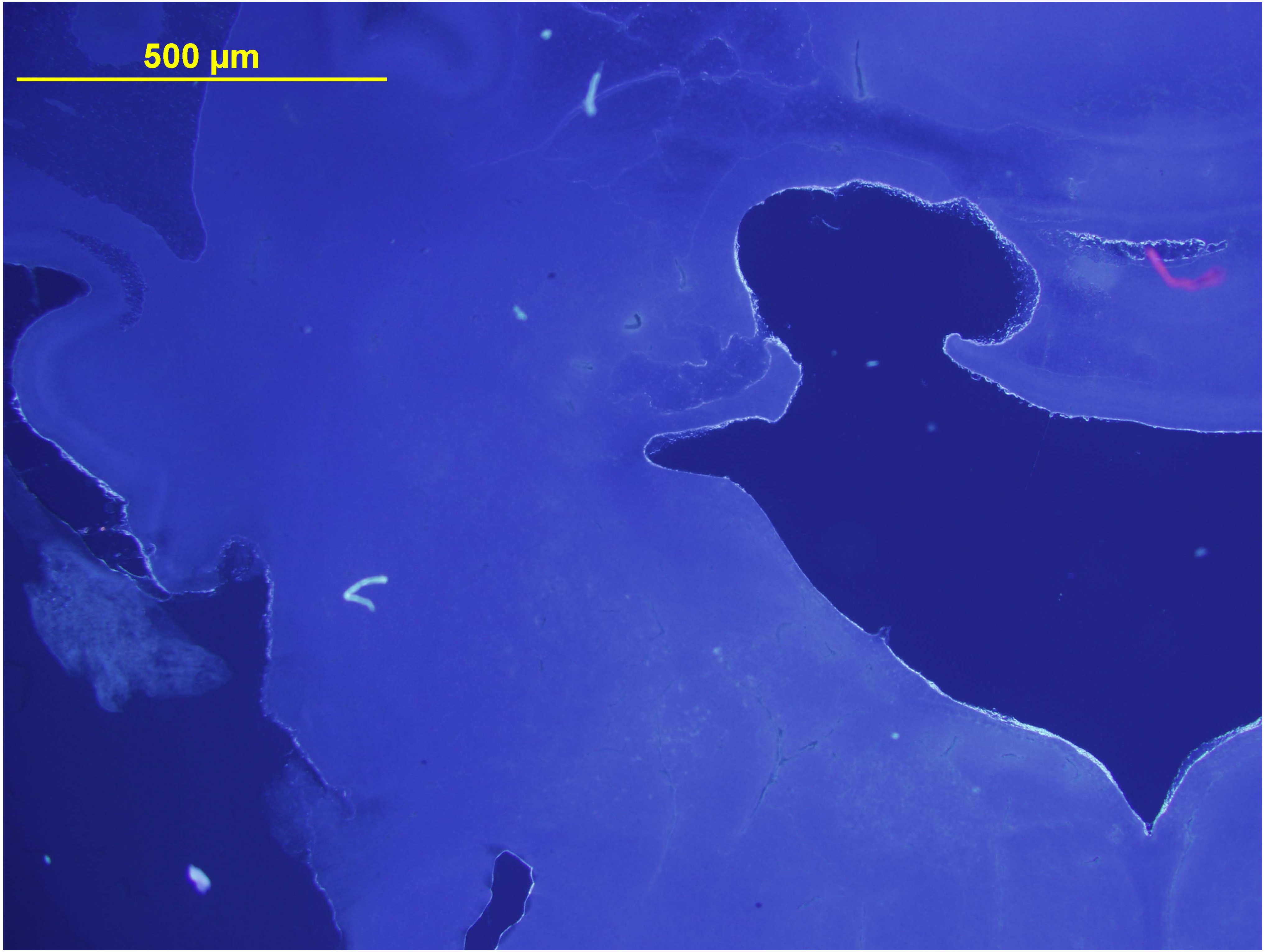
500 µm

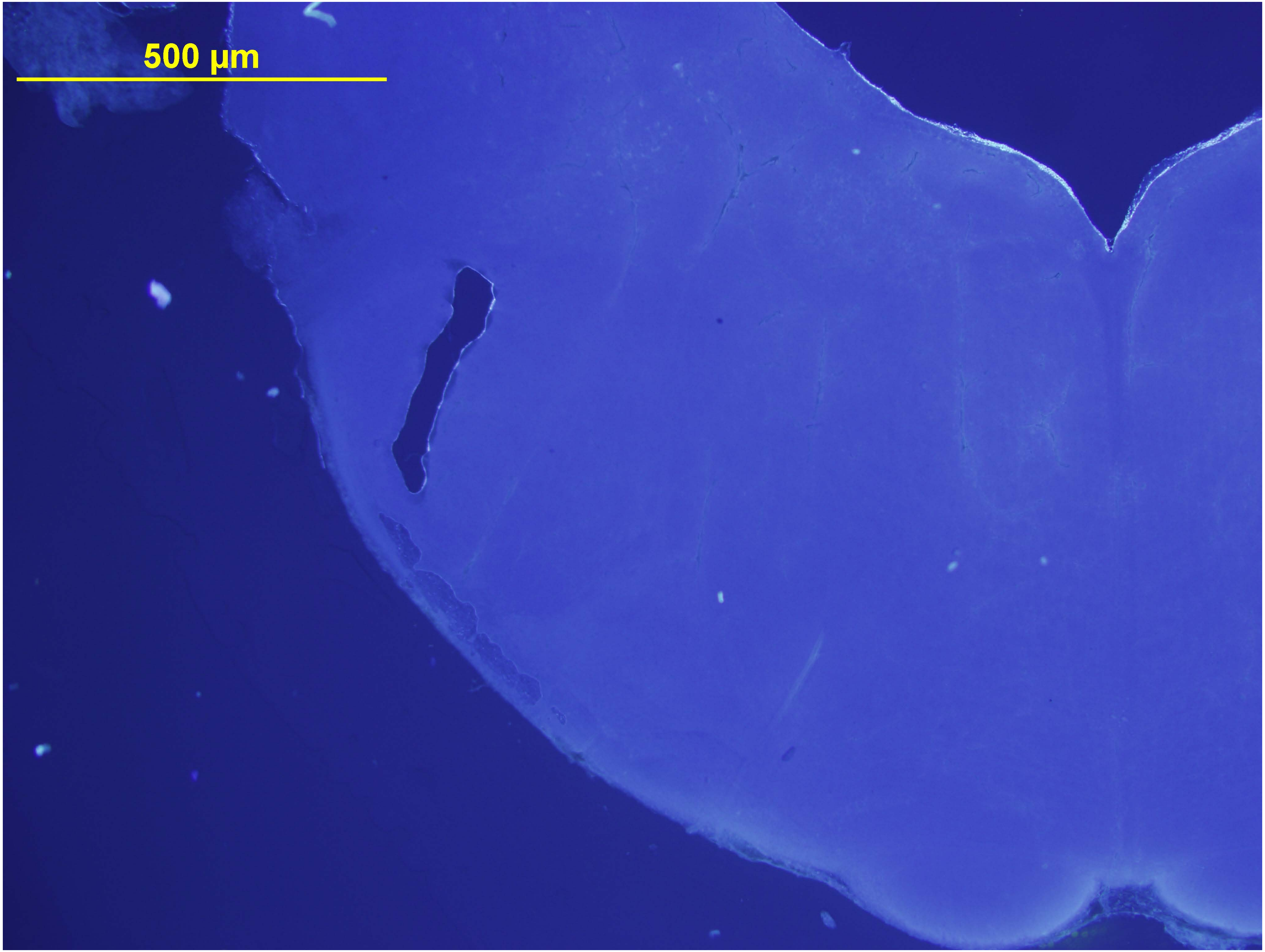
500 µm

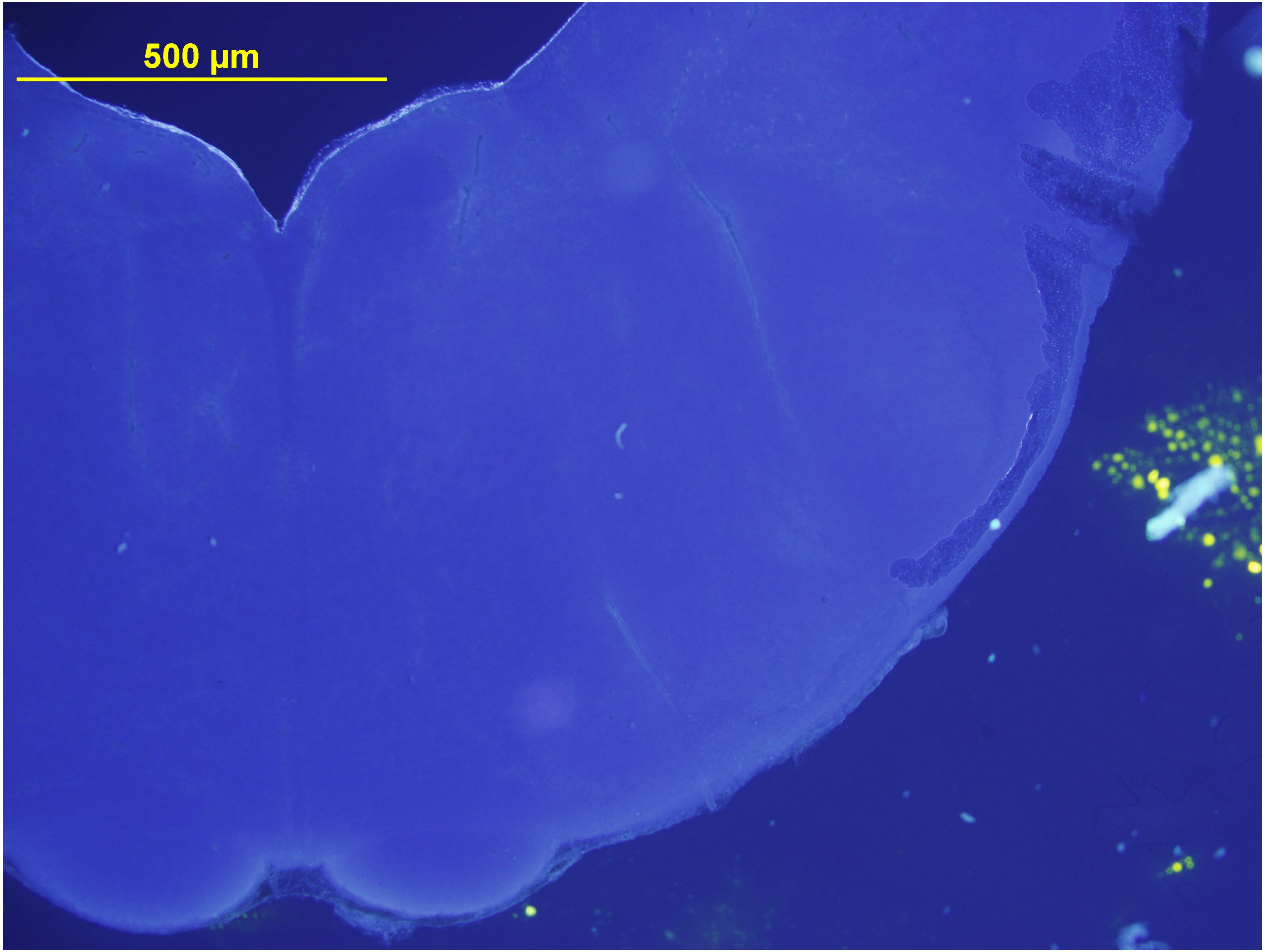
500 µm

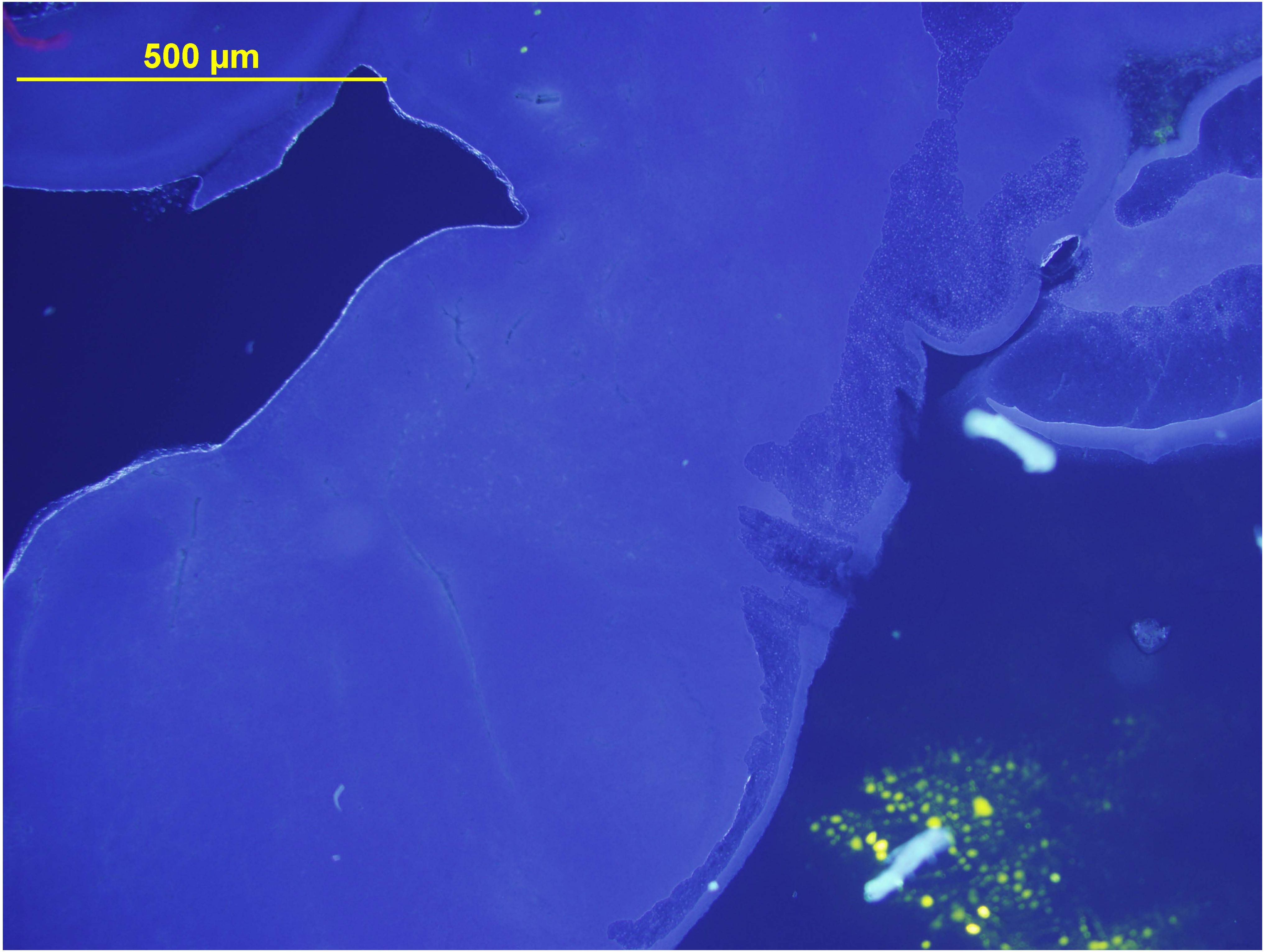
500 µm
