# Supplementary material for: A bipedal mammalian model for spinal cord injury research: The tammar wallaby
Source: F1000Res. 2017 Jun 15;6:921. [Version 1] doi: 10.12688/f1000research.11712.1 (PMC5497825; doi:10.12688/f1000research.11712.1)
Supplement: Peer review history

# Peer review: Referee response for version 1

**DOI:** 10.5256/f1000research.12654.r23514

**Published:** 2017-06-27

**Authors:** Zoltan Varga

**Affiliations:** 1 Zebrafish International Resource Center, University of Oregon, Eugene, OR, USA

I really enjoyed reading this article. It explores whether the tammar wallaby is a suitable model organism to study mechanisms of spinal cord regeration after injury at the mid-thoracic region in pouch young animals. Comparable to previous studies by the Saunders group (e.g. with *Mondoelphis domestica*), this study establishes a critical "permissive" period for wallabies after birth, after which growth of neurites across the lesion and behavioral recovery of hopping and swimming can not occur any longer. Importantly - the tammar wallaby might be a more representative bipedal species to humans because it probably relies less on sensory feedback (as opposed to quadrupeds) for the recovery of locomotor functions after injury.  Therefore, therapeutic discoveries in this species will be more relevant for human spinal cord injuries.

 The article is very thoughtfully written, data are well presented and carefully discussed. The study is scientifically sound. I have no doubt that it will find the interest of a wide audience. A few (very minor) comments, suggestions.

1. The perceived shortcomings of the wallaby model at this time (problematic survival of P40/P60 operated animals), could be overcome by appropriate husbandry refinements and changes in post-operative care. In my opinion the wallaby is an excellent test animal and this article lays important ground work for future studies.
2. PY (pouch young) should be defined at first mention in the main text.
3. It would be helpful to approximately indicate how far rostral and caudal from the lesion site sections B,C, and D were taken in Figure 3.
4. Considering the question of suitability of the wallaby for bipedal recovery after SCI and the role of sensory feedback in the process: what is known about the anatomy and connectivity of spinal cord sensory connections and how wallabies compare e.g. to dorsal spinal cord sensory tracts in *M.* *domestica* or human? Is the absence of Oregon Green labeling rostral to the lesion (after the recovery period) indicative of the absence of sensory feedback connections (compared to quadrupeds)?

I have read this submission. I believe that I have an appropriate level of expertise to confirm that it is of an acceptable scientific standard.

# Peer review: Referee response for version 1

**DOI:** 10.5256/f1000research.12654.r23512

**Published:** 2017-06-26

**Authors:** Helen Marie Bramlett

**Affiliations:** 1 Department of Neurological Surgery, University of Miami, Miami, FL, USA

This manuscript describes the use of the tammar wallaby as a model for use in spinal cord injury research.  The uniqueness of this animal is the fact that it is a bipedal mammal similar to humans when assessing gait either through walking (hopping) or swimming.  Additionally, the tammar wallaby has similar properties in regeneration as the authors’ have previously shown in *Monodelphis domestica*. Several outcomes measures are used including behavioral assessment as well as fluorescent tracing to demonstrate regeneration.  These outcomes clearly show that positive results are only seen when the SCI is done early on in life during a “permissive stage” of regrowth and not during the “non-permissive” stage of the animal.

**Specific Comments:**

1. This is a well-written manuscript and provides compelling evidence for the use of the tammar wallaby as a model for SCI in a bipedal animal.
2. Although limitations are discussed including this one, the survivability of animals in this model for assessment is a big issue for the use of the tammar wallaby.  This will have to be addressed and improved in order for the use of this animal as a valid model for SCI regeneration.
3. However, the use of the tammar wallaby in future studies is well-supported based on the data and conclusions in the paper.

I have read this submission. I believe that I have an appropriate level of expertise to confirm that it is of an acceptable scientific standard.
